# Accurate Biomolecular Structures by the Nano-LEGO Approach: Pick the Bricks and Build Your Geometry

**DOI:** 10.1021/acs.jctc.1c00788

**Published:** 2021-10-20

**Authors:** Giorgia Ceselin, Vincenzo Barone, Nicola Tasinato

**Affiliations:** Scuola Normale Superiore, Piazza Dei Cavalieri 7, I-56126 Pisa, Italy

## Abstract

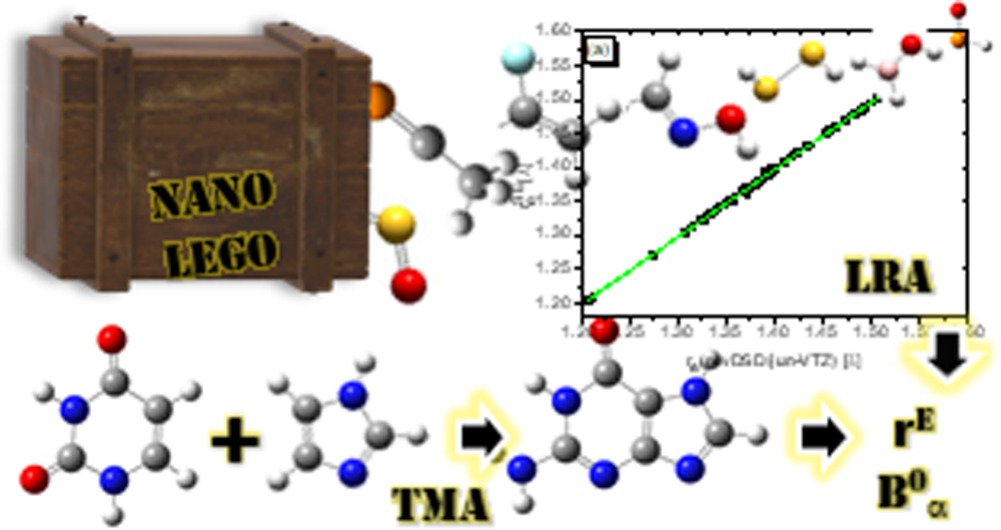

The determination of accurate equilibrium molecular structures plays a fundamental role for understanding many physical–chemical properties of molecules, ranging from the precise evaluation of the electronic structure to the analysis of the role played by dynamical and environmental effects in tuning their overall behavior. For small semi-rigid systems in the gas phase, state-of-the-art quantum chemical computations rival the most sophisticated experimental (from, for example, high-resolution spectroscopy) results. For larger molecules, more effective computational approaches must be devised. To this end, we have further enlarged the compilation of available semi-experimental (SE) equilibrium structures, now covering the most important fragments containing H, B, C, N, O, F, P, S, and Cl atoms collected in the new SE100 database. Next, comparison with geometries optimized by methods rooted in the density functional theory showed that the already remarkable results delivered by PW6B95 and, especially, rev-DSDPBEP86 functionals can be further improved by a linear regression (LR) approach. Use of template fragments (taken from the SE100 library) together with LR estimates for the missing interfragment parameters paves the route toward accurate structures of large molecules, as witnessed by the very small deviations between computed and experimental rotational constants. The whole approach has been implemented in a user-friendly tool, termed nano-LEGO, and applied to a number of demanding case studies.

## 1 Introduction

The knowledge of detailed molecular geometries in the gas phase is the mandatory prerequisite for the study of their physical–chemical properties and for the disentanglement of stereo-electronic, vibrational, and environmental effects that ultimately define the overall experimental observables.^1−3^ Moreover, accurate geometries of isolated molecules provide the best benchmarks for the validation of different quantum mechanical (QM) approaches^4−9^ and for the development of accurate force fields to be used in molecular mechanics (MM)^10^ or multi-level QM/MM models for the study of systems too large to be amenable to the most accurate QM studies. Unfortunately, the determination of accurate equilibrium geometries for molecules of increasing size is not straightforward at all from both experimental and theoretical viewpoints. Theoretically, accurate equilibrium structures can be computed by means of high-level post-Hartree–Fock approaches, with the coupled-cluster model including single and double excitations together with a perturbative inclusion of triples, CCSD(T), being the so-called “gold standard” for molecules not involving too strong static correlation effects.^11^ However, this approach shows a very unfavorable scaling with the dimension of the investigated system, especially when complete basis set extrapolation and core–valence contributions are taken into account. Although more effective composite methods have been devised (e.g., the different flavors of the so-called “cheap” scheme^3,12^), the size of the systems amenable to such accurate studies remains limited.

On the other hand, the analysis of high-resolution spectra (with rotational spectroscopy being the technique of reference in the present context) provides the spectroscopic parameters for the vibrationally averaged structure of one or more vibrational states. The direct experimental outcomes are the rotational constants, which are proportional to the inverse of the inertia moments in the Eckart frame. Since they depend on both the coordinates and the masses of the atoms in the molecule, measurements performed for a sufficient number of isotopologues provide the information needed for determining all the averaged geometrical parameters of the corresponding vibrational states. In order to move to the equilibrium configuration, however, vibrational contributions need to be considered and the rotational constants of the equilibrium geometry have to be employed in the fitting procedure. Experimentally, this means that accurate spectroscopic parameters must be determined in at least one excited vibrational level of each molecule’s normal mode,^13^ which is affordable only for the simplest systems characterized by a reduced number of accessible (i.e., lying at relatively low energies) and nearly isolated (i.e., not involved in strongly resonant systems^14^) fundamental vibrational levels.

In the majority of cases, therefore, a pure experimental route is not practicable. In this connection, the so-called semi-experimental (SE) approach^2,15,16^ represents the best method for obtaining accurate equilibrium structures for all but the smallest (two, three atoms) molecules. In order to exploit this method for semi-rigid molecules, second-order vibrational perturbation theory (VPT2) comes into play, providing explicit expressions of the vibrational corrections to the rotational constants in terms of second and semi-diagonal third-order derivatives of the potential energy with respect to normal modes.^17,18^ The situation is particularly favorable because the sum of the corrections issuing from the different normal modes (contrary to the individual terms) is devoid from any possible resonance and, thanks to a fortuitous but very general error compensation, can be computed with great accuracy with not too-sophisticated QM approaches (e.g., MP2, hybrid, or double-hybrid density functionals). Additional electronic contributions can also be taken into account, but their role is negligible except in very peculiar cases.^19^ We have thus at our disposal a very accurate technique for determining equilibrium geometries whenever a sufficient number of isotopic substitutions are available for the studied molecular system. On these grounds, a data set of about 60 SE equilibrium structures for molecules containing H, C, N, O, F, S, and Cl atoms has been recently built.^19−21^ In the meantime, it has been shown that last-generation hybrid (PW6B95) and double-hybrid (rev-DSDPBEP86) functionals in conjunction with partially augmented basis sets (jul-cc-pVDZ and jun-cc-pVTZ, respectively) provide improved results for several spectroscopic properties^22^ with respect to the B3LYP and B2PLYP models used in the previous compilation. Furthermore, it would be interesting to extend the panel of available structures to other important moieties and to systems also containing B and P atoms. To this end, after determining a number of new SE equilibrium structures, we have also added other systems available in the literature, thus obtaining the new SE100 database. Most building blocks of biomolecules are present in this new database, which will be continuously upgraded including additional moieties.

It follows from the above discussion that on one side, the SE method is the most effective approach for determining accurate equilibrium geometries and on the other side, it is dependent on the availability of experimental rotational constants for a sufficient number of isotopologues. Actually, this may represent the main hurdle to the exploitation of the method given the intrinsic issues in measuring the high-resolution spectra of each singly isotopic substituted species of a molecule, with the deuteration of carbon atoms representing one of the most daunting tasks. With the aim of overcoming the limitations of the SE approach related to the lack of isotopic substitutions and providing cost-effective computational methods to be confidently used for medium to large molecular systems for which coupled-cluster approaches are too demanding, the linear regression (LR) and template molecule approaches (hereafter LRA and TMA, respectively) have been proposed.^19,20^ In the present work, the large SE100 database has been used to build reliable and robust LRs for the key geometrical parameters capable of correcting PW6B95 and rev-DSDPBEP86 equilibrium geometries for systematic errors, thus leading to equilibrium geometries, whose accuracy rivals that of the most refined (and costly) QM approaches. Equipped with this new tool, we have been next able to deal with larger systems for which a full set of isotopic substitutions is not available so that some experimental parameters must be taken from QM computations. In this connection, we will show that integration of LRA and TMA (i.e., the direct transfer of correction to geometrical parameters from suitable fragments of the molecular system at hand) and least square fitting employing the predicate approach (also called the mixed regression model)^23,24^ allow the study of large systems of current technological and/or biological interest. In this respect, a fully black-box tool, referred to as nano-LEGO, has been implemented, which, starting from an initial (even rough) structure of the molecular system, leads to a very accurate equilibrium geometry and, when needed, to accurate rotational constants to be directly compared with their experimental counterparts.

## 2 Methodology and Computational Details

Quantum chemical calculations rooted into density functional theory (DFT) were carried out by using hybrid and double-hybrid density functionals of the last generation, which are considered the best performing for geometries and spectroscopic parameters according to a very recent benchmark.^22^ Among hybrid functionals, the PW6B95 meta exchange–correlation functional of Zhao and Truhlar^25^ was selected in conjunction with the jul-cc-pVDZ double-ζ basis set.^26^ The rev-DSDPBEP86 double-hybrid density functional, recently proposed by Martin and co-workers,^27^ was employed together with the jun-cc-pVTZ basis set.^26^ Indeed, triple-ζ basis sets in conjunction with the B2PLYP double-hybrid functional^28^ have been demonstrated to provide accurate predictions of geometries, rotational spectroscopic parameters, and vibrational properties.^7,20,29−32^ Also, if not explicitly indicated, both PW6B95 and rev-DSDPBEP86 were always augmented for dispersion contributions by means of the Grimme’s DFT-D3 scheme^33^ with Becke-Johnson damping,^34^ even if the bare PW6B95 functional can already provide a satisfactory description of dispersion forces.^35^ Since tight d functions are important for a quantitative representation of the electronic structure of second-row elements, partially augmented basis sets, namely, jul-/jun-cc-pV(n+d)Z with n = D, T, including an additional set of d functions, were employed for phosphorous, sulfur, and chlorine atoms. These basis sets were downloaded from the Basis Set Exchange library.^36^ At each level of theory, geometries were first optimized and then harmonic vibrational frequencies were computed by means of analytical Hessian matrices.^29^ The cubic force constants were calculated by numerical differentiation of analytic quadratic force constants and then employed to compute vibrational contributions to rotational constants in the framework of VPT2.^17,18,37,38^ We recall that while specific vibrational contributions can be plagued by resonances, their sum can be written in a resonance-free form. All DFT calculations were carried out by using the Gaussian 16 suite of programs,^39^ which was also employed for the perturbative treatment of the anharmonic force field in the framework of a general VPT2 engine.^38,40^ Since rev-DSDPBEP86 is not among the Gaussian built-in functionals, it has been defined by setting proper IOP flags on top of the DSDPBEP86 functional. In order to obtain the SE equilibrium structure of a molecule, the SE rotational constants of a set of isotopologues were employed in a nonlinear least squares fitting procedure to refine the structural parameters. SE rotational constants (*B*_α_^SE^, with α = *a*, *b*, *c*, representing the principal inertial axis) were obtained by removing vibrational contributions (*ΔB*_α_^vib^) from the corresponding ground-state rotational constants (*B*_α_^0^) measured experimentally
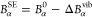
1where Δ*B*_α_^vib^ was obtained by VPT2 applied to semi-diagonal cubic force constants evaluated at the rev-DSDPBEP86/jun-cc-pV(T+d)Z/jul-cc-pVDZ level. The MSR software^41^ was used for applying the SE procedure.

In TMA, improved estimates of the different geometrical parameters (*r*_e_) of the target molecule are obtained from the corresponding parameters issuing from geometry optimization (*r*_opt_) as
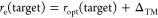
2with
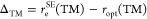
3where *r*_e_^SE^(TM) and *r*_opt_(TM) are, respectively, the structural parameter of the SE geometry (taken from the SE100 database) and calculated theoretically at the same level as that of the target molecule, of a reference molecule (i.e., the template) which shares structural similarities with the target one.

The LR approach replaces the Δ_TM_ correction by an estimate (Δ_LR_) based on an LR model
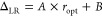
4so that
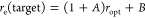
5

The parameters *A* and *B* depend only on the atomic numbers of the involved atoms and are obtained by a statistical analysis of a large number of molecules (in the present case, those contained in the SE100 data set). Of course, the equilibrium geometrical parameters estimated by TM and/or LR approaches can be either fixed in the least square refinement leading to SE structures or employed as reliable predicates in a mixed regression refinement.

## 3 Results and Discussion

### 3.1 SE100 Database

**Figure 1:**
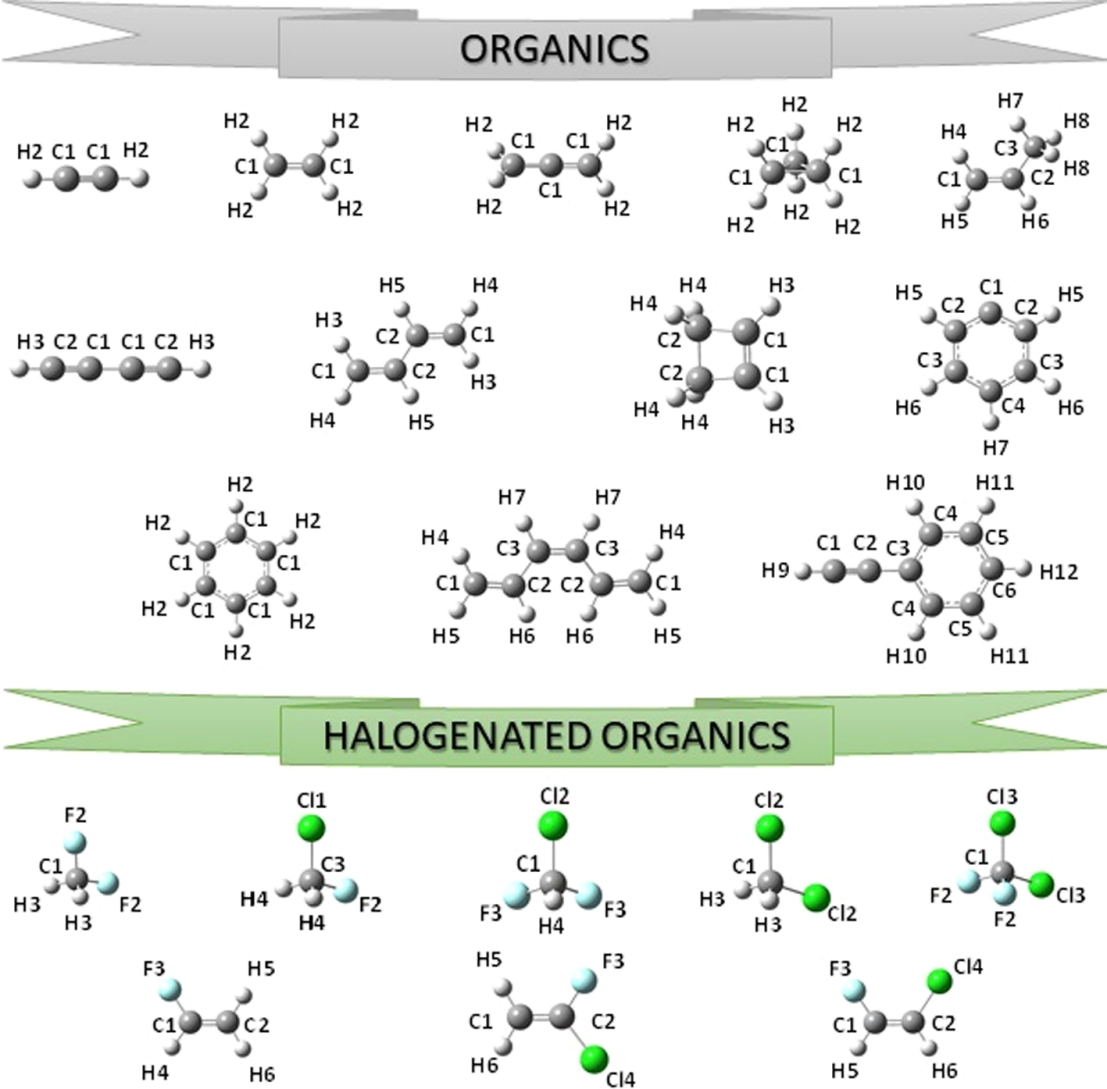
Structure and atom labeling of the organic and halogenated organic molecules previously included in the SE100 database.

**Figure 2:**
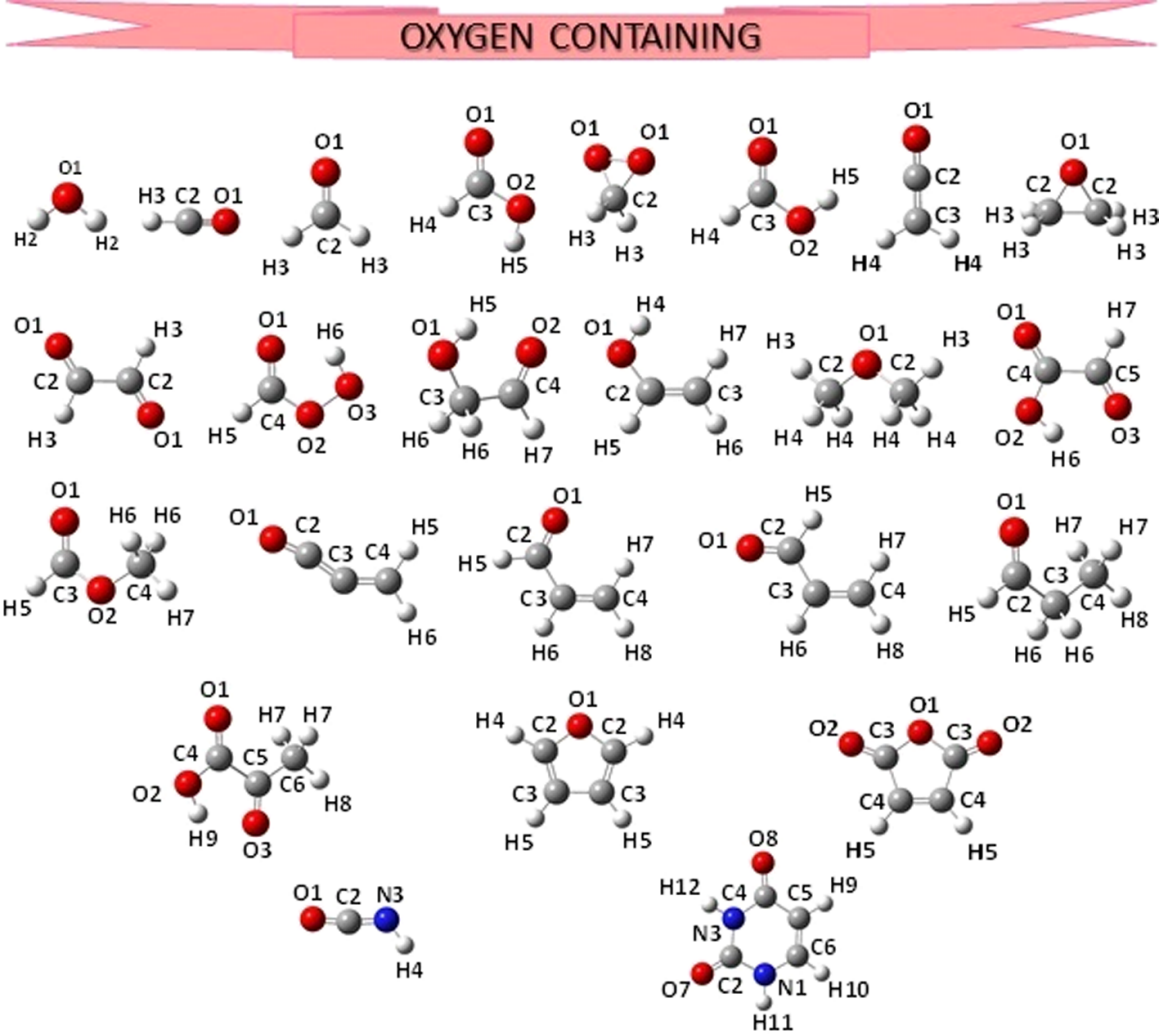
Structure and atom labeling of the oxygen-containing molecules previously included in the SE100 database.

**Figure 3:**
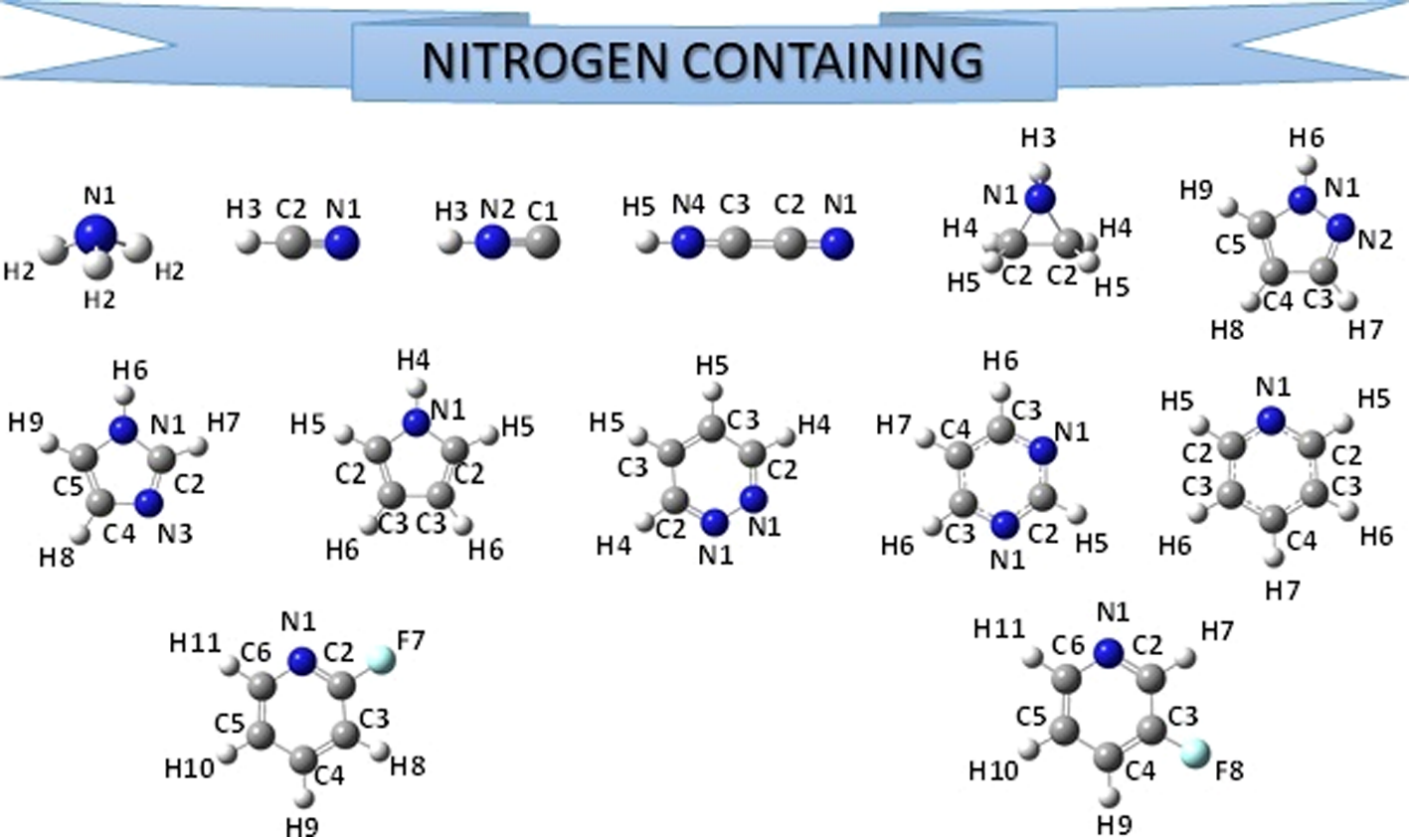
Structure and atom labeling of the nitrogen-containing molecules previously included in the SE100 database.

**Figure 4:**
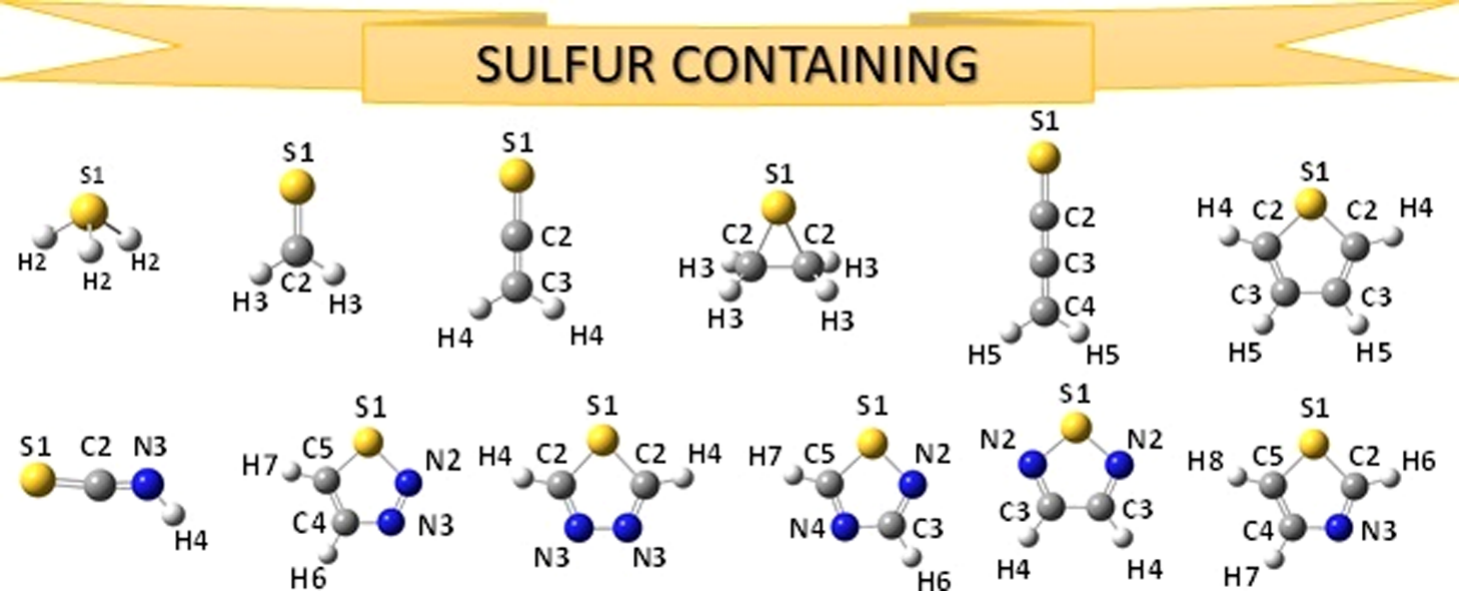
Structure and atom labeling of the sulfur-containing molecules previously included in the SE100 database.

LRA was originally developed for the B3LYP/SNSD^42−44^ model chemistry based on a set of 47 SE equilibrium structures,^19^ which was later extended by new SE equilibrium geometries and employed to parameterize LRA for the B2PLYP/cc-pVTZ level of theory.^20^ More recently, sulfur-containing species have been included,^21^ resulting in a database collecting the equilibrium geometries of 69 molecules, reported in Figures 1–4, which were obtained by using either B3LYP/SNSD (B3se database) or B2PLYP/cc-pVTZ (B2se database) vibrational contributions to correct ground-state rotational constants.

**Figure 5:**
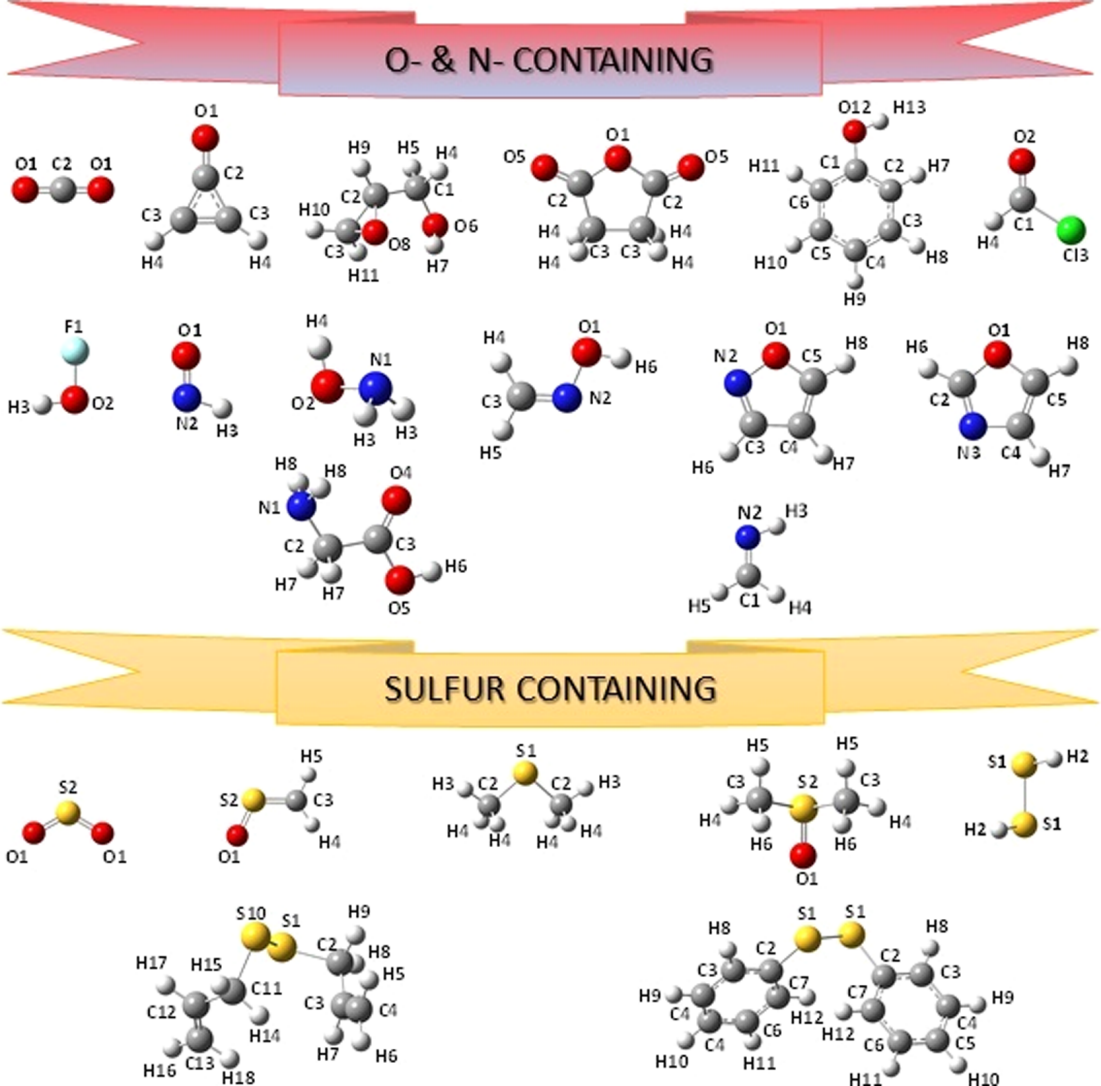
Structure and atom labeling of the new oxygen-, nitrogen-, and sulfur-containing molecules included in the SE100 database.

**Figure 6:**
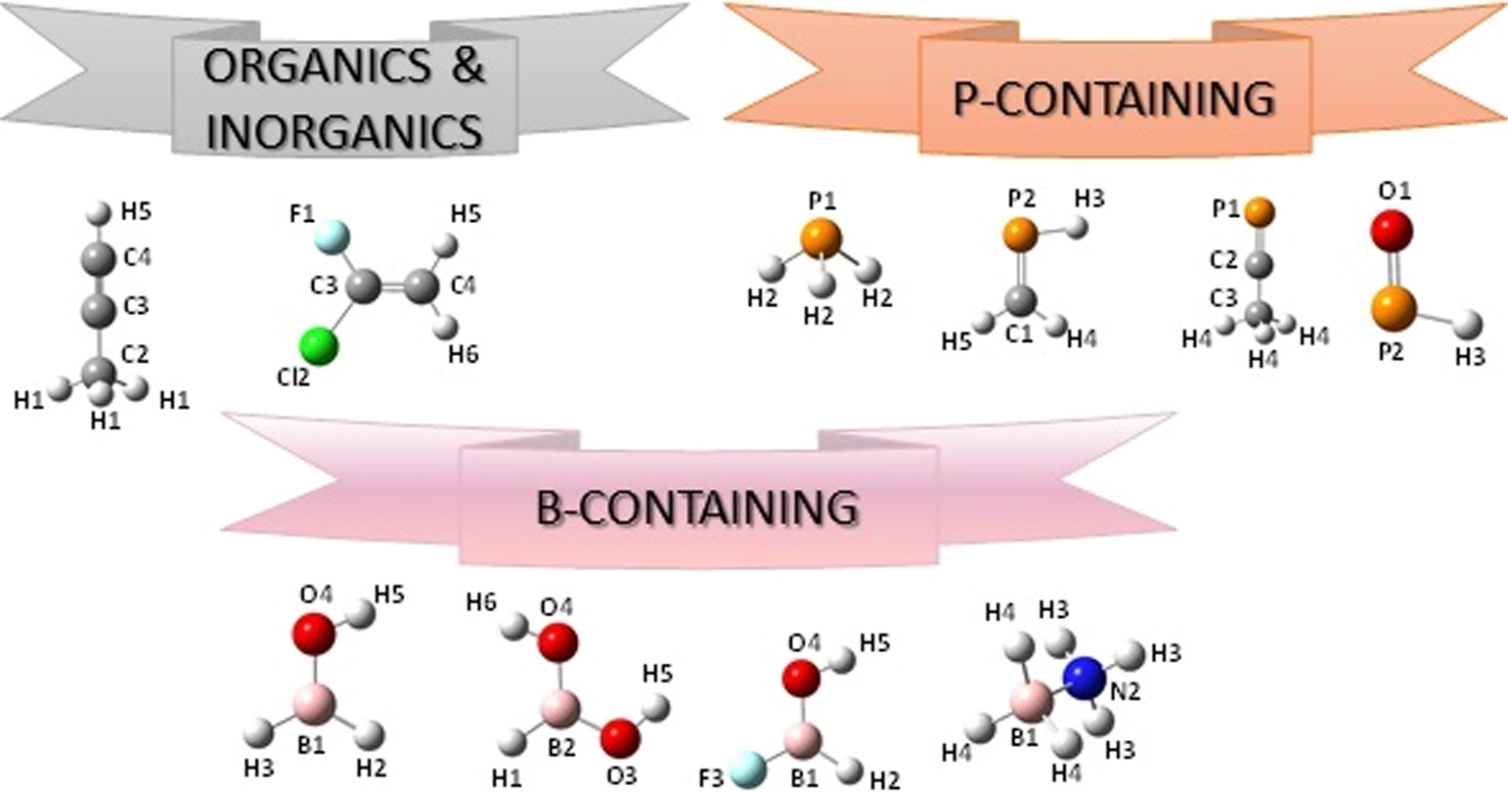
Structure and atom labeling of the new organic and inorganic species and of phosphorous- and boron-containing molecules included in the SE100 database.

The first step toward the definition of LRA for application to biochemical building blocks was the enrichment of the structural database with 31 molecules which are shown in Figures 5 and 6, while their SE equilibrium geometries can be found in Table S1 of Supporting Information. Attention has been primarily focused in extending the database with species formed by biochemically relevant elements. Specifically, in addition to propyne,^45^ 12 nitrogen- and/or oxygen-bearing molecules have been added (HNO,^46^ NH_2_OH, CH_2_NH,^47^ CH_2_NOH, CO_2_,^2^ succinic anhydride,^48^ phenol, oxazole, isoxazole, glycidol,^49^ cyclopropenone,^50^ and glycine^41^) and the number of sulfur compounds has been increased substantially with the inclusion of sulfur dioxide,^7^ dimethyl sulfide,^51^ dimethyl sulfoxide,^52^ sulfinylmethane,^53^ hydrogen disulfide,^54^ diallyl disulfide,^55^ and diphenyl disulfide,^56^ with the last three molecules involving a sulfur–sulfur bridge. Since phosphorus is a biologically relevant element not represented in the database, efforts have been made to overcome this limit, resulting in the inclusion of the PH_3_,^2^ CH_3_CP,^57^ CH_2_PH,^47^ and HPO^58^ molecules for which an accurate SE equilibrium geometry has been worked out in the literature. Furthermore, four boron-containing molecules have been introduced (BH_2_OH, BH(OH)_2_, BHFOH, and BH_3_NH_3_^59^), with the halogenated organics (1,1-ClFCCH_2_,^60^ OCHCl^61^) as well as the inorganic compound HOF.^2^ In the following, new SE equilibrium structures determined in the present work are discussed first; then, the performance of the PW6B95 and rev-DSDPBEP86 density functionals are described, with their LRA parameterization presented showing the improvements it delivers over the bare functionals for different classes of molecules and structural parameters.

#### 3.1.2 New Semi-Experimental Equilibrium Geometries

The SE equilibrium structures of CH_2_NOH, BH_2_OH, BH(OH)_2_, BHFOH, oxazole, isoxazole, and phenol have been determined in the present work by correcting the ground-state rotational constants measured experimentally for a set of isotopologues through vibrational corrections evaluated at the rev-DSDPBEP86/jun-cc-pVTZ level of theory. In addition, some tests have been carried out by using α vibration–rotation interaction constants computed at the PW6B95/jul-cc-pVDZ level of theory obtaining, within the quoted errors, the same results as the double-hybrid density functional which are listed in Table 1.

**Table 1:** Semi-Experimental, rev-DSDPBEP86, and PW6B95 Equilibrium Geometries of the New Molecules Added to the SE100 Database^a^

molecule	parameter	SE	rev-DSDPBEP86	PW6B95
BHFOH	*r*(B–H)	1.189385(49)	1.1932	1.1973
	*r*(B–F)	1.31961(15)	1.3265	1.3390
	*r*(B–O)	1.34626(15)	1.3537	1.3478
	*r*(O–H)	0.95735(16)	0.9603	0.9590
	α(HBF)	119.381(44)	119.37	119.14
	α(HBO)	123.459(44)	123.48	124.38
	α(HOB)	112.714(18)	112.76	113.07
BH_2_OH	*r*(B–H1)	1.1957(16)	1.1982	1.2044
	*r*(B–H2)	1.1899(16)	1.1926	1.1997
	*r*(B–O)	1.34979(13)	1.3569	1.3524
	*r*(O–H)	0.9558(17)	0.9614	0.9600
	α(HBH)	122.84(15)	122.77	123.02
	α(HBO)	119.80(23)	120.49	120.35
	α(HOB)	112.90(19)	112.94	113.18
BH(OH)_2_	*r*(B2–H1)	1.189723(80)	1.1936	1.1986
	*r*(B–O3)	1.35353(19)	1.3608	1.3574
	*r*(B–O4)	1.36364(17)	1.3709	1.3674
	*r*(O3–H5)	0.96034(15)	0.9634	0.9621
				
	*r*(O4–H6)	0.95625(24)	0.9596	0.9580
	α(H1B2O3)	118.527(94)	118.534	118.658
	α(O3B2O4)	119.1251(41)	119.171	118.877
	α(B2O3H5)	111.9132(59)	111.956	111.932
	α(B2O4H6)	116.223(77)	116.33	116.52
cyc-H_2_C_3_O	*r*(C=O)	1.20043(13)	1.2044	1.2024
	*r*(C2–C3)	1.42887(42)	1.4350	1.4298
	*r*(C–H)	1.078173(54)	1.0814	1.0862
	α(CCO)	151.9272(23)	151.93	151.91
	α(CCH)	153.7200(64)	153.78	153.86
H_2_C=NOH	*r*(N–O)	1.39899(35)	1.4005	1.3867
	*r*(C=N)	1.27112(42)	1.2728	1.2679
	*r*(C–H1)	1.08425(47)	1.0873	1.0918
	*r*(C–H2)	1.07952(64)	1.0814	1.0858
	*r*(O–H3)	0.9627(12)	0.9627	0.9607
	α(CNO)	110.548(15)	110.87	111.31
	α(H1CN)	122.238(43)	122.47	122.54
	α(H2CN)	116.223(77)	116.33	116.52
	α(H3ON)	102.65(11)	102.61	103.41
cyc-C_3_H_3_NO	*r*(O1–C2)	1.35097(30)	1.3548	1.3487
	*r*(C2=N3)	1.28819(27)	1.2926	1.2897
	*r*(C4–N3)	1.39268(30)	1.3945	1.3848
	*r*(C4=C5)	1.34934(20)	1.3531	1.3528
	*r*(C2–H2)	1.07481(26)	1.0774	1.0800
	*r*(C4–H4)	1.07433(26)	1.0767	1.0804
	*r*(C5–H5)	1.07300(58)	1.0752	1.0785
	α(NCO)	115.076(19)	114.93	114.65
	α(CNC)	103.884(17)	103.94	104.13
	α(CCN)	109.003(20)	109.04	109.06
	α(HCO)	116.832(20)	116.74	116.88
	α(HCN)	121.867(61)	121.93	122.06
	α(HCC)	135.145(61)	135.16	135.21
iso-cyc-C_3_H_3_NO	*r*(O1–N2)	1.39274(57)	1.3930	1.3802
	*r*(N2=C3)	1.30706(85)	1.3121	1.3064
	*r*(C3–C4)	1.42101(61)	1.4213	1.4191
	*r*(C4=C5)	1.3515(10)	1.3577	1.3564
	*r*(C3–H3)	1.07590(50)	1.0793	1.0831
	*r*(C4–H4)	1.07311(54)	1.0759	1.0795
	*r*(C5–H5)	1.07556(90)	1.0777	1.0813
	α(CNO)	105.518(42)	105.41	105.48
	α(CCN)	112.159(57)	112.20	112.16
	α(CCC)	102.986(54)	103.003	102.79
	α(HCN)	118.50(13)	118.71	118.78
	α(H7C4C3)	128.44(13)	128.77	128.87
	α(H8C5C4)	133.52(24)	133.64	133.62
C_6_H_5_OH	*r*(C1–C2)	1.39197(64)	1.3941	1.3927
	*r*(C2–C3)	1.39010(43)	1.3937	1.3910
	*r*(C3–C4)	1.39018(43)	1.3921	1.3902
	*r*(C4–C5)	1.39170(43)	1.3951	1.3929
	*r*(C5–C6)	1.38882(37)	1.3943	1.3926
	*r*(C1–O1)	1.36386(28)	1.3681	1.3632
	*r*(O1–H7)	0.95939(50)	0.9624	0.9600
	*r*(C2–H2)	1.08363(33)	1.0856	1.0888
	*r*(C3–H3)	1.08090(22)	1.0836	1.0870
	*r*(C4–H4)	1.07960(16)	1.0826	1.0859
	*r*(C5–H5)	1.08133(23)	1.0836	1.0870
	*r*(C6–H6)	1.07972(30)	1.0828	1.0859
	α(C1C2C3)^fixed^	119.68	119.68	119.65
	α(C2C3C4)^fixed^	120.50	120.50	120.56
	α(C3C4C5)^fixed^	119.30	119.30	119.22
	α(C4C5C6)^fixed^	120.79	120.74	120.83
	α(C2C1O1)^fixed^	122.42	122.45	122.44
	α(C1O1H7)	108.907(19)	108.94	109.43
	α(C3C2H2)	120.507(41)	120.31	120.38
	α(C4C3H3)	120.164(24)	120.17	120.14
	α(C5C4H4)^fixed^	120.37	120.37	120.43
	α(C6C5H5)	119.286(25)	119.28	119.24
	α(C1C6H6)^fixed^	119.05	119.04	119.24

a Bond lengths and angles in Å and °, respectively. Figures in parentheses are standard deviations in the units of the last significant digits. See Figures 5 and 6 for atom labeling.

In the case of formaldoxime, the ground-state rotational constants of the main isotopic species have been recently redetermined by Wu and Tan,^62^ while those of the *cis*-CHDNOH, *trans*-CHDNOH, ^13^CH_2_NOH, CH_2_^15^NOH, and CH_2_N^18^OH isotopologues have been measured by Levine long time ago.^63^ The fits leading to the SE equilibrium geometry have been carried out by using only the *B* and *C* SE rotational constants and the rev-DSDPBEP86/jun-cc-pVTZ geometry as the guess for the equilibrium structure. As expected, due to the lack of isotopic substitutions for the hydrogen atom of the hydroxyl group, the accuracy of the equilibrium values determined for the O–H bond length and the NOH valence angle is unsatisfactory. In order to overcome the problem, the method of predicate observations has been adopted with the set of rotational constants augmented by the theoretical predictions of the two structural parameters. In addition, the error on the C–H2 bond length was about twice that of the C–H1 one, a problem that, after some tests, has been related to the limited accuracy of the experimental rotational constants determined for the ^13^C isotopologue from just two observed transitions. While a reinvestigation of the rotational spectra of the different isotopologues should be encouraged, in the present work, the issue has been solved by reducing the weight of the ^13^CH_2_NOH rotational constants and utilizing a predicate also for the C–H2 bond length. In addition, due to the lack of an accurate theoretical geometry, the equilibrium structure has been computed by using the CCSD(T)/CBS + CV gradient composite scheme^64^ employing the CFOUR package.^65^ The corresponding values of the structural parameters were employed as predicates. The SE equilibrium structure, reported in Table 1, appears well determined, including the O–H bond length and the NOH valence angle, which are affected by slightly larger uncertainties because of the aforementioned lack of deuteration on the OH moiety.

Concerning boronic acid and fluorohydroxyborane, Kawashima et al.^66^ measured the rotational constants of 18 and 10 isotopologues, respectively, and determined both a partial and an effective structure. Although the equilibrium geometries of the two molecules have been recently obtained by CCSD(T)-based composite schemes and the SE equilibrium structure of BHFOH has also been determined, the values obtained for the B–F and B–O lengths as well as the HBF and HBO angles were considered not fully satisfactory.^67^ For this reason, the BHFOH SE equilibrium structure has been redetermined in this work, trying to overcome the limitation due to the lack of isotopic substitution for fluorine by using predicate values for both the B–F bond length and the HBF angle taken from the CCSD(T)/VQZ + MP2[V5Z – VQZ + wCVQZ(ae) – wCVQZ(fc)] structure.^67^ Some tests have also been carried out by avoiding or reducing (to just the B–F bond length or the HBF valence angle) the use of predicates, but the results were quite unsatisfactory. The best SE equilibrium geometry issuing from the use of two predicates shows standard deviations lower than 3 × 10^–4^ Å and 0.09° for bond lengths and angles, respectively.

The SE equilibrium structure of boronic acid is well determined as well, with standard deviations in the range between 0.8–2.4 × 10^–4^ Å and 0.4–9.4 × 10^–2^° for bond lengths and angles, respectively, and furthermore, it is consistent with the theoretical structure computed by Demaison et al.^67^ The SE equilibrium geometry of borinic acid has been obtained from the rotational constants measured for four isotopologues:^68^^11^BH_2_OH, ^10^BH_2_OH, ^11^BD_2_OH, and ^11^BH_2_^18^OH. Due to the lack of isotopic substitution on the hydrogen atom of the hydroxyl group, a preliminary fit has been performed by constraining the O–H distance and the BOH angle to the theoretical value computed at the CCSD(T)/VQZ + MP2[V5Z – VQZ + wCVQZ(*ae*) – wCVQZ(*fc*)] level.^67^ In the next step, the fit has been augmented by using these values as predicate observations resulting, however, in B–H bond lengths determined with a relatively low accuracy. For this reason, in the final fit, predicates have been used also for the B–H distances, obtaining the structure reported in Table 1. The errors on retrieved parameters may appear somewhat large for a SE equilibrium geometry, being around 0.001 Å for bond lengths and 0.5° for valence angles. This behavior however was expected due to the limited number of available experimental data. With this said, the obtained equilibrium geometry appears reliable as also confirmed by the good agreement with the high-level theoretical predictions.^67^

Moving to cyclic molecules, the microwave spectra of all the monosubstituted isotopologues of oxazole were recorded in the ground vibrational state long time ago;^69^ however, the structural fit performed by considering all isotopic species showed a too high uncertainty for the HCO angle and, most importantly, an unrealistically large C4–H4 bond length of 1.092 Å that poorly matches that obtained at the rev-DSDPBEP86 level (1.077 Å) as well as the *r*_s_ structure (1.075 Å)^69^ and appears too different from the remaining C–H distances of the molecule. A fully satisfactory fit has been obtained by excluding the rotational constants of the D4 and D5 isotopologues and using predicates for the C4–H4, C5–H5, HCN, and CCH geometrical parameters, with initial values taken from the rev-DSDPBEP86 structure improved by LRA corrections (see below). As can be seen from the results reported in Table 1, the obtained SE equilibrium geometry appears reliable and well determined, with the standard deviations being around 3 × 10^–4^ Å for bond lengths and between 0.02 and 0.09° for bond angles.

The rotational spectra of isoxazole in natural isotopic abundance were investigated by double-resonance microwave spectroscopy obtaining the ground-state rotational constants of all its isotopologues.^70,71^ The SE equilibrium structure has been obtained by fitting the structural degrees of freedom to the *A* and *B* rotational constants of all isotopic species, reaching a standard deviation of 3 × 10^–3^ amuÅ^2^.

The determination of the structure of phenol has been the subject of great research efforts, on one side because of the intrinsic importance of the molecule in organic synthesis and on the other for understanding the dynamics of the hindered internal rotation of the hydroxyl group. In 1960, the first study dealt with the parent species,^72^ and some years later, the rotational spectra of C_6_H_5_^18^OH, C_6_D_5_OH, and C_6_D_5_OD were analyzed, obtaining the respective rotational constants.^73^ Subsequently, the measurements were focused on the six monodeuterated phenol and mono-^13^C isotopologues.^74,75^ In this work, the SE equilibrium structure has been obtained by a step-wise fitting of the rotational constants of the different isotopic species. At first, the parent and the ^13^C species have been considered and only the C=C bond lengths have been refined, constraining the remaining structural parameters to the rev-DSDPBEP86 values. Next, the rotational constants corresponding to the hydroxyl deuteration have been added and the O–H bond distance varied accordingly. The data for C–H deuteration have been introduced in the fit as well as those corresponding to multiple deuterations, and all the bond lengths but C–O have been refined. Finally, the rotational constants of the ^18^O isotopologue have been introduced, relaxing all the bond lengths. In the next step, also, bond angles have been refined, but the fit has resulted affected by the strong correlation among bond angles, particularly those of the aromatic ring. In order to reduce the correlations, predicate observations have been introduced for C1C2C3, C2C3C4, C3C4C5, C4C5C6, C2C1O1, C5C4H4, and C1C6H6 valence angles, obtaining the structure reported in Table 1 with a standard deviation of 2.4 × 10^–3^ amuÅ^2^.

Finally, the SE structure of cyclopropenone has been redetermined by correcting the ground-state rotational constants recently measured for the six isotopologues^50^ through vibrational corrections obtained at either the rev-DSDPBEP86 or PW6B95 levels of theory. The fit of these rotational constants has converged straightforwardly pointing out the high accuracy and reliability of the underlying experimental data. The structural parameters obtained are coincident with those determined by Müller et al.^50^ but with significantly lower uncertainties, probably due to the different fitting software employed.

### 3.2 Organic Molecules: Placing Carbon and Hydrogen Atoms

**Figure 7:**
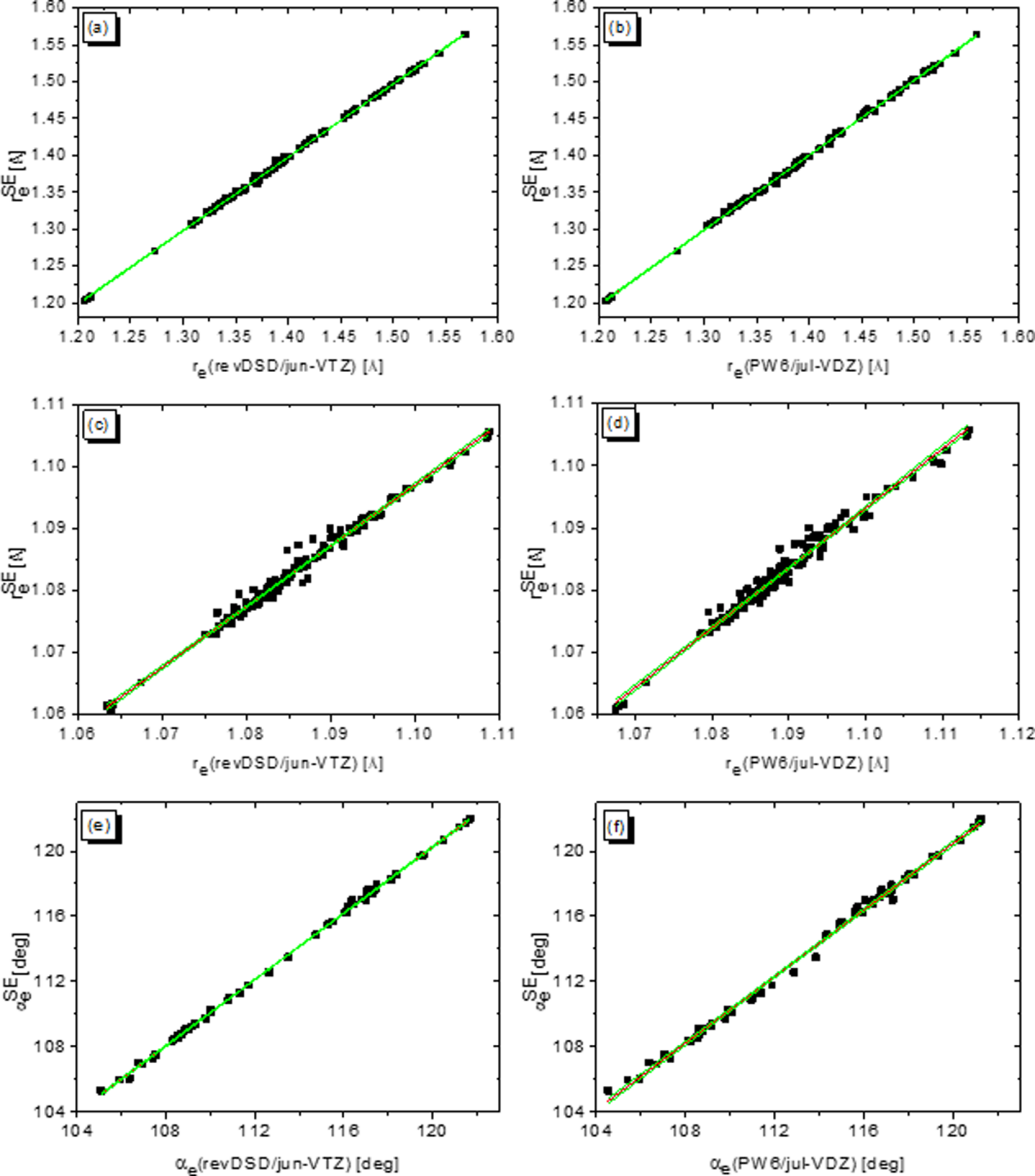
LRA for CC bond lengths at (a) rev-DSDPBEP86/jun-cc-pVTZ and (b) PW6B95/jul-cc-pVDZ levels of theory, for CH bond lengths at (c) rev-DSDPBEP86/jun-cc-pVTZ and (d) PW6B95/jul-cc-pVDZ levels of theory, and for HCH angles at (e) rev-DSDPBEP86/jun-cc-pVTZ and (f) PW6B95/jul-cc-pVDZ levels of theory. Green curves represent 95% confidence intervals.

The first step toward the determination of accurate equilibrium molecular structures for biomolecular building blocks, for which the SE approach cannot be undertaken due to the increasing molecular size, is clearly the building of an accurate carbon backbone. Even more important is the ability to correctly predict C–H bond lengths and, ideally, angles involving carbon and hydrogen atoms due to the difficulties in performing experiments on all the selectively monodeuterated species. For the purpose of exploiting TMA, the SE100 database contains a wide range of hydrocarbon molecules covering aliphatic, cyclic, and aromatic species and involving single, double, and triple carbon–carbon bonds. On the LRA side, the database offers a huge set of entries for density functional parameterization that, in the present work, has been carried out for rev-DSDPBEP86 and PW6B95. As an example, LR plots together with the corresponding 95% confidence intervals are reported in Figure 7 for the CC and CH bond lengths, and they have been obtained on a set of 115 and 162 observables, respectively. The resulting LRA parameters for the rev-DSDPBEP86/jun-cc-pVTZ and PW6B95/jul-cc-pVDZ levels of theory are reported in Table 2 together with mean deviations (MDs), mean absolute deviations (MADs), and maximum negative (Neg.) and positive (Pos.) errors for the bare and LRA-augmented functionals. Concerning rev-DSDPBEP86, the MD and MAD are around 0.0026 Å for both CC and CH bond lengths, while the PW6B95 functional shows an MD of −1.4 × 10^–4^ Å and an MAD of 0.0018 Å for the CC distance, and on average, it systematically overestimates CH bond lengths by 0.006 Å. Upon application of the LRA correction, the MAD for CH and CC distances lowers, respectively, to 5.9 × 10^–4^ and 0.0013 Å for the rev-DSDPBEP86 functional and to 8.5 × 10^–4^ and 0.0017 Å in the case of PW6B95, thus showing an accuracy comparable to that of the most refined composite schemes based on the coupled-cluster theory.

**Table 2:** Statistics and LRA Parameters for Bond Lengths and Valence Angles for rev-DSDPBEP86/jun-cc-pV(T+d)Z and PW6B95/jul-cc-pV(D+d)Z Levels of Theory^a^

parameter	rev-DSDPBEP86	PW6B95	rev-DSDPBEP86-LRA	PW6B95-LRA
CC Bond, N = 115				
MD	0.0026	–0.0001	–0.00002	–0.00006
Neg.	–0.0046	–0.0064	–0.0072	–0.0062
Pos.	0.0081	0.0072	0.0056	0.0073
MAD	0.0028	0.0018	0.0013	0.0018
*A*	–0.00184	0.00014		
*B*	0	0		
CH Bond, N = 162				
MD	0.0026	0.0064	–5 × 10^–6^	–4 × 10^–6^
Neg.	–0.0018		–0.0044	–0.0042
Pos.	0.0054	0.0095	0.0028	0.0030
MAD	0.0026	0.0064	0.00059	0.00084
*A*	–0.00239	–0.00586		
*B*	0	0		
CO Bond, N = 48				
MD	0.0038	–0.00050	0.00004	0.00003
Neg.		–0.0070	–0.0031	–0.0039
Pos.	0.0058	0.0034	0.0022	0.0029
MAD	0.0038	0.0020	0.00070	0.0013
*A*	–0.00297	0.01708		
*B*	0	–0.0212		
CN Bond, N = 39				
MD	0.0031	–0.0017	0.00003	0.00013
Neg.	–0.00027	–0.0082	–0.0034	–0.0056
Pos.	0.0069	0.0039	0.0038	0.0056
MAD	0.0032	0.0026	0.0013	0.0019
*A*	–0.00234	0.01705		
*B*	0	–0.02079		
CS Bond, N = 18				
MD	0.0042	0.00020	2 × 10^–6^	7 × 10^–6^
Neg.		–0.0050	–0.0030	–0.0041
Pos.	0.0088	0.0071	0.0033	0.0060
MAD	0.0041	0.0019	0.0012	0.0017
*A*	–0.01222	–0.01296		–.
*B*	0.01672	0.02188		
CF Bond, *N* = 8				
MD	0.0041	0.0081	1 × 10^–6^	–8 × 10^–6^
Neg.			–0.0012	–0.0017
Pos.	0.0052	0.0093	0.0011	0.0013
MAD	0.0041	0.0081	0.00067	0.00091
*A*	–0.00307	–0.00598		
*B*	0	0		
CCl Bond, *N* = 7				
MD	0.0075	0.0024	–0.00001	–7 × 10^–6^
Neg.			–0.0028	–0.0023
Pos.	0.0091	0.0037	0.0015	0.0012
MAD	0.0075	0.0024	0.0010	0.00075
*A*	–0.0043	–0.0014		
*B*	0	0		
NH Bond, *N* = 14				
MD	0.0022	0.0034	4 × 10^–6^	–8 × 10^–6^
Neg.	–0.0020		–0.0042	–0.0031
Pos.	0.0048	0.0067	0.0026	0.0032
MAD	0.0025	0.0034	0.0011	0.0011
*A*	–0.00216	–0.00331		
*B*	0	0		
OH Bond, *N* = 9				
MD	0.0033	0.0015	3 × 10^–6^	–8 × 10^–6^
Neg.			–0.00094	–0.00098
Pos.	0.0040	0.0025	0.00054	0.00069
MAD	0.0033	0.0015	0.00038	0.00030
*A*	0.24674	0.17529		
*B*	–0.24091	–0.17005		
CCH Angle, *N* = 159				
MD	0.001	0.032	–6 × 10^–5^	–8 × 10^–4^
Neg.	–1.21	–1.21	–1.22	–1.24
Pos.	1.48	1.51	1.48	1.48
MAD	0.16	0.18	0.16	0.18
*A*	–0.00001	–0.00027		
*B*	0	0		
HCH Angle, *N* = 54				
MD	–0.15	–0.33	4 × 10^–4^	–2 × 10^–4^
Neg.	–0.58	–0.90	–0.39	–0.64
Pos.	0.33	0.35	0.37	0.70
MAD	0.17	0.38	0.09	0.22
*A*	0.01695	0.02077		
*B*	–1.77589	–2.01637		
OCO Angle, *N* = 7				
MD	0.12	–0.08	–4 × 10^–4^	–9 × 10^–5^
Neg.	–0.03	–0.30	–0.05	–0.22
Pos.	0.25	0.11	0.08	0.19
MAD	0.13	0.13	0.03	0.10
*A*	–0.06709	0.00063		
*B*	8.1676	0		
HCN Angle, *N* = 31				
MD	0.005	0.17	–9 × 10^–4^	–2 × 10^–4^
Neg.	–0.62	–0.39	–0.63	–0.57
Pos.	0.33	0.59	0.33	0.42
MAD	0.16	0.22	0.16	0.15
*A*	–0.00003	–0.0014		
*B*	0	0		
COH Angle, *N* = 8				
MD	–0.011	0.31		–4 × 10^–4^
Neg.	–0.20	–0.08		–0.15
Pos.	0.12	0.66		0.23
MAD	0.09	0.34		0.09
*A*	0	–0.16466		
*B*	0	17.43968		

a Bond lengths in Å, angles in °; *N*: number of points in the linear fit; MD: mean deviation; Neg.: largest negative error; Pos.: largest positive error; MAD: mean absolute deviation; for angles, only the most important parameterizations are reported; *B* (or *A*) = 0 means that the parameter has been fixed to zero; for the full list, see Table S2 of Supporting Information.

In the case of the CCC and CCH valence angles, the predictions of both the rev-DSDPBEP86 and PW6B95 functionals are in excellent agreement with the SE counterparts, as can be seen in Tables 2 and S2 of Supporting Information, with MAD equal to 0.12°, that is, comparable to the accuracy of the CCSD(T)-based composite schemes, for the former and 0.25° for the latter. With the accuracy limit almost reached, the MAD improvement issuing from the LR correction is negligible, even though it leads to vanishing MDs, thus reducing systematic errors. In the case of the HCH angle, the MD and MAD (over 54 data points) of the rev-DSDPBEP86 predictions from the SE equilibrium values amount to 0.14 and 0.17° and, as in the case of CCC and CCH angles, LRA marginally reduces the MAD but drops the MD to 0. Conversely, the PW6B95 functional shows an MD and a MAD of −0.33 and 0.35°, respectively, which reduce to −1.6 × 10^–4^ and 0.22° after the LRA correction.

### 3.3 Adding Oxygen and Nitrogen Atoms

The LRA parameters for CO and CN bond lengths have been obtained from 48 and 39 data points, respectively, and they are reported in Table 2 while LR plots are shown in Figure S1 of Supporting Information. Concerning the CO moiety, the rev-DSDPBEP86 functional systematically overestimates bond lengths by about 0.004 Å, while the bare PW6B95 density functional performs very well with an MD of −5.0 × 10^–4^ Å and an MAD of only 0.0020 Å. Therefore, for this functional, the LRA correction provides a modest improvement (the MAD lowers to 0.0013 Å), whereas it significantly enhances the performance of the double-hybrid functional, yielding an MD and an MAD, with respect to SE values, of 3.5 × 10^–5^ and 7.0 × 10^–4^ Å, respectively. Before applying LRA, the double-hybrid functional reproduces CN equilibrium distances with an MAD of 0.0032 Å, which reduces to 0.0013 Å when the correction is introduced. The accuracy of the PW6B95 functional in describing CN bond lengths is remarkable, with an MAD of only 0.0026 Å, which is, anyway, somewhat improved by LRA (MAD = 0.0019 Å). Furthermore, systematic errors are strongly reduced, with the MD going from −0.0017 Å for the bare functional to 1.3 × 10^–4^ Å for the corrected one. As in the case of the CCC angle, both the considered functionals yield very good predictions of CCX, CXC, and HCX with X being either oxygen or nitrogen (see Table 2 and Supporting Information, Table S2). Indeed, at the rev-DSDPBEP86 level of theory, all these angles are computed with an MAD lower than 0.16° and a maximum error of 0.4°. The hybrid PW6B95 functional also provides reliable predictions: for CCO, HCO, CCN, and HCN angles, the MADs are in the range between 0.12 and 0.22° and the LRA parameterization does not deliver significant improvements. Conversely, for the COC and CNC angles, the correction lowers the MAD from 0.35 to 0.14° and from 0.23 to 0.07°, respectively, thus reconciling the accuracy with that obtained for the remaining angles involving nitrogen or oxygen atoms in the heavy atom molecular backbone.

### 3.4 Boron-Containing Compounds

The boron element was not included in the previous release of the database; hence, the SE equilibrium structures of BH_2_OH, BH(OH)_2_, BHFOH, and BH_3_NH_3_ have been added in this work. The rev-DSDPBEP86 functional reproduces the equilibrium geometries of these molecules with an MD of 0.005 Å and 0.05° for bond lengths and valence angles, respectively, and a maximum error of 0.009 Å for the B–N distance and of 0.6° for the HBO angle of BH_2_OH. Concerning the PW6B95 hybrid functional, SE equilibrium structures are reproduced with an MD of 0.0003 Å and 0.14°, which increases to 0.005 Å and 0.3°, respectively, if MADs are considered. The maximum errors, reported for B–N and B–F bond lengths, are around 0.01 Å and of 0.9° for the HBO angle of the BHFOH molecule, respectively.

### 3.5 Sulfur-Containing Compounds and Inclusion of the S–S Bridge

As pointed out in Section 3.1, the structural database has been enriched by adding seven molecules containing the sulfur atom, thus making it possible to systematically investigate the prediction of CS bond lengths as well as of SCH, CSC, and CCS bond angles. Linear fits for the CS distance and CSC angle, carried out on a total of 18 and 7 data points, respectively, are shown in Figure S2 of Supporting Information, and the corresponding LRA parameters are reported in Tables 2 and S2 of Supporting Information. In passing, it should be noted that the previous LRA parameterization for the B3LYP and B2PLYP functionals was performed by using the SNSD and cc-pVTZ basis sets, respectively, whereas recent investigations have pointed out the necessity of augmenting the basis set with an additional set of d functions for the S atom (and in general for second-row elements) in order to improve the predictions of structural, spectroscopic,^22^ and thermochemical properties.^8,76,77^ The CS distance is systematically overestimated at the rev-DSDBPEP86/jun-cc-pV(D+d)Z level of theory by about 0.004 Å, with a maximum error of 0.009 Å, whereas CCS, CSC, and SCH angles are predicted with a remarkable accuracy, with the MADs being 0.09, 0.08, and 0.16°, respectively; thus, they require only minor adjustments. The LRA parameterization for the CS bond length reduces the MAD to 0.0012 Å, and it narrows the errors from the corresponding SE equilibrium values to the range of −0.003 to 0.003 Å. Computations carried out at the PW6B95/jul-cc-pV(D+d)Z level predict the CS distance and the SCH and CCS angles with MADs of only 0.0018 Å, 0.3 and 0.1°, respectively. Given the already small deviations, the LRA correction delivers only marginal improvements, even though less systematic errors can be noted as demonstrated by MDs equal to 0. For the CSC angle, LRA, even though performed on a set of only six points, improves the MAD from 0.3° for the bare functional to 0.2° for the LRA-augmented one and, most remarkably, the maximum error reduces from 0.8 to 0.3°. A few words need to be spent for the S–S bridge that, given its relevance for biomolecular functionality, has been included in the database through the SE equilibrium structures of hydrogen disulfide, diallyldisulfide, and diphenyldisulfide. Even though based on just three observations, some trends are apparent: first, both the rev-DSDPBEP86 and PW6B95 functionals overestimate the S–S bond length by about 0.01 Å; second, the deviations are very systematic, and the plot of predicted and SE bond lengths already suggests a linear trend.

### 3.6 Phosphorous-Containing Compounds

Like boron, phosphorous was not present in the previous release of the database; hence, in the present work, the SE equilibrium geometries of HPO,^58^ PH_3_,^2^ CH_2_PH,^47^ and CH_3_CP^57^ have been included. The computed P–H distance is too long by about 0.003 Å and 0.01 Å at the rev-DSDPBEP86/jun-cc-pV(T+d)Z and PW6B95/jul-cc-pV(D+d)Z levels of theory, respectively, whereas for the CP bond length, the deviations appear less systematic, even though this behavior is speculated on the basis of only two observations which, moreover, refer to different bonding environments: a CP double bond in CH_2_PH and a triple bond in CH_3_CP. For the former molecule, the double-hybrid functional overestimates the CP distance by 0.002 Å, whereas the hybrid one underestimates it by −0.004 Å; hence, both of them appear fairly accurate. In the case of CH_3_CP, the error increases to 0.007 Å for the rev-DSDPBEP86 functional, but PW6B95 results closer to the SE value with an error of only −0.002 Å. Concerning bond distances, the largest error is observed for the PO bond of the HPO molecule, with deviations around 0.01 Å for both the functionals. Conversely, all the bond angles involving the phosphorous atom are predicted with good accuracy at the rev-DSDPBEP86 level of theory, with errors within 0.3°, whereas the maximum error amounts to 0.6° in the case of PW6B95. Overall, for these phosphorous-containing molecules, the MADs computed over all the bond lengths and angles are 0.0036 Å and 0.13° at the rev-DSDPBEP86/jun-cc-pV(T+d)Z level and 0.0076 Å and 0.31° at the PW6B95/jul-cc-pV(D+d)Z level of theory, respectively, even though the accuracy could be improved by applying the LRA correction to the structural parameters for which the parameterization has been obtained (i.e., C–*H* and CC bonds and CCH angles).

### 3.7 Halogenated Organics

The molecules contained in the SE100 database allow a full assessment of the performances of the two considered functionals in the prediction of C–F and C–Cl bond lengths and a robust determination of the corresponding LRA parameters (Table 2). This has led to the regression lines reported in Supporting Information, Figure S3. The C–F and C–Cl distances are systematically overestimated at both rev-DSDPBEP86/jun-cc-pV(T+d)Z and PW6B95/jul-cc-pV(D+d)Z levels of theory. The former functional shows MADs of 0.004 and 0.008 Å for the C–F and C–Cl bond lengths, respectively; the PW6B95 functional reproduces C–F distances with an MAD of 0.008 Å, whereas interestingly, it performs very well for the C–Cl bond length (MAD = 0.002 Å). The LRA correction significantly improves the theoretical predictions: rev-DSDPBEP86-LRA yields C–F distances computed with an MAD = 7 × 10^–4^ Å, and deviations from SE values range between −0.001 and 0.001 Å; for the C–Cl bond length, the MAD lowers to 0.001 Å, and the maximum negative and positive errors are −0.0028 and 0.0015 Å, respectively. Improved accuracy is also attained by augmenting PW6B95 with LRA: the MADs lower to 9 × 10^–4^ and 7 × 10^–4^ Å for C–F and C–Cl bond lengths, respectively, and the errors are in the range of −0.0023 to 0.0012 Å.

### 3.8 Issue of OH and NH Acidic Hydrogens

Up to this point, the performances of the rev-DSDPBEP86 and PW6B95 functionals have been assessed only for hydrogens bonded to carbon atoms. In this section, the attention is devoted to the more challenging case of terminal X–H bonds (X = O and N) as these may be involved in intramolecular hydrogen bonds of different strengths. LRs obtained over the 14 N–H data points are reported in Figure S4 of Supporting Information a and b for the rev-DSDPBEP86 and PW6B95 functionals, respectively, and both of them show a very regular trend highlighted by an *R*^2^ value better than 0.9999, vanishing intercepts and slopes very close to 1. Indeed, already the bare functionals provide good predictions for the N–H bond lengths, with the MAD being around 0.0034 and 0.0025 Å for the hybrid and double-hybrid functionals, respectively. Use of LRA improves the accuracy, lowering the MAD to 0.0011 Å and yielding errors in the range between −0.0042 and 0.0026 Å for rev-DSDPBEP86 and between −0.0031 and 0.0032 Å for PW6B95. The CNH angles are also well reproduced on average, with the MADs being around 0.25°, even though the maximum deviation can reach 1.5 and 0.8° at rev-DSDPBEP86/jun-cc-pVTZ and PW6B95/jul-cc-pVDZ levels of theory, respectively. In the present case, however, LRA provides only negligible improvements, probably because the residuals between theoretical and SE data do not follow a regular and systematic trend. By considering the obtained results, no particular concerns can be reported for the N–H bond length and the CNH angle, but it should be noted that glycine is the only molecule of the SE100 database featuring an intramolecular hydrogen bond involving the NH group.The O–H bond lengths are overestimated, on average, by 0.0041 and 0.0026 Å at the rev-DSDPBEP86 and PW6B95 levels of theory, respectively, with maximum errors around 0.009 Å. Linear fits carried out by considering all the 14 entries have led to unsatisfactory results, but closer inspection of the residuals of theoretical values from the corresponding SE equilibrium counterparts (see Figure S5 of Supporting Information for the rev-DSDPBEP86 functional) has revealed that those associated with free O–H bonds tend to cluster between 0.003 and 0.004 Å, whereas OH groups involved in hydrogen bonds show much scattered residuals. This has suggested to parameterize LRA only for free OH bonds, leading to the linear fits reported in Supporting Information, Figures S4c,d. Upon applying the LRA correction, the performance of both functionals is comparable, with an MAD around 3 × 10^–4^ Å and maximum negative and positive deviations around −9 and 6 × 10^–4^ Å, respectively. The COH angles, on the other hand, appear well described at the rev-DSDPBEP86 level irrespective of whether they are involved or not in intermolecular hydrogen bonds: SE equilibrium values are reproduced with an MAD of 0.09° and errors in the range of −0.21 to 0.12°. The PW6B95 functional shows larger deviations (MAD = 0.34°) that are smoothed out when employing LRA, which attains the same MAD as the rev-DSDPBEP86 functional and errors between −0.15 and 0.23°.

### 3.9 Playing with Nano-LEGO

The SE100 database represents the cornerstone for the building of the nano-LEGO platform, which, starting from the collection of a large panel of SE equilibrium geometries, allows the determination of accurate structures through the exploitation of LRA and TMA, with the twofold aim of providing (i) reliable guess geometries for the SE fitting procedure, thus overcoming the limitations connected with the lack of isotopic substitutions, and (ii) a reliable and cost-effective quantum chemical approach for obtaining accurate geometries for medium to large molecular systems, thus overcoming the limitations connected to the computational cost of coupled-cluster-based approaches. On these grounds, the following subsections present test cases for the validation of the nano-LEGO approach related to these two topics.

**Figure 8:**
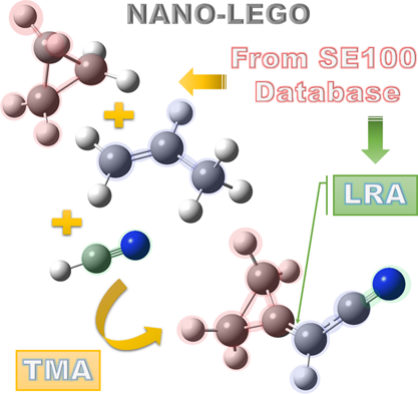
Schematic diagram of Nano-LEGO: starting from the SE100 database, TMA for (cyanomethylene)cyclopropane is carried out by using cyclopropane, propene, and hydrogen cyanide, while LRA is used for interfragment structural parameters.

A schematic representation of the nano-LEGO framework is provided by Figure 8. The example considered in this figure is (cyanomethylene)cyclopropane, an isomer of pyridine and a prebiotic molecule of astrochemical relevance, whose millimeter-wave spectra have been recently analyzed, obtaining the rotational constants for the main isotopic species.^78^ In this molecule, three fragments can be envisaged: the C_3_H_4_, C_2_H, and CN moieties. DFT geometry optimizations have been carried out for (cyanomethylene)cyclopropane as well as for the template molecules, namely, cyclopropane, propylene, and HCN. The data available in the SE100 database have then been used to correct the structural parameters of the three moieties in which the whole molecule has been dissected. Finally, the DFT bond lengths and (possibly) valence angles connecting the three “nano-LEGO bricks” have been corrected using LRA.

To test the accuracy of the equilibrium structures derived using the nano-LEGO platform, one can compare the corresponding rotational constants with those issuing from the SE approach. The results listed in Table 3 are indeed impressive: ground-state rotational constants are reproduced, on average, within 0.02 and 0.06% by the rev-DSDPBEP86-LRA and PW6B95-TMA methods, respectively, with maximum errors of 0.03% and 0.1%. These findings should be compared to an estimated accuracy of 0.1% delivered by state-of-the-art composite schemes based on coupled-cluster theory,^79,80^ thus showing that the nano-LEGO approach can be profitably used to predict accurate equilibrium geometries, with an accuracy comparable to that of the most-refined coupled-cluster methods but with a reduction of the computational cost of 2–4 orders of magnitude depending on the employed functional.

**Table 3:** Equilibrium Geometries and Ground-State Rotational Constants of (Cyanomethylene)cyclopropane at rev-DSDPBEP86/jun-cc-pVTZ and PW6B95/jul-cc-pVDZ Levels of Theory and upon LRA and TMA Correction and Comparison to the Experimental Rotational Constants^a^

	rev-DSDPBEP86	PW6B95	revDSDPBEP86-LRA	PW6B95-LRA	rev-DSDPBEP86-TMA	PW6B95-TMA
C1N2	1.1633	1.1592	1.1605	1.1582	1.1591	1.1598
C1C3	1.4324	1.4265	1.4296	1.7272	1.4297	1.4300
C3C4	1.3273	1.3276	1.3252	1.3267	1.3255	1.3278
C4C5	1.4626	1.4580	1.4596	1.4591	1.4589	1.4589
C5C6	1.5388	1.5320	1.5354	1.5343	1.5351	1.5329
C3H7	1.0833	1.0872	1.0809	1.0809	1.0806	1.0801
C5H8	1.0837	1.0875	1.0811	1.0811	1.0810	1.0807
C5H9	1.0836	1.0874	1.0811	1.0811	1.0809	1.0806
C1C3C4	121.41	121.81	121.38	121.69	121.14	121.12
C3C4C5	148.31	148.16	148.28	148.01	148.24	148.01
C4C5C6	58.19	58.23	58.18	58.17	58.18	58.17
C3C4H7	121.49	121.32	121.49	121.29	121.77	121.75
C4C5H8	118.22	118.38	118.22	118.35	118.17	118.15
C5C6H10	117.76	118.10	117.76	118.07	117.71	117.87
C3C4C5H8	73.31	71.97	n.a.	n.a.	n.a.	n.a.
C4C5C6H10	107.48	107.52	n.a.	n.a.	n.a.	n.a.
*A*	12657.507	12778.564	12647.603	12699.661	12620.703	12628.917
*B*	2024.628	2024.199	2038.517	2033.747	2043.863	2039.953
*C*	1786.367	1788.412	1796.792	1793.615	1800.438	1797.156
MAD %^b^	0.47	0.75	0.02	0.29	0.21	0.06

a Bond lengths in Å and angles in °. Rotational constants in MHz.

b Percentage mean absolute deviation from experimental data:^78^*A* = 12644.003 MHz, *B* = 2038.862 MHz, *C* = 1797.043 MHz. Theoretical equilibrium rotational constants augmented by vibrational contributions evaluated at the PW6B95/jul-cc-pV(D+d)Z level: Δ*A*^vib^ = −80.314 MHz, Δ*B*^vib^ = −3.747 MHz, Δ*C*^vib^ = −5.216 MHz.

#### 3.9.1 Fishing in the Sea of Geometry Bricks

**Figure 9:**
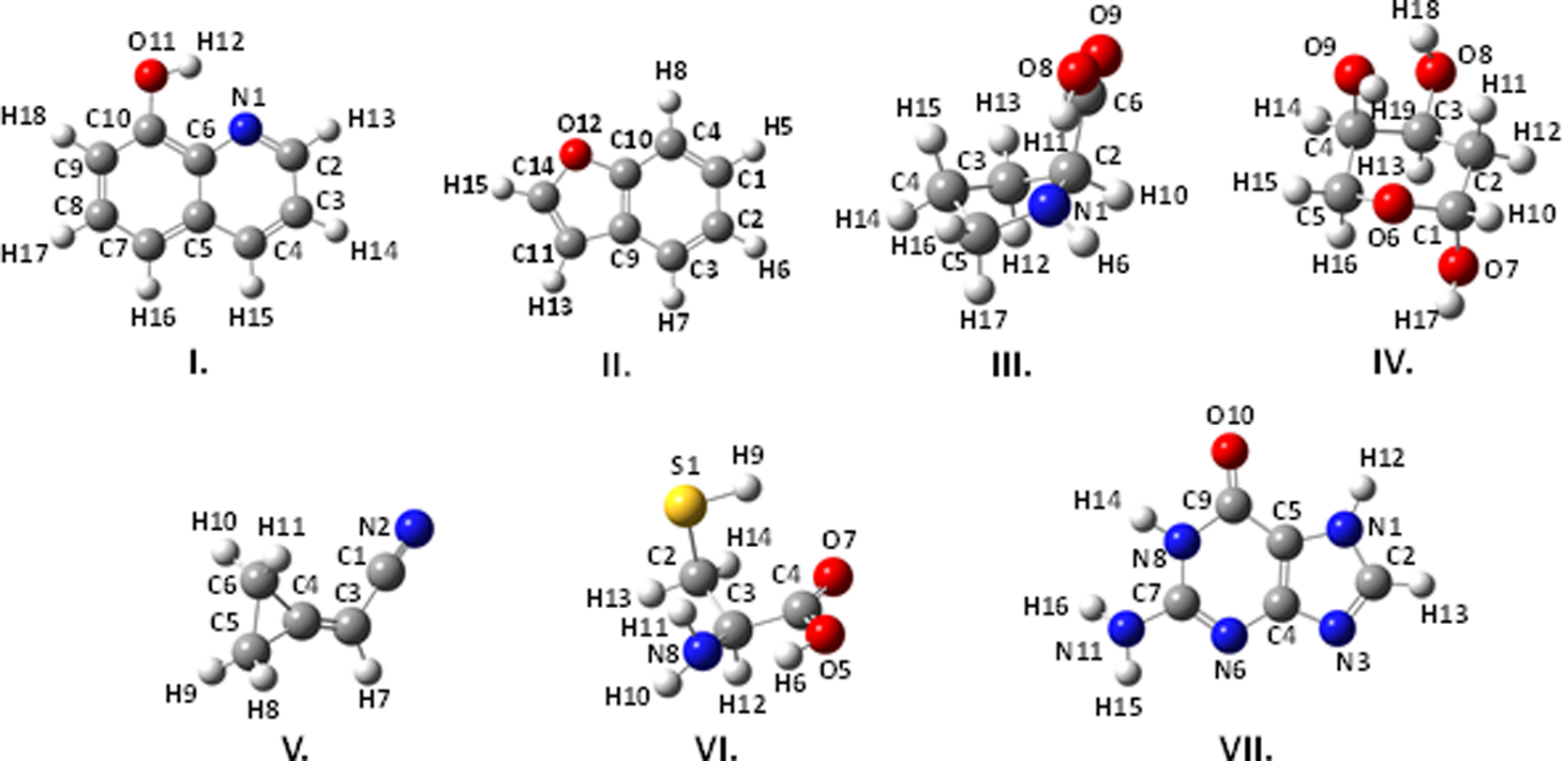
Structure and atom labeling of (I) 8-hydroxyquinoline, (II) benzofuran, (III) proline, (IV) 2-deoxyribose, (V) (cyanomethylene)cyclopropane, (VI) cysteine, and (VII) guanine.

In this subsection, the nano-LEGO procedure is employed to generate reliable guess geometries and provide very accurate values for the geometrical parameters related to missing isotopic substitutions, the lack of deuteration of carbon atoms being one of the most common situations. For the purpose, the determination of the equilibrium geometry of 8-hydroxyquinoline (8-HQ) and benzofuran, whose structures and atom labeling are detailed in Figure 9, has been considered. For the former, a SE equilibrium geometry has been recently obtained based on the measurement of the rotational constants for 13 isotopologues (namely, all singly ^13^C isotopic substitutions, the ^15^N and ^18^O ones, and the deuteration of the OH group).^81^ However, due to the lack of C–D-containing isotopologues, the mass-dependent structural evaluation model has been adopted and a number of constraints have been imposed in the fit, with C–H distances and CCH angles fixed to B2PLYP-D3/aug-cc-pVTZ values.

Following the nano-LEGO procedure, the theoretical equilibrium structure of 8-HQ has been obtained by exploiting the TMA using phenol and pyridine as templates. This has been used as a guess geometry in the SE least-squares fitting procedure based on the experimental rotational constants as well as the B3LYP-D3 vibrational and electronic corrections reported in ref (81). During the fits, all the structural parameters have been relaxed, with the exception of CH and HCC bond lengths that have been kept fixed at the TMA theoretical values. In addition, as the presence of condensed cycles may impose some strains on the geometry, resulting in a loss of accuracy for the structural parameters describing the neighborhoods of the contact points of the rings, the theoretical values of the C9–C10 distance and the C4C10C9, C5C10C9, C3C4C10, and C6C5C10 angles have been used as predicate observations. The resulting SE equilibrium geometry, reported in Table 4, is in fair agreement with the mass-dependent *r*_m_^(2)^ geometry of McNaughton et al.,^81^ with the difference that in the present case, 21 structural parameters have been directly refined instead of 6.

**Table 4:** Equilibrium Structure of 8-Hydroxyquinoline^a^

	SE^b^	SE^c^	rev-DSD^b^	rev-DSD + NanoLego^b^
C2N	1.3151(13)	1.3194(14)	1.3179	1.3156
C2C3	1.4134(14)	1.4134(13)	1.4139	1.4117
C3C4	1.3697(13)	1.3754(10)	1.3738	1.3711
C4C10	1.4139(17)	1.4094(20)	1.4155	1.4128
C9C10	1.4159(14)	1.4224(12)	1.4180	1.4158
C5C10	1.4150(18)	1.4183(19)	1.4171	1.4153
C5C6	1.3713(16)	1.3774(15)	1.3756	1.3720
C6C7	1.4110(14)	1.4124(17)	1.4136	1.4138
C7C8	1.3712(14)	1.3774(12)	1.3758	1.3722
C8O	1.34717(78)	1.3461(15)	1.3498	1.3463
OH	0.9658(13)	0.9751(40)	0.9736	0.9703
C3H14^fixed^	1.0775	1.1000	1.0828	1.0775
C4H15^fixed^	1.0818	1.1003	1.0847	1.0818
C5H16^fixed^	1.0807	1.1003	1.0838	1.0807
C6H17^fixed^	1.0810	1.0999	1.0837	1.0810
C7H18^fixed^	1.0788	1.0999	1.0827	1.0788
NC2C3	123.414(42)	123.30(10)	123.418	123.378
C2C3C4	119.130(28)	119.200(70)	119.147	119.157
C3C4C10	119.541(56)	119.500(80)	119.520	119.500
C4C10C9	116.52(16)	116.64(12)	116.54	116.55
C5C10C9	119.43(16)	119.16(15)	119.35	119.35
C6C5C10	119.436(62)	119.49(13)	119.452	119.481
C5C6C7	121.707(28)	121.80(13)	121.737	121.751
C6C7C8	119.783(50)	119.80(10)	119.777	119.607
C9C8O	118.33(10)	118.690(70)	118.555	118.539
C8OH	105.545(51)	105.50(18)	105.326	105.299
H13C2C3^fixed^	119.969	119.00	120.019	119.969
H14C3C4^fixed^	121.315	120.69	121.255	121.315
H15C4C5^fixed^	119.502	118.611	119.502	119.502
H16C5C6^fixed^	121.009	120.86	121.006	121.09
H17C6C7^fixed^	118.629	118.99	118.605	118.629
H18CC8^fixed^	119.418	120.65	119.394	119.418

a Bond lengths in Å and angles in °. Figures in parentheses are standard deviations in the units of the last significant digits.

b This work.

c From ref (81).

A similar procedure has been employed for determining the SE equilibrium geometry of benzofuran: indeed, TMA has been employed to describe the parameters related to H13C14H15O12 and H7C3C2H6C1CHC4H8 portions of the molecule, templated on the basis of furan and benzene, respectively, whereas the rev-DSDPBEP86-LRA method has been employed for determining the bond lengths and angles involved in the linkage between the two condensed rings. The fitting procedure has been carried out by using the SE rotational constants obtained by correcting those measured for the ground state of the parent and all the ^13^C and ^18^O singly substituted isotopic species^82^ through PW6B95/jul-cc-pVDZ vibrational contributions. Due to the lack of isotopic substitutions for the hydrogen atoms, the CH bond length and the HCC and HCO bond angles have been constrained to the rev-DSDPBEP86-LRA values. The fit has converged, with a standard weighted root-mean-square deviation of 2.3 × 10^–3^ umaÅ^2^, to the equilibrium structure reported in Table 5. As can be seen, all the fitted parameters appear well determined with errors within 9 × 10^–3^ Å for bond lengths and 0.04° for bond angles.

**Table 5:** Semi-Experimental and Theoretical Equilibrium Structure of Benzofuran^a^

	SE	rev-DSDPBEP86	PW6B95	rev-DSDPBEP86-LRA	PW6B95-LRA	rev-DSDPBEP86-TMA
C1C2	1.40454(50)	1.4064	1.4035	1.4038	1.4037	1.4041
C2C3	1.38583(33)	1.3885	1.3862	1.3860	1.3864	1.3862
C1C4	1.38733(34)	1.3907	1.3887	1.3881	1.3889	1.3884
C3C9	1.40001(89)	1.4011	1.3989	1.3985	1.3991	1.3985
C4C10	1.38440(80)	1.3882	1.3853	1.3857	1.3855	1.3857
C9C11	1.44013(89)	1.4433	1.4388	1.4406	1.4390	1.4406
C10O12	1.36518(83)	1.3674	1.3624	1.3634	1.3644	1.3648
C14C11	1.35117(51)	1.3547	1.3524	1.3522	1.3526	1.3510
C1H5^fixed^	1.0807	1.0833	1.0865	1.0807	1.0801	1.0795
C2H6^fixed^	1.0808	1.0833	1.0865	1.0808	1.0801	1.0795
C3H7^fixed^	1.0809	1.0835	1.0865	1.0809	1.0801	1.0797
C4H8^fixed^	1.0797	1.0823	1.0852	1.0797	1.0789	1.0785
C11H13^fixed^	1.0755	1.0781	1.0812	1.0755	1.0749	1.0756
C14H15^fixed^	1.0747	1.0773	1.0806	1.0747	1.0742	1.0740
C1C2C3	121.279(18)	121.34	121.33	121.32	121.191	121.316
C1C2C4	121.399(19)	121.36	121.41	121.33	121.27	121.33
C2C3C9	118.272(29)	118.31	118.33	118.28	118.20	118.28
C1C4C10	116.267(32)	116.29	116.26	116.26	116.13	121.26
C3C9C11	135.561(30)	135.71	135.74	135.68	135.59	135.68
C4C10O12	125.743(35)	125.75	125.93	125.66	125.87	125.66
C9C11C14	105.753(28)	105.88	105.85	105.85	105.73	105.85
H5C1C2^fixed^	119.32	119.32	119.34	119.32	119.30	119.32
H6C2C1^fixed^	119.09	119.09	119.11	119.09	119.07	119.09
H7C3C2^fixed^	120.77	120.77	120.79	120.77	120.76	120.77
H8C4C1^fixed^	122.19	122.19	122.16	122.19	122.13	122.19
H13C11C9^fixed^	128.04	128.04	128.04	128.04	128.01	127.98
H15C10O12^fixed^	115.29	115.17	115.29	115.19	115.24	115.20

a Bond lengths in Å and angles in °. Figures in parentheses are standard deviations in the units of the last significant digits.

#### 3.9.2 LRA and TMA Route toward Accurate Equilibrium Geometries of Biomolecules

The nano-LEGO procedure can be used to build accurate geometries for medium-size biochemical molecules at the computational cost of the underlying DFT computations. For the purpose, the SE equilibrium geometries determined by Demaison and collaborators for proline^83^ and 2-deoxyribose^84^ have been employed for showing that by using either LRA or a combination of TMA and LRA (TMA + LRA), rev-DSDPBEP86 and PW6B95 equilibrium geometries can be improved to such an extent that they fully reconcile with the SE counterparts. As a test of consistency, for proline, TMA has been carried out according to two different methods: in the first one, pyrrole and glyoxylic acid have been used as template molecules; in the second one, pyrrole and glycine have been employed. Both of them have led to (nearly) the same results when applied in combination with rev-DSDPBEP86 and PW6B95 equilibrium geometries, and hence, only those obtained from the second template model are reported. Concerning proline (see Table S3 of Supporting Information), bond lengths are systematically overestimated at the rev-DSDPBEP86/jun-cc-pVTZ level of theory by 0.003 Å and a similar result is given by the PW6B95/jul-cc-pVDZ model chemistry. Even though these results can already be considered very good, the structural predictions improve upon augmentation through LRA. Indeed, at the rev-DSDPBEP86-LRA level, the MAD decreases to 0.001 Å and all errors fall in the −0.002 to 0.003 Å range; the same MAD is obtained at the PW6B95-LRA level with errors within −0.002 and 0.002 Å, thus reaching the same accuracy of CCSD(T)-based geometrical composite approaches like the cheap scheme.^3,79^ The combination of TMA and LRA also delivers the same accuracy, with MDs close to 0 and MADs of 0.001 for both the functionals and errors in the range of −0.003 to 0.002 and −0.003 to 0.001 Å for the rev-DSDPBEP86-TMA + LRA and PW6B95-TMA + LRA models, respectively. Concerning valence angles, excellent predictions are delivered already by the bare functionals to the extent that LRA or TMA corrections provide only a negligible improvement, as expected from the results previously obtained for the LRA parameterization.

In the case of 2-deoxyribose, only LRA has been adopted due to the lack of suitable template molecules in the SE100 database. Some tests have been performed trying to template this molecule using glycolaldehyde, oxirane, and propanal, but they have not led to completely satisfactory results in terms of maximum deviations from the SE geometry due to the different chemical environment experienced by the atoms of the target and the template molecules. Strictly speaking, this does not represent a real issue for the nano-LEGO approach given the high accuracy of corrections based on LRA. Indeed, for bond lengths, the MAD lowers from about 0.003 Å for the bare functionals to 0.001 Å upon application of the LR correction (see Supporting Information, Table S5) and MDs around 0.0005 and −0.0002 Å are respectively scored by rev-DSDPBEP86-LRA and PW6B95-LRA, thus confirming the reliability of the method. For 2-deoxyrobose as well, the rev-DSDPBEP86/jun-cc-pVTZ and PW6B95/jul-cc-pVDZ levels of theory confirm their reliability in the prediction of bond angles and LRA augmentation has little effect on their values.

An additional issue to be addressed at this point concerns dihedral angles, for which use of LRA has not been possible. Overall, for the two molecules considered, both rev-DSDPBEP86 and PW6B95 yield quite reliable predictions with MADs around 0.2 and 0.4°, respectively, in the case of 2-deoxyribose and 0.5 and 1.2° for proline. However, it should be pointed out that for this molecule, the statistics can be in part biased by the conformational complexity of the molecule, which also features an intramolecular hydrogen bond, and by the fact that in the SE geometry, the torsional angles defining the position of the hydrogen atoms have been mainly determined on the basis of the adopted predicates.^83^

Finally, the nano-LEGO tool has also been applied for the prediction of the ground-state rotational constants of medium-size molecules of biochemical relevance. The procedure is here illustrated and validated focusing on cysteine and guanine (see Figure 9), whose rotational spectra have been investigated by laser ablation supersonic jet Fourier transform microwave spectroscopy some years ago, leading to the determination of the ground-state rotational constants for different isomers.^85,86^ For the purpose of the present work, only the most stable ones are considered, namely, cysteine IIb and the keto N7H form of guanine. For guanine, both LRA and the integrated TMA + LRA have been used to correct the equilibrium geometries obtained at rev-DSDPBEP86/jun-cc-pV(T+d)Z and PW6B95/jul-cc-pv(D+d)Z levels of theory, while for cysteine, only LRA has been used. The equilibrium geometries calculated for the bare functionals as well as those obtained upon introducing the nano-LEGO correction are reported in Tables 6 and 7 for cysteine and guanine, respectively, together with the corresponding ground-state rotational constants and their comparison to the values measured experimentally. For the application of TMA to guanine, uracil and imidazole have been employed. From these equilibrium geometries, the corresponding equilibrium rotational constants have been straightforwardly derived and augmented by vibrational corrections computed at the PW6B95/jul-cc-pV(D+d)Z level, thus obtaining ground-state rotational constants that can be directly compared with the values measured experimentally. It is interesting to note that already the PW6B95 and rev-DSDPBEP86 functionals provide good values of ground-state rotational constants, with percentage mean absolute deviations (MAD %) within 0.8%, even though errors as large as 0.9–1% can be observed. In passing, it should be pointed out that the PW6B95 functional performs better than the widely used B3LYP functional, and hence, even without corrections, its use must be encouraged in order to predict structural parameters and to assist rotational spectroscopy investigations. On the other hand, as already pointed out,^22^ the rev-DSDPBEP86 model represents a valid alternative to B2PLYP. Upon playing nano-LEGO to work out improved equilibrium structures, the overall MAD decreases, and most importantly, a more consistent description of the rotational constants is obtained, without any outlayer and a maximum deviation of 0.6% observed for the *B*_0_ rotational constant of cysteine. However, it should be noted that for this molecule, the main cause for the observed deviation is related to a potential bias in the vibrational corrections because of the presence of large amplitude motions (LAMs), mainly corresponding to the torsional degrees of freedom of the molecule (very notably the rotation of the S–H) group. When LAMs are present, the cubic (and quartic as well) force constants can be ill determined and inflated by nonphysical contributions due to the unreliability of the fourth-order Taylor series expansion of the potential energy. In the present context, the problem has been mitigated by resorting to a reduced-dimensionality GVPT2 calculation, in which the normal modes corresponding to LAMs have been decoupled from the remaining small amplitude motions, thus preventing the presence of unusually large force constants.^87,88^

**Table 6:** Equilibrium Geometries and Ground-State Rotational Constants of Cysteine at Rev-DSDPBEP86/jun-cc-pVTZ and PW6B95/jul-cc-pVDZ Levels of Theory and upon LRA Correction and Comparison to the Experimental Rotational Constants^a^

	rev-DSDPBEP86	PW6B95	rev-DSDPBEP86-LRA	PW6B95-LRA
S1C2	1.8201	1.8162	1.8146	1.8145
C2C3	1.5289	1.5254	1.5261	1.5257
C3C4	1.5375	1.5325	1.5347	1.5327
C4O5	1.3361	1.3307	1.3322	1.3322
O5H6	0.9808	0.9801	0.9819	0.9818
C4O7	1.2063	1.2047	1.2027	1.2041
C3N8	1.4625	1.4567	1.4591	1.4608
S1H9	1.3404	1.3461	1.3404^b^	1.3461^b^
N8H10	1.0171	1.0173	1.0149	1.0139
N8H11	1.0118	1.0114	1.0096	1.0081
C3H12	1.0959	1.0984	1.0933	1.0919
C2H13	1.0916	1.0938	1.0889	1.0874
C2H14	1.0883	1.0914	1.0857	1.0850
S1C2C3	113.00	112.94	113.0	112.94
C2C3C4	110.24	110.21	110.22	110.08
C3C4O5	114.01	113.86	113.93	113.80
C4O5H6	104.91	104.96	104.91	105.11
C3C4O7	122.90	122.96	122.82	122.90
C4C3N8	109.44	109.16	109.44	109.16
C2S1H9	95.96	95.65	95.96^b^	95.65^b^
C3N8H10	109.44	109.35	109.23	109.36
C3N8H11	111.45	111.87	111.23	111.88
C4C3H12	104.97	104.77	104.97	104.75
S1C2H13	105.54	105.87	105.44	105.76
S1C2H14	109.37	109.52	109.27	109.40
S1C2C3C4	–67.80	–68.27	n.a.	n.a.
C2C3C4O5	146.94	148.82	n.a.	n.a.
C3C4O5H6	–4.84	–5.77	n.a.	n.a.
O5C4C3O7	–178.48	–178.70	n.a.	n.a.
C2C4C3N8	–128.37	–128.69	n.a.	n.a.
C3C2S1H9	71.73	71.20	n.a.	n.a.
C4C3N8H10	88.47	88.82	n.a.	n.a.
H10C3N8H11	119.38	119.50	n.a.	n.a.
N8C4C3H12	–115.45	–115.27	n.a.	n.a.
C3S1C2H13	119.95	120.33	n.a.	n.a.
H13S1C2H14	116.80	116.88	n.a.	n.a.
*A*	3044.272	3081.357	3062.788	3080.929
*B*	1599.210	1606.108	1616.886	1615.949
*C*	1322.812	1325.263	1333.082	1328.950
MAD %^c^	0.66	0.28	0.34	0.37

a Bond lengths in Å and angles in °. Rotational constants in MHz.

b Not parameterized, uncorrected value.

c Percentage mean absolute deviation from experimental data:^85^*A* = 3071.437 MHz, *B* = 1606.5366 MHz, *C* = 1331.8019 MHz. Theoretical equilibrium rotational constants augmented by vibrational contributions evaluated at the PW6B95/jul-cc-pV(D+d)Z level: Δ*A*^vib^ = −29.267 MHz, Δ*B*^vib^ = −11.122 MHz, Δ*C*^vib^ = −9.114 MHz.

**Table 7:** Equilibrium Geometries and Ground-State Rotational Constants of Guanine at rev-DSDPBEP86/jun-cc-pVTZ and PW6B95/jul-cc-pVDZ Levels of Theory and upon LRA and TMA Augmentation and Comparison to the Experimental Rotational Constants^a^

	rev-DSDPBEP86	PW6B95	rev-DSDPBEP86-LRA	PW6B95-LRA	rev-DSDPBEP86-TMA	PW6B95-TMA
N1C2	1.3615	1.3563	1.3583	1.3587	1.3598	1.3583
C2N3	1.3205	1.3161	1.3174	1.3174	1.3158	1.3152
N3C4	1.3722	1.3668	1.3690	1.3693	1.3719	1.3750
C4C5	1.3889	1.3896	1.3863	1.3898	1.3809	1.3824
C4N6	1.3688	1.3616	1.3656	1.3640	1.3667	1.3644
C7N6	1.2990	1.2985	1.2960	1.2999	1.2948	1.2965
C7N8	1.3775	1.3732	1.3743	1.3759	1.3739	1.3746
N8C9	1.4089	1.4062	1.4056	1.4094	1.4038	1.4037
C9O10	1.2230	1.2221	1.2194	1.2217	1.2185	1.2193
C7N11	1.3814	1.3760	1.3781	1.3787	1.3781^b^	1.3787^b^
N1H12	1.0064	1.0066	1.0043	1.0032	1.0029	1.0024
C2H13	1.0789	1.0812	1.0724	1.0749	1.0776	1.0773
N8H14	1.0104	1.0099	1.0082	1.0065	1.0085	1.0083
N11H15	1.0094	1.0090	1.0072	1.0056	1.0072^b^	1.0056^b^
N11H16	1.0087	1.0087	1.0065	1.0053	1.0065^b^	1.0053^b^
N1C2N3	113.30	113.35	113.30^c^	113.35^c^	113.50	113.66
C2N3C4	104.52	104.62	104.50	104.37	104.32	104.28
N3C4C5	110.33	110.25	110.33	110.25	110.33	110.24
C5C4N6	124.37	124.32	124.37	124.32	124.31	124.33
C4N6C7	113.83	114.07	113.83	113.85	113.84	114.03
N6C7N8	124.68	124.43	124.68^c^	124.43^c^	124.80	124.53
C7N8C9	125.45	125.54	125.45	125.37	125.35	124.45
N8C9O10	121.65	121.36	121.65^c^	121.36^c^	121.92	121.56
N6C7N11	120.01	119.87	120.01^c^	119.87^c^	120.01^c^	119.87^c^
C2N1H12	127.76	127.74	127.51	127.76	127.60	127.64
N1C2H13	121.80	121.78	121.79	121.61	121.75	121.72
C7N8H14	119.58	119.80	119.35	119.81	119.59	119.76
C7N11H15	111.60	111.93	111.38	111.95	111.38^b^	111.95^b^
C7N11H16	116.19	116.76	115.97	116.77	115.97^b^	116.77^b^
N1C5C4N6	–179.49	–179.43	n.a.	n.a.	n.a.	n.a.
N3C4N6C7	179.29	179.35	n.a.	n.a.	n.a.	n.a.
C4N6C7N8	0.80	0.74	n.a.	n.a.	n.a.	n.a.
C4N6C7N11	–176.24	–176.37	n.a.	n.a.	n.a.	n.a.
N6C7N8H14	–175.11	–175.89	n.a.	n.a.	n.a.	n.a.
N8C7N11H15	170.60	170.74	n.a.	n.a.	n.a.	n.a.
H15C7N11H16	–131.62	–132.93	n.a.	n.a.	n.a.	n.a.
*A*	1911.495	1918.428	1921.161	1921.310	1922.995	1920.585
*B*	1116.019	1121.746	1121.377	1122.980	1121.366	1119.658
*C*	705.277	708.436	708.703	709.318	708.948	707.893
MAD %^d^	0.53	0.09	0.04	0.07	0.03	0.14

a Bond lengths in Å and angles in °. Rotational constants in MHz.

b From the corresponding LRA.

c Not parameterized, uncorrected value.

d Percentage mean absolute deviation from experimental data:^86^*A* = 1922.155 MHz, *B* = 1121.6840 MHz, *C* = 709.0079 MHz. Theoretical equilibrium rotational constants augmented by vibrational contributions evaluated at the PW6B95/jul-cc-pVDZ level: Δ*A*^vib^ = −11.662 MHz, Δ*B*^vib^ = −6.715 MHz, Δ*C*^vib^ = −4.227 MHz.

On these grounds, the results here reported for cysteine and guanine can be considered the most accurate equilibrium geometries obtained until now for these molecules.

## 4 Conclusions and Outlook

The large set of SE equilibrium geometries obtained in our previous database has been extended to further molecular moieties and to systems also containing B and P atoms. The resulting SE100 database allows an unbiased judgement of the performances of different model chemistries for the prediction of molecular geometries. Next, LR analyses have been performed for the PW6B95 and rev-DSDPBEP86 functionals, which were selected as the best performers among hybrid and double-hybrid functionals, respectively, especially in connection with “calendar” basis sets of double- and triple-zeta quality. The availability of accurate geometries for the most significant building blocks of biomolecules and of accurate LRs for the missing parameters paves the route for the systematic application of our black-box nano-LEGO approach to the refinement of geometries of large molecules of current biological and/or technological interest.

Of course, a number of issues remain to be analyzed to improve its performances. Among the most important, we can mention the following:

1) Inclusion in the database of other building blocks, for instance, ionic species, free radicals, and transition metals. Here, the main issue is related to the availability of accurate reference structures, but the limitations of experimental data can be effectively overcome by state-of-the-art QM approaches at least for not too large building blocks. In this connection, recent work has unequivocally shown that coincident SE and purely theoretical equilibrium structures can be obtained for molecules as large as pyrimidine.^89^
2) Test of cheaper computational models (GGA functionals, smaller basis sets, semi-empirical methods) for the prediction of accurate geometries of very large molecular systems. In this connection, what really matters is not the extent of absolute errors but, rather, their systematic nature, which can be effectively taken into account by the TMA and LRA models.
3) Flexible molecules. For these, VPT2 evaluation of vibrational correction is no longer sufficient and LAMs must be treated by proper variational procedures possibly employing curvilinear coordinates. Effective tools are already available for a reduced number of LAMs, but systematic studies are still lacking. At the same time, the accuracy of current density functionals and the reliability of LRs for dihedral angles are still to be unequivocally proven.

While work along these and related lines is in progress in our laboratory, we think that already the present version of the nano-LEGO tool represents significant progress toward the computation of accurate structures for molecular systems of fundamental and biochemical/technological interest at an affordable computational cost.

## Supporting Information Available

SE, rev-DSDPBEP86/jun-cc-pV(T+d)Z, and PW6B95/jul-cc-pV(D+d)Z equilibrium geometries of the molecules present in the SE100 database; statistics and LRA parameters for valence angles for rev-DSDPBEP86/jun-cc-pV(T+d)Z and PW6B95/jul-cc-pV(D+d)Z levels of theory; theoretical equilibrium geometries of proline at rev-DSDPBEP86/jun-cc-pVTZ and PW6B95/jul-cc-pVDZ levels of theory upon LRA and TMA augmentation and comparison to the SE equilibrium structure; theoretical equilibrium geometries of 2-deoxyribose at rev-DSDPBEP86/jun-cc-pVTZ and PW6B95/jul-cc-pVDZ levels of theory and upon LRA augmentation and comparison to the SE equilibrium structure; LR plots for CO and CN bond lengths and for COC and CNC angles; LR plots for CS bond length and CSC angle; LR plots for CF and CCl bond lengths; LR plots for OH and NH bond lengths and for COH angle; and residuals of OH bond distances from SE equilibrium values (PDF)

## References

[ref1] Equilibrium Molecular Structures: From Spectrocopy to Quantum Chemistry; Demaison J.; Boggs J. E.; Czázár A. G., Eds.; CRC Press.: Boca Raton, 2011.

[ref2] Demaison J. Experimental, semi-experimental and ab initio equilibrium structures. Mol. Phys. 2007, 105, 3109–3138. 10.1080/00268970701765811.

[ref3] Puzzarini C.; Barone V. Diving for accurate structures in the ocean of molecular systems with the help of spectroscopy and quantum chemistry. Acc. Chem. Res. 2018, 51, 548–556. 10.1021/acs.accounts.7b00603. 29400950

[ref4] Grimme S.; Steinmetz M. Effects of London dispersion correction in density functional theory on the structures of organic molecules in the gas phase. Phys. Chem. Chem. Phys. 2013, 15, 16031–16042. 10.1039/c3cp52293h. 23963317

[ref5] Brémond É.; Savarese M.; Su N. Q.; Pérez-Jiménez A. J.; Xu X.; Sancho-García J. C.; Adamo C. Benchmarking Density Functionals on Structural Parameters of Small-/Medium-Sized Organic Molecules. J. Chem. Theory Comput. 2017, 12, 459–465. 10.1021/acs.jctc.5b01144. 26730741

[ref6] Grimme S.; Bannwarth C.; Shushkov P. A Robust and Accurate Tight-Binding Quantum Chemical Method for Structures, Vibrational Frequencies, and Noncovalent Interactions of Large Molecular Systems Parametrized for All spd-Block Elements (Z = 1-86). J. Chem. Theory Comput. 2017, 13, 1989–2009. 10.1021/acs.jctc.7b00118. 28418654

[ref7] Boussessi R.; Ceselin G.; Tasinato N.; Barone V. DFT meets the segmented polarization consistent basis sets: Performances in the computation of molecular structures, rotational and vibrational spectroscopic properties. J. Mol. Struct. 2020, 1208, 127886 10.1016/j.molstruc.2020.127886.

[ref8] Barone V.; Lupi J.; Salta Z.; Tasinato N. Development and Validation of a Parameter-Free Model Chemistry for the Computation of Reliable Reaction Rates. J. Chem. Theory Comput. 2021, 17, 4913–4928. 10.1021/acs.jctc.1c00406. 34228935 PMC8359010

[ref9] Karton A.; Spackman P. R. Evaluation of density functional theory for a large and diverse set of organic and inorganic equilibrium structures. J. Comput. Chem. 2021, 42, 1590–1601. 10.1002/jcc.26698. 34121198

[ref10] Grimme S. A General Quantum Mechanically Derived Force Field (QMDFF) for Molecules and Condensed Phase Simulations. J. Chem. Theory Comput. 2014, 10, 4497–4514. 10.1021/ct500573f. 26588146

[ref11] Bak K. L.; Gauss J.; Jørgensen P.; Olsen J.; Helgaker T.; Stanton J. F. Evaluation of density functional theory for a large and diverse set of organic and inorganic equilibrium structures. Int. J. Quantum Chem. 2021, 42, 1590–1601. 10.1002/jcc.26698 34121198

[ref12] Alessandrini S.; Barone V.; Puzzarini C. Extension of the ″Cheap″ Composite Approach to Noncovalent Interactions: The jun-ChS Scheme. J. Chem. Theory Comput. 2019, 16, 988–1006. 10.1021/acs.jctc.9b01037. 31860293

[ref13] Tamassia F.; Cané E.; Fusina L.; Di Lonardo G. The experimental equilibrium structure of acetylene. Phys. Chem. Chem. Phys. 2016, 18, 1937–1944. 10.1039/c5cp05997f. 26687993

[ref14] Tasinato N.; Stoppa P.; Pietropolli Charmet A.; Giorgianni S.; Gambi A. Modelling the anharmonic and Coriolis resonances within the six level polyad involving the *ν*_4_ fundamental in the ro-vibrational spectrum of vinyl fluoride. J. Quant. Spectrosc. Radiat. Transf. 2016, 18, 1937–1944.

[ref15] Pulay P.; Meyer W.; Boggs J. E. Cubic force constants and equilibrium geometry of methane from Hartree-Fock and correlated wavefunctions. J. Chem. Phys. 1978, 68, 5077–5085. 10.1063/1.435626.

[ref16] Pawłowski F.; Jørgensen P.; Olsen J.; Hegelund F.; Helgaker T.; Gauss J.; Bak K. L.; Stanton J. F. Cubic force constants and equilibrium geometry of methane from Hartree–Fock and correlated wavefunctions. J. Chem. Phys. 2002, 116, 6482–6496.

[ref17] Papoušek D.; Aliev M. R. Molecular Vibrational/rotational Spectra; Elsevier: Amsterdam, 1982.

[ref18] Mills I. M. Molecular Spectroscopy: Modern Research; Rao K. N.; Mathews C. W., Eds.; Academic Press: New York, 1972; pp 115–140.

[ref19] Piccardo M.; Penocchio E.; Puzzarini C.; Biczysko M.; Barone V. Semi-Experimental Equilibrium Structure Determinations by Employing B3LYP/SNSD Anharmonic Force Fields: Validation and Application to Semirigid Organic Molecules. J. Phys. Chem. A 2015, 119, 2058–2082. 10.1021/jp511432m. 25648634

[ref20] Penocchio E.; Piccardo M.; Barone V. Semiexperimental Equilibrium Structures for Building Blocks of Organic and Biological Molecules: The B2PLYP Route. J. Chem. Theory Comput. 2015, 11, 4689–4707. 10.1021/acs.jctc.5b00622. 26574259

[ref21] Penocchio E.; Mendolicchio M.; Tasinato N.; Barone V. Structural features of the carbon-sulfur chemical bond: a semi-experimental perspective. Can. J. Chem. 2016, 94, 1065–1076. 10.1139/cjc-2016-0282. 28912608 PMC5595238

[ref22] Barone V.; Ceselin G.; Fusè M.; Tasinato N. Accuracy Meets Interpretability for Computational Spectroscopy by Means of Hybrid and Double-Hybrid Functionals. Front. Chem. 2020, 8, 584203 10.3389/fchem.2020.584203. 33195078 PMC7645164

[ref23] Sim G.; Sutton L. E.; Bartell L.; Romenesko D.; Wong T. C. Augmented Analyses: Method of Predicate Observations; Royal Society of Chemistry’s, 1975.

[ref24] Demaison J.; Craig N. C.; Cocinero E. J.; Grabow J.-U.; Lesarri A.; Rudolph H. D. Semiexperimental Equilibrium Structures for the Equatorial Conformers of N-Methylpiperidone and Tropinone by the Mixed Estimation Method. J. Phys. Chem. A 2012, 116, 8684–8692. 10.1021/jp304178n. 22861349

[ref25] Zhao Y.; Truhlar D. G. Design of Density Functionals That Are Broadly Accurate for Thermochemistry, Thermochemical Kinetics, and Nonbonded Interactions. J. Phys. Chem. A 2005, 109, 5656–5667. 10.1021/jp050536c. 16833898

[ref26] Papajak E.; Zheng J.; Xu X.; Leverentz H. R.; Truhlar D. G. Perspectives on Basis Sets Beautiful: Seasonal Plantings of Diffuse Basis Functions. J. Chem. Theory Comput. 2011, 7, 3027–3034. 10.1021/ct200106a. 26598144

[ref27] Santra G.; Sylvetsky N.; Martin J. M. L. Minimally Empirical Double-Hybrid Functionals Trained against the GMTKN55 Database: revDSD-PBEP86-D4, revDOD-PBE-D4, and DOD-SCAN-D4. J. Phys. Chem. A 2019, 123, 5129–5143. 10.1021/acs.jpca.9b03157. 31136709 PMC9479158

[ref28] Grimme S. Semiempirical hybrid density functional with perturbative second-order correlation. J. Chem. Phys. 2006, 124, 034108 10.1063/1.2148954. 16438568

[ref29] Biczysko M.; Panek P.; Scalmani G.; Bloino J.; Barone V. Harmonic and Anharmonic Vibrational Frequency Calculations with the Double-Hybrid B2PLYP Method: Analytic Second Derivatives and Benchmark Studies. J. Chem. Theory Comput. 2010, 6, 2115–2125. 10.1021/ct100212p. 26615939

[ref30] Boussessi R.; Tasinato N.; Pietropolli Charmet A.; Stoppa P.; Barone V. Sextic centrifugal distortion constants: interplay of density functional and basis set for accurate yet feasible computations. Mol. Phys. 2020, 118, e1734678 10.1080/00268976.2020.1734678.

[ref31] Tasinato N.; Puzzarini C.; Barone V. Correct Modeling of Cisplatin: a Paradigmatic Case. Angew. Chem., Int. Ed. 2017, 56, 13838–13841. 10.1002/anie.201707683. PMC5656895 28857397

[ref32] Spada L.; Tasinato N.; Bosi G.; Vazart F.; Barone V.; Puzzarini C. On the competition between weak O H···F and C H···F hydrogen bonds, in cooperation with C H···O contacts, in the difluoromethane - tert-butyl alcohol cluster. J. Mol. Spectrosc. 2017, 337, 90–95. 10.1016/j.jms.2017.04.001. 28919646 PMC5597040

[ref33] Grimme S.; Antony J.; Ehrlich S.; Krieg H. A consistent and accurate ab initio parametrization of density functional dispersion correction (DFT-D) for the 94 elements H-Pu. J. Chem. Phys. 2010, 132, 154104 10.1063/1.3382344. 20423165

[ref34] Grimme S.; Ehrlich S.; Goerigk L. Effect of the damping function in dispersion corrected density functional theory. J. Comput. Chem. 2011, 32, 1456–1465. 10.1002/jcc.21759. 21370243

[ref35] Tasinato N.; Grimme S. Unveiling the non-covalent interactions of molecular homodimers by dispersion-corrected DFT calculations and collision-induced broadening of ro-vibrational transitions: Application to (CH_2_F_2_)_2_ and (SO_2_)_2_. Phys. Chem. Chem. Phys. 2015, 17, 5659–5669. 10.1039/c4cp05680a. 25623466

[ref36] Pritchard B. P.; Altarawy D.; Didier B.; Gibson T. D.; Windus T. L. New Basis Set Exchange: An Open, Up-to-Date Resource for the Molecular Sciences Community. J. Chem. Inf. Model. 2019, 59, 4814–4820. 10.1021/acs.jcim.9b00725. 31600445

[ref37] Barone V. Anharmonic vibrational properties by a fully automated second-order perturbative approach. J. Chem. Phys. 2005, 122, 014108 10.1063/1.1824881. 15638643

[ref38] Bloino J.; Biczysko M.; Barone V. General Perturbative Approach for Spectroscopy, Thermodynamics, and Kinetics: Methodological Background and Benchmark Studies. J. Chem. Theory Comput. 2012, 8, 1015–1036. 10.1021/ct200814m. 26593363

[ref39] Frisch M. J.; Gaussian16, Revision C.01.; Gaussian Inc: Wallingford CT, 2016.

[ref40] Bloino J.; Biczysko M.; Barone V. Anharmonic Effects on Vibrational Spectra Intensities: Infrared, Raman, Vibrational Circular Dichroism, and Raman Optical Activity. J. Phys. Chem. A 2015, 119, 11862–11874. 10.1021/acs.jpca.5b10067. 26580121 PMC5612400

[ref41] Mendolicchio M.; Penocchio E.; Licari D.; Tasinato N.; Barone V. Development and Implementation of Advanced Fitting Methods for the Calculation of Accurate Molecular Structures. J. Chem. Theory Comput. 2017, 13, 3060–3075. 10.1021/acs.jctc.7b00279. 28437115

[ref42] Becke A. D. Density-functional thermochemistry. III. The role of exact exchange. J. Chem. Phys. 1993, 98, 5648–5652. 10.1063/1.464913.

[ref43] Lee C.; Yang W.; Parr R. G. Development of the Colle-Salvetti correlation-energy formula into a functional of the electron density. Phys. Rev. B: Condens. Matter Mater. Phys. 1988, 37, 785–789. 10.1103/physrevb.37.785. 9944570

[ref44] Carnimeo I.; Puzzarini C.; Tasinato N.; Stoppa P.; Pietropolli Charmet A.; Biczysko M.; Cappelli C.; Barone V. Anharmonic theoretical simulations of infrared spectra of halogenated organic compounds. J. Chem. Phys. 2013, 139, 074310 cited By (59) 10.1063/1.4817401. 23968095 PMC4604659

[ref45] Müller H. S. P.; Thorwirth S.; Lewen F. Rotational spectroscopy of singly ^1^3C substituted isotopomers of propyne and determination of a semi-empirical equilibrium structure. J. Mol. Struct. 2020, 1207, 127769 10.1016/j.molstruc.2020.127769.

[ref46] Demaison J.; Császár A. G.; Dehayem-Kamadjeu A. The Case of the Weak N-X Bond: Ab Initio, Semi-Experimental, and Experimental Equilibrium Structures of XNO (X= H, F, Cl, OH) and FNO_2_. J. Phys. Chem. A 2006, 110, 13609–13617. 10.1021/jp064769v. 17165889

[ref47] Margulès L.; Demaison J.; Sreeja P. B.; Guillemin J.-C. Submillimeterwave spectrum of CH_2_PH and equilibrium structures of CH_2_PH and CH_2_NH. J. Mol. Struct. 2006, 238, 234–240. 10.1016/j.jms.2006.05.008.

[ref48] Jahn M. K.; Obenchain D. A.; Nair K. P. R.; Grabow J.-U.; Vogt N.; Demaison J.; Godfrey P. D.; McNaughton D. The puzzling hyper-fine structure and an accurate equilibrium geometry of succinic anhydride. Phys. Chem. Chem. Phys. 2020, 22, 5170–5177. 10.1039/c9cp06775b. 32083625

[ref49] Demaison J.; Craig N. C.; Conrad A. R.; Tubergen M. J.; Rudolph H. D. Semiexperimental Equilibrium Structure of the Lower Energy Conformer of Glycidol by the Mixed Estimation Method. J. Phys. Chem. A 2012, 116, 9116–9122. 10.1021/jp305504x. 22894798

[ref50] Müller H. S. P.; Brahmi M. A.; Guillemin J.-C.; Lewen F.; Schlemmer S. Rotational spectroscopy of isotopic cyclopropenone, *c*-H_2_C_3_O, and determination of its equilibrium structure. Astron. Astrophys. 2021, 647, A179 10.1051/0004-6361/202040088.

[ref51] Demaison J.; Margulès L.; Rudolph H. D. Accurate determination of an equilibrium structure in the presence of a small coordinate: The case of dimethylsulfide. J. Mol. Struct. 2010, 978, 229–233. 10.1016/j.molstruc.2010.02.025.

[ref52] Vogt N.; Demaison J.; Rudolph H. D. Semiexperimental equilibrium structure of the oblate-top molecules dimethyl sulfoxide and cyclobutene. J. Mol. Spectrosc. 2014, 297, 11–15. 10.1016/j.jms.2014.01.005.

[ref53] Demaison J.; Vogt N.; Ksenafontov D. N. Accuracy of semiexperimental equilibrium structures: Sulfine as an example. J. Mol. Struct. 2020, 1206, 127676 10.1016/j.molstruc.2019.127676.

[ref54] Ye H.; Mendolicchio M.; Kruse H.; Puzzarini C.; Biczysko M.; Barone V. The challenging equilibrium structure of HSSH: Another success of the rotational spectroscopy / quantum chemistry synergism. J. Mol. Struct. 2020, 1211, 127933 10.1016/j.molstruc.2020.127933.

[ref55] Demaison J.; Vogt N.; Saragi R. T.; Juanes M.; Rudolph H. D.; Lesarri A. How flexible is the disulfide linker? A combined rotational-computational investigation of diallyl disulfide. Phys. Chem. Chem. Phys. 2019, 21, 19732–19736. 10.1039/c9cp02508a. 31192318

[ref56] Demaison J.; Vogt N.; Saragi R. T.; Juanes M.; Rudolph H. D.; Lesarri A. The S–S Bridge: A Mixed Experimental-Computational Estimation of the Equilibrium Structure of Diphenyl Disulfide. ChemPhysChem 2019, 20, 366–373. 10.1002/cphc.201800973. 30476349

[ref57] degli Esposti C.; Melosso M.; Bizzocchi L.; Tamassia F.; Dore L. Determination of a semi-experimental equilibrium structure of 1-phosphapropyne from millimeter-wave spectroscopy of CH_3_CP and CD_3_CP. J. Mol. Struct. 2020, 1203, 127429 10.1016/j.molstruc.2019.127429.

[ref58] Ozeki H.; Saito S. Microwave spectra of HPO and DPO: molecular structure. J. Mol. Spectrosc. 2003, 219, 305–312. 10.1016/s0022-2852(03)00052-3.

[ref59] Demaison J.; Liévin J.; Császár A. G.; Gutle C. Equilibrium structure and torsional barrier of NH_3_BH_3_. J. Phys. Chem. A 2008, 112, 4477–4482. 10.1021/jp710630j. 18422295

[ref60] Gambi A.; Pietropolli Charmet A.; Stoppa P.; Tasinato N.; Ceselin G.; Barone V. Molecular synthons for accurate structural determinations: The equilibrium geometry of 1-chloro-1-fluoroethene. Phys. Chem. Chem. Phys. 2019, 21, 3615–3625. 10.1039/c8cp04888f. 30318548

[ref61] Demaison J.; Boggs J. E.; Rudolph H. D. Ab initio anharmonic force field and ab initio and experimental equilibrium structures of formyl chloride. J. Mol. Struct. 2004, 695–696, 145–153. 10.1016/j.molstruc.2003.10.035.

[ref62] Wu Q. Y.; Tan T. L. Fourier transform infrared (FTIR) spectroscopy of formaldoxime (CH_2_NOH) in the 450–3800 cm^–1^ region and its *ν*_9_ band. J. Mol. Spectrosc. 2021, 376, 1171417 10.1016/j.jms.2021.111417.

[ref63] Levine I. N. Structure of Formaldoxime. J. Chem. Phys. 1963, 38, 2326–2328. 10.1063/1.1733504.

[ref64] Shavitt I.; Bartlett R. J. Many-Body Methods in Chemistry and Physics, 1st ed.; Cambridge University Press, 2009.

[ref65] Stanton J. F.; Gauss J.; Harding M. E.; Szalay P. G. CFOUR. A Quantum Chemical Program Package; with contributions from A; Auer A.; Bartlett R. J.; Benedikt U.; Berger C.; Bernholdt D. E.; Bomble Y. J.; Christiansen O.; Engel F.; Heckert M.; Heun O.; Huber C.; Jagau T.-C.; Jonsson D.; Jusélius J.; Klein K.; Lauderdale W. J.; Lipparini F.; Matthews D.; Metzroth T.; Mück L. A.; O’Neill D. P.; Price D. R.; Prochnow E.; Puzzarini C.; Ruud K.; Schiffmann F.; Schwalbach W.; Stopkowicz S.; Tajti A.; Vázquez J.; Wang F.; Watts J. D., Eds. and the integral packages MOLECULE (Almlöf, J and P. R. Taylor); PROPS (Taylor, P. R.); ABACUS (Helgaker, T., Jensen, H. J. Aa.; P. Jørgensen, and J. Olsen), and ECP routines by A. V. Mitin and C. van Wüllen, Eds. For the current version, 2016 see http://www.cfour.de.

[ref66] Kawashima Y.; Takeo H.; Matsumura C. Structure of metastable molecules. The microwave spectra of BH(OH)2 and BHF(OH). J. Jpn. Soc. Chem. 1986, 1986, 1465–1475. 10.1246/nikkashi.1986.1465.

[ref67] Demaison J.; Herman M.; Lievin J. The equilibrium OH bond length. Int. Rev. Phys. Chem. 2007, 26, 391–420. 10.1080/01442350701371919.

[ref68] Kawashima Y.; Takeo H.; Matsumura C. Microwave spectrum of borinic acid BH_2_(OH). J. Chem. Phys. 1981, 74, 5430–5435. 10.1063/1.440947.

[ref69] Kumar A.; Sheridan J.; Stiefvater O. L. The Microwave Spectrum of Oxazole I. The Complete Structure by DRM Microwave Spectroscopy. Z. Naturforsch. 1978, 33, 145–152. 10.1515/zna-1978-0207.

[ref70] Stiefvater O. L. The complete structure of isoxazole from naturally occurring isotopic forms by double resonance modulated microwave spectroscopy. J. Chem. Phys. 1975, 63, 2560–2569. 10.1063/1.431647.

[ref71] Stiefvater O. L.; Nösberger P.; Sheridan J. Microwave spectrum and structure of isoxazole. Chem. Phys. 1975, 9, 435–444. 10.1016/0301-0104(75)80081-4.

[ref72] Kojima T. Potential Barrier of Phenol from its Microwave Spectrum. J. Phys. Soc. Jpn. 1960, 15, 284–287. 10.1143/jpsj.15.284.

[ref73] Forest H.; Dailey B. P. Microwave Spectra of Some Isotopically Substituted Phenols. J. Chem. Phys. 1966, 45, 1736–1746. 10.1063/1.1727823.

[ref74] Pedersen T.; Larsen N. W.; Nygaard L. Microwave spectra of the six monodeuteriophenols. Molecular structure, dipole moment, and barrier to internal rotation of phenol. J. Mol. Struct. 1969, 4, 59–77. 10.1016/0022-2860(69)85029-5.

[ref75] Larsen N. W. Microwave spectra of the six mono-^13^C-substituted phenols and of some monodeuterated species of phenol. Complete substitution structure and absolute dipole moment. J. Mol. Struct. 1979, 51, 175–190. 10.1016/0022-2860(79)80292-6.

[ref76] Salta Z.; Segovia M. E.; Katz A.; Tasinato N.; Barone V.; Ventura O. N. Isomerization and Fragmentation Reactions on the [C_2_SH_4_] Potential Energy Surface: The Metastable Thione S-Methylide Isomer. J. Org. Chem. 2021, 86, 2941–2956. 10.1021/acs.joc.0c02835. 33501826 PMC8023414

[ref77] Salta Z.; Lupi J.; Tasinato N.; Barone V.; Ventura O. N.; Ventura O. N. Unraveling the role of additional OH-radicals in the H-Abstraction from Dimethyl sulfide using quantum chemical computations. Chem. Phys. Lett. 2020, 739, 136963 10.1016/j.cplett.2019.136963.

[ref78] Esselman B. J.; Kougias S. M.; Zdanovskaia M. A.; Woods R. C.; McMahon R. J. Synthesis, Purification, and Rotational Spectroscopy of (Cyanomethylene)Cyclopropane-An Isomer of Pyridine. J. Phys. Chem. A 2021, 125, 5601–5614. 10.1021/acs.jpca.1c03246. 34153184

[ref79] Melli A.; Melosso M.; Tasinato N.; Bosi G.; Spada L.; Bloino J.; Mendolicchio M.; Dore L.; Barone V.; Puzzarini C. Rotational and Infrared Spectroscopy of Ethanimine: A Route toward Its Astrophysical and Planetary Detection. Astrophys. J. 2018, 855, 123 10.3847/1538-4357/aaa899.

[ref80] Spada L.; Tasinato N.; Vazart F.; Barone V.; Caminati W.; Puzzarini C. Noncovalent Interactions and Internal Dynamics in Pyridine-Ammonia: A Combined Quantum-Chemical and Microwave Spectroscopy Study. Chem.—Eur. J. 2017, 23, 4876–4883. 10.1002/chem.201606014. 28186344

[ref81] McNaughton D.; Wachsmuth D.; Kraus P.; Herbers S.; Wang J.; Grabow J.-U. Determination of the ″Privileged Structure″ of 8-Hydroxyquinoline. ChemPhysChem 2021, 22, 1692 10.1002/cphc.202100384. 34132015

[ref82] Maris A.; Michela Giuliano B.; Melandri S.; Ottaviani P.; Caminati W.; Favero L. B.; Velino B. Structure, dipole moment and large amplitude motions of 1-benzofuran. Phys. Chem. Chem. Phys. 2005, 7, 3317–3322. 10.1039/b507795h. 16240046

[ref83] Vogt N.; Demaison J.; Krasnoshchekov S. V.; Stepanov N. F.; Rudolph H. D. Determination of accurate semiexperimental equilibrium structure of proline using efficient transformations of anharmonic force fields among the series of isotopologues. Mol. Phys. 2017, 115, 942–951. 10.1080/00268976.2017.1292370.

[ref84] Vogt N.; Demaison J.; Cocinero E. J.; Écija P.; Lesarri A.; Rudolph H. D.; Vogt J. The equilibrium molecular structures of 2-deoxyribose and fructose by the semiexperimental mixed estimation method and coupled-cluster computations. Phys. Chem. Chem. Phys. 2016, 18, 15555–15563. 10.1039/c6cp01842d. 27212641

[ref85] Sanz M. E.; Blanco S.; López J. C.; Alonso J. L. Rotational Probes of Six Conformers of Neutral Cysteine. Angew. Chem., Int. Ed. 2008, 47, 6216–6220. 10.1002/anie.200801337. 18618532

[ref86] Alonso J. L.; Peña I.; López J. C.; Vaquero V. Rotational Spectral Signatures of Four Tautomers of Guanine. Angew. Chem., Int. Ed. 2009, 48, 6141–6143. 10.1002/anie.200901462. 19565585

[ref87] Puzzarini C.; Bloino J.; Tasinato N.; Barone V. Accuracy and Interpretability: The Devil and the Holy Grail. New Routes across Old Boundaries in Computational Spectroscopy. Chem. Rev. 2019, 119, 8131–8191. 10.1021/acs.chemrev.9b00007. 31187984

[ref88] Puzzarini C.; Tasinato N.; Bloino J.; Spada L.; Barone V. State-of-the-art computation of the rotational and IR spectra of the methyl-cyclopropyl cation: hints on its detection in space. Phys. Chem. Chem. Phys. 2019, 21, 3615–3625. 10.1039/c8cp04629h. 30110028

[ref89] Heim Z. N.; Amberger B. K.; Esselman B. J.; Stanton J. F.; Woods R. C.; McMahon R. J. Molecular structure determination: Equilibrium structure of pyrimidine (m-C_4_H_4_N_2_) from rotational spectroscopy (*r*_e_^SE^) and high-level ab initio calculation (*r*_*e*_) agree within the uncertainty of experimental measurement. J. Chem. Phys. 2020, 152, 104303 10.1063/1.5144914. 32171207

