# Supplementary material for: Accurate Biomolecular Structures by the Nano-LEGO Approach: Pick the Bricks and Build Your Geometry
Source: J Chem Theory Comput. 2021 Oct 20;17(11):7290–311. doi: 10.1021/acs.jctc.1c00788 (PMC8582257; doi:10.1021/acs.jctc.1c00788)
Supplement: Supplementary file 1 — ct1c00788_si_001.pdf [file ct1c00788_si_001.pdf]

Supporting Information: Accurate biomolecular structures by the nano-LEGO approach: pick the bricks and build your geometry

Giorgia Ceselin, Vincenzo Barone, and Nicola Tasinato*

Scuola Normale Superiore, Piazza dei Cavalieri 7, I-56126, Pisa, Italy

E-mail: nicola.tasinato@sns.it

Table S1: Semi-experimental, rev-DSDPBEP86 and PW6B95 equilibrium geometries of the molecules contained in the SE100 database[a]

Molecule	Parameter	SE	rev-DSD	PW6
HCN	r(C2≡N1)	1.1533	1.1575	1.1527
	r(C2−H3)	1.0651	1.0674	1.0713
HNC	r(C1≡N2)	1.1685	1.1717	1.1724
	r(N2−H3)	0.9954	0.9973	1.0001
HCO+	r(C2≡O1)	1.1057	1.1107	1.1046
	r(C2−H3)	1.0919	1.0948	1.1005
CO_2	r(C2=O1)	1.1600	1.1651	1.1616
H_2O	r(O1−H2)	0.9573	0.9610	0.9593
	α(H2O1H2)	104.53	104.46	105.01
HNO	r(N2=O1)	1.2087	1.2095	1.1971
	r(N2−H3)	1.0536	1.0554	1.0603
	α(H3N2O1)	108.12	108.18	108.50
HOF	r(F1−O1)	1.4345	1.4303	1.4168
	r(O2−H3)	0.9686	0.9697	0.9687
	α(H3O2F1)	97.86	98.13	98.70
HPO	r(P2=O1)	1.4806	1.4896	1.4953
	r(P2−H3)	1.4543	1.4569	1.4660
	α(H3P2O1)	104.27	104.35	103.80
SO_2	r(S2=O1)	1.4308	1.4421	1.4455
	α(O1S2O1)	119.31	119.28	119.08
C_2H_2	r(C1≡C1)	1.2030	1.2062	1.2065
	r(C1−H2)	1.0617	1.0639	1.0685
H_2CO	r(C2=O1)	1.2047	1.2072	1.2015
	r(C2−H3)	1.1003	1.1041	1.1098
	α(H3C2O1)	121.65	121.78	121.82
NH_3	r(N1−H2)	1.0110	1.0137	1.0142
	α(H2N1H2)	106.94	106.70	106.62
HNCO	r(C2=O1)	1.1644	1.1685	1.1655
	r(C2=N3)	1.2147	1.2180	1.2164
	r(N3−H4)	1.0059	1.0058	1.0074
	α(C2N3H4)	123.02	123.60	122.82
	α(N3C2O1)	171.51	172.20	172.64
HNCS	r(C2=S1)	1.5678	1.5711	1.5681
	r(C2=N3)	1.2047	1.2068	1.2070
	r(N3−H4)	1.0070	1.0050	1.0072
	α(S1C2N3)	172.23	173.78	173.82
	α(C2N3H4)	129.75	131.33	129.06
CH_2S	r(C2=S1)	1.6093	1.6111	1.6043
	r(C2−H3)	1.0854	1.0884	1.0941
	α(H3C2S1)	121.73	121.92	122.15
PH_3	r(P1−H2)	1.4117	1.4154	1.4223
	α(H2P1H2)	93.42	93.73	93.01
SH_3+	r(S1−H2)	1.3500	1.3525	1.3596
	α(H2S1H2)	94.15	94.47	93.93
H_2S_2	r(S1−S1)	2.0513	2.0609	2.0596
	r(S1−H1)	1.3401	1.3422	1.3493
	α(H2S1S1)	98.07	98.24	98.35
	δ(H2S1S1H2)	90.72	90.63	90.73
HClC=O	r(C2−Cl3)	1.7628	1.7702	1.7650
	r(C2=O1)	1.1820	1.1857	1.1817
	r(C2−H4)	1.0918	1.0946	1.0998

	α(H4C2O1)	126.38	126.35	126.38
	α(Cl3C2O1)	123.33	123.54	123.43
	α(H4C2Cl3)	110.29	110.11	110.18
$H_2C{=}NH$	r(C1=N2)	1.2709	1.2721	1.2667
	r(N2−H3)	1.0192	1.0211	1.0217
	r(C1−H4)	1.0897	1.0932	1.0984
	r(C1−H5)	1.0861	1.0888	1.0941
	α(C1N2H3)	110.33	110.37	110.55
	α(H4C1N2)	124.18	124.47	124.68
	α(H5C1N2)	118.76	118.72	118.85
H_2CCO	r(C2=O1)	1.1606	1.1644	1.1622
	r(C2=C3)	1.3124	1.3156	1.3131
	r(C3−H4)	1.0764	1.0786	1.0827
	α(C2C3H4)	119.05	119.16	119.37
CH_2O_2 (Dioxirane)	r(O1−O1)	1.5133	1.5099	1.4826
	r(C2−O1)	1.3846	1.3884	1.3839
	r(C2−H3)	1.0853	1.0880	1.0921
	α(H3C2H3)	117.03	117.00	117.29
CH_2O_2 (cis Formic Acid)	r(C3=O1)	1.1911	1.1952	1.1929
	r(C3−O2)	1.3482	1.3520	1.3465
	r(O2−H5)	0.9612	0.9646	0.9627
	r(C3−H4)	1.0981	1.1016	1.1062
	α(O2C3O1)	122.28	122.41	122.34
	α(H4C3O1)	123.87	123.99	123.89
	α(C3O2H5)	109.18	109.27	109.84
CH_2O_2 (trans Formic Acid)	r(C3=O1)	1.1976	1.2019	1.1995
	r(C3−O2)	1.3411	1.3450	1.3398
	r(O2−H5)	0.9660	0.9697	0.9679
	r(C3−H4)	1.0918	1.0952	1.0997
	α(O1C3O2)	124.81	125.05	124.92
	α(H4C5O1)	125.14	125.11	125.11
	α(C3O2H5)	106.81	106.93	107.26
HNCCN+	r(C2=C3)	1.3724	1.3723	1.3693
	r(C2=N1)	1.1634	1.1683	1.1607
	r(C3=N4)	1.1406	1.1447	1.1438
	r(N4−H5)	1.0133	1.0157	1.0171
$H_2C{=}PH$	r(C1=P2)	1.6661	1.6685	1.6626
	r(P2−H3)	1.4195	1.4221	1.4291
	r(C1−H4)	1.0810	1.0834	1.0882
	r(C1−H5)	1.0829	1.0852	1.0899
	α(H3P2C1)	97.36	97.55	97.44
	α(H4C1P2)	124.58	124.70	125.17
	α(H5C1P2)	119.17	119.20	119.25
C_2H_2S	r(C2=S1)	1.5556	1.5589	1.5576
	r(C2=C3)	1.3107	1.3128	1.3100
	r(C3−H4)	1.0806	1.0825	1.0864
	α(C2C3H4)	120.15	120.28	120.45
CH_2F_2	r(C1−F2)	1.3532	1.3584	1.3620
	r(C1−H3)	1.0868	1.0899	1.0944
	α(F2C1F2)	108.29	108.35	108.21
	α(H3C1F2)	108.74	108.71	108.67
	α(H3C1H3)	113.48	113.52	113.83
$CHClF_2$	r(C1−Cl2)	1.7562	1.7651	1.7586
	r(C1−F3)	1.3360	1.3410	1.3451
	r(C1−H4)	1.0850	1.0870	1.0919

	α(H4C1Cl2)	109.56	109.45	109.80
	α(F3C2Cl2)	109.61	109.66	109.63
	α(F3C2F3)	108.09	108.10	107.91
	α(H4C1F3)	109.98	109.97	109.93
CH_2ClF	r(C3−Cl1)	1.7641	1.7723	1.7666
	r(C3−F2)	1.3594	1.3638	1.3678
	r(C3−H4)	1.0840	1.0869	1.0912
	α(F2C3Cl1)	110.02	110.13	109.97
	α(H4C3Cl1)	107.96	107.99	108.15
	α(H4C3H4)	112.57	112.64	112.86
CCl_2F_2	r(C1−Cl3)	1.7520	1.7611	1.7557
	r(C1−F2)	1.3285	1.3328	1.3369
	α(Cl3C1Cl3)	111.61	111.61	112.00
	α(Cl3C1F2)	109.34	109.33	109.28
	α(F2C1F2)	107.78	107.84	107.63
CH_2Cl_2	r(C1−Cl2)	1.7640	1.7710	1.7675
	r(C1−H3)	1.0816	1.0843	1.0883
	α(Cl2C1Cl2)	112.18	112.40	112.58
	α(H3C1Cl2)	108.23	108.20	108.11
	α(H3C1H3)	111.77	111.70	111.87
H_2NOH	r(N1−O2)	1.4409	1.4420	1.4293
	r(O2−H4)	0.9578	0.9615	0.9598
	r(N1−H3)	1.0138	1.0165	1.0169
	α(H4O2N1)	102.10	102.22	103.02
	α(O2N1H3)	103.62	103.78	104.03
	δ(H3N1O2H4)	124.85	124.84	124.74
BHFOH	r(B1−F3)	1.31961	1.3265	1.3390
	r(B1−O4)	1.34626	1.3537	1.3478
	r(B1−H2)	1.189385	1.1932	1.1973
	r(O4−H5)	0.95735	0.9603	0.9590
	α(H2B1F3)	119.381	119.37	119.14
	α(H2B1O4)	123.459	123.48	124.38
	α(B1O4H5)	112.714	112.76	113.07
BH_2OH	r(B1−O4)	1.34979	1.3569	1.3524
	r(B1−H2)	1.1957	1.1982	1.2045
	r(B1−H3)	1.1899	1.1926	1.1997
	r(O4−H5)	0.9558	0.9615	0.9600
	α(H2B1H3)	122.84	122.77	123.02
	α(H2B1O2)	119.80	120.49	120.35
	α(H3B1O2)	117.24	116.75	116.63
	α(B1O2H5)	112.90	112.95	113.18
$H_2C{=}S{=}O$ (Sulfine)	r(C3=S2)	1.6084	1.6116	1.6053
	r(S2=O1)	1.4650	1.4731	1.4825
	r(C3−H4)	1.0797	1.0828	1.0871
	r(C3−H5)	1.0817	1.0833	1.0875
	α(C3S2O1)	114.80	115.22	115.00
	α(S2C3H4)	123.05	123.31	123.55
	α(S2C3H5)	115.19	115.20	115.30
C_4H_2	r(C1≡C2)	1.2084	1.2124	1.2126
	r(C1−C1)	1.3727	1.3734	1.3686
	r(C2−H3)	1.0615	1.0641	1.0679
C_2H_4	r(C1=C1)	1.3311	1.3317	1.3290
	r(C1−H2)	1.0807	1.0835	1.0879
	α(C1C1H2)	121.42	121.50	121.66
	α(H2C1H2)	117.16	117.01	116.67

cyc-H_2C_3O (Cyclopropenone)	r(C2=O1)	1.20043(13)	1.2045	1.2024
	r(C2−C3)	1.42887	1.4320	1.4298
	r(C3−H4)	1.078173	1.0814	1.0862
	α(C3C2O1)	151.9272	151.93	151.91
	α(C2C3H4)	153.7200	153.78	153.86
CH_2O_3 (Peroxy Formic Acid)	r(O2−O3)	1.4390	1.4377	1.4205
	r(C4=O1)	1.2015	1.2051	1.2036
	r(C4−O2)	1.3368	1.3409	1.3350
	r(O3−H6)	0.9720	0.9813	0.9808
	r(C4−H5)	1.0905	1.0939	1.0979
	α(H5C4O1)	126.96	127.00	127.08
	α(H5C4O2)	108.72	108.48	108.89
	α(O1C4O2)	124.32	124.52	124.02
	α(C4O2O3)	110.31	110.67	110.99
	α(O2O3H6)	100.78	100.70	101.07
$C_2H_2O_2$ (Trans Glyoxal)	r(C2=O1)	1.2046	1.2086	1.2032
	r(C2−C2)	1.5157	1.5204	1.5176
	r(C2−H3)	1.1006	1.1040	1.1089
	α(C2C2H3)	115.23	115.17	115.26
	α(H3C2O1)	123.60	123.47	123.46
	α(C2C2O1)	121.17	121.36	121.28
H_2CCCO	r(C2−O1)	1.1662	1.1695	1.1672
	r(C2=C3)	1.3047	1.3086	1.3033
	r(C3=C4)	1.3299	1.3312	1.3263
	r(C4−H5)	1.0883	1.0879	1.0926
	r(C4−H6)	1.0874	1.0861	1.0908
	α(C3C2O1)	168.31	168.25	168.96
	α(C2C3C4)	142.46	142.18	145.43
	α(C3C4H5)	122.42	122.74	122.75
	α(C3C4H6)	120.62	120.88	121.18
H_2C=NOH	r(N2−O1)	1.39899	1.4005	1.3867
	r(C3=N2)	1.27112	1.2728	1.2679
	r(O1−H6)	0.9627	0.9627	0.9607
	r(C3−H4)	1.07952	1.0814	1.0858
	r(C3−H5)	1.08425	1.0873	1.0918
	α(C3N2O1)	110.548	110.87	111.31
	α(H6O1N2)	102.65	102.61	103.41
	α(H5C3N2)	122.238	122.47	122.54
	α(H4C3N2)	116.223	116.33	116.52
$CH_3C{\equiv}P$	r(C2≡P1)	1.5456	1.5522	1.5439
	r(C2−C3)	1.4620	1.4645	1.4555
	r(C3−H4)	1.0902	1.0924	1.0960
	α(C2C3H4)	110.56	110.62	110.76
C_3H_2S (Propadienthione)	r(C2=S1)	1.5715	1.5739	1.5708
	r(C2=C3)	1.2702	1.2731	1.2747
	r(C3=C4)	1.3228	1.3227	1.3194
	r(C4-H5)	1.0842	1.0870	1.0909
	α(C3C4H5)	121.20	121.47	121.61
CH_2CHF	r(C1−F3)	1.3424	1.3453	1.3490
	r(C1−C2)	1.3213	1.3235	1.3210
	r(C1−H4)	1.0792	1.0821	1.0862
	r(C2−H5)	1.0785	1.0811	1.0852
	r(C2−H6)	1.0772	1.0801	1.0841
	α(F3C1H4)	112.10	112.07	111.71
	α(F3C1C2)	121.72	121.96	121.96

	α(C1C2H5)	121.32	121.48	121.63
	α(C1C2H6)	118.95	118.94	119.05
ClFC=CH_2	r(C2−Cl4)	1.7081	1.7156	1.7108
	r(C2−F3)	1.3287	1.3322	1.3362
	r(C2=C1)	1.3233	1.3243	1.3222
	r(C1−H5)	1.0780	1.0803	1.0841
	r(C1−H6)	1.0750	1.0779	1.0817
	α(F3C2C1)	125.44	122.52	122.49
	α(Cl4C2C1)	125.52	125.62	126.04
	α(C2C1H5)	119.3	119.37	119.41
	α(C2C1H6)	120.02	120.13	120.27
cis-CHFCHCl	r(C2−Cl4)	1.7129	1.7175	1.7130
	r(C1−F3)	1.3313	1.3346	1.3376
	r(C1=C2)	1.3244	1.3275	1.3255
	r(C1−H5)	1.0796	1.0814	1.0852
	r(C2−H6)	1.0795	1.0791	1.0836
	α(Cl4C2C1)	123.08	123.44	123.45
	α(F3C1C2)	122.56	122.87	122.75
	α(C1C2H6)	121.08	120.25	120.48
	α(C2C1H5)	123.49	123.26	123.57
$BH(OH)_2$	r(B2−O3)	1.35353	1.3608	1.3575
	r(B2−O4)	1.36364	1.3709	1.3675
	r(O3−H5)	0.96034	0.9635	0.9621
	r(O4−H6)	0.95625	0.9596	0.9580
	r(B2−H1)	1.189723	1.1936	1.1986
	α(H1B2O3)	118.527	118.53	118.66
	α(O4B2O3)	119.1251	119.17	118.88
	α(B2O3H5)	111.9132	111.96	111.93
	α(B2O4H6)	112.85	112.83	113.09
H_2C=C=CH_2 (Allene)	r(C1=C1)	1.3066	1.3077	1.3063
	r(C1−H2)	1.0807	1.0837	1.0876
	α(H2C1H2)	118.26	118.13	117.86
C_2H_4O (Oxirane)	r(C2−O1)	1.4274	1.4316	1.4242
	r(C2−C2)	1.4609	1.4636	1.4626
	r(C2−H3)	1.0816	1.0848	1.0892
	α(C2O1C2)	61.56	61.48	61.79
	α(C2C2O1)	59.22	59.26	59.10
	α(H3C2O1)	114.87	114.89	115.01
	α(H3C2H3)	116.25	116.05	115.90
C_2H_4O (Ethenol)	r(C2−O1)	1.3596	1.3622	1.3569
	r(C2=C3)	1.3313	1.3330	1.3313
	r(O1−H4)	0.9603	0.9639	0.9619
	r(C2−H5)	1.0790	1.0825	1.0867
	r(C3−H6)	1.0772	1.0799	1.0837
	r(C3−H7)	1.0814	1.0841	1.0881
	α(C3C2O1)	126.28	126.61	126.85
	α(C2O1H4)	108.75	108.83	109.26
	α(C3C2H5)	122.51	122.60	122.58
	α(C2C3H6)	119.59	119.60	119.78
	α(C2C3H7)	121.88	122.06	122.20
$C_2H_2O_3$ (Glyoxilic Acid)	r(C4=O1)	1.1967	1.2025	1.1998
	r(C5=O3)	1.2054	1.2096	1.2049
	r(C4−O2)	1.3316	1.3350	1.3297
	r(C4−C5)	1.5244	1.5292	1.5249
	r(O2−H6)	0.9697	0.9732	0.9720

	r(C5−H7)	1.0966	1.0994	1.1040
	α(C5C4O1)	121.86	121.84	121.93
	α(C5C4O2)	113.48	113.55	113.34
	α(C4C5O3)	120.69	120.93	120.46
	α(C4O2H6)	107.1	107.09	107.27
	α(C4C5H7)	115.48	115.24	115.66
C_2H_4S (Thiirane)	r(C2−S1)	1.8111	1.8174	1.8091
	r(C2−H3)	1.0798	1.0826	1.0871
	α(C2S1C2)	48.25	48.18	48.25
	α(S1C2H3)	114.98	115.02	115.35
	α(H3C2H3)	115.66	115.48	114.98
$CH_3C{\equiv}CH$ (Propyne)	r(C3≡C4)	1.2046	1.2082	1.2090
	r(C2−C3)	1.4588	1.4615	1.4538
	r(C2−H1)	1.0884	1.0913	1.0944
	r(C4−H5)	1.0614	1.0634	1.0677
	α(C3C2H1)	110.59	110.64	110.73
C_2H_4NH (Aziridine)	r(C2−C2)	1.4772	1.4803	1.4783
	r(C2−N1)	1.4708	1.4735	1.4640
	r(N1−H3)	1.0124	1.0149	1.0144
	r(C2−H4)	1.0804	1.0836	1.0880
	r(C2−H5)	1.0797	1.0823	1.0869
	α(C2N1C2)	60.29	60.30	60.65
	α(C2C2N1)	59.86	59.85	59.68
	α(C2C2H4)	117.99	117.88	117.95
	α(C2C2H5)	119.36	119.64	119.79
	α(C2N1H3)	109.27	109.47	110.05
	α(H4C2N1)	118.43	117.88	118.71
	α(H5C2N1)	114.20	114.45	114.65
$C_2H_2N_2S$ (1,2,3-thiadiazole)	r(C5−S1)	1.6844	1.6882	1.6840
	r(S1−N2)	1.6845	1.6922	1.6995
	r(C4−N3)	1.3645	1.3642	1.3620
	r(N2−N3)	1.2855	1.2914	1.2760
	r(C4−H6)	1.0764	1.0796	1.0827
	r(C5−H7)	1.0753	1.0782	1.0823
	α(C5S1N2)	93.05	93.04	92.62
	α(S1N2N3)	111.29	111.12	111.09
	α(S1C5H7)	124.01	124.04	123.84
	α(N2N3C4)	113.90	113.99	114.49
	α(H6C4N3)	119.15	119.22	119.41
$C_2H_2N_2S$ (1,3,4-thiadiazole)	r(C2−S1)	1.7161	1.7204	1.7163
	r(C2=N3)	1.2987	1.3035	1.2986
	r(C2−H4)	1.0782	1.0798	1.0837
	α(C2S1C2)	86.31	86.34	86.35
	α(S1C2N3)	114.74	114.68	114.40
	α(S1C2H4)	122.25	122.54	122.55
$C_2H_2N_2S$ (1,2,4-thiadiazole)	r(C5−S1)	1.7031	1.7075	1.7044
	r(S1−N2)	1.6449	1.6522	1.6522
	r(C3−N2)	1.3146	1.3180	1.3119
	r(C5−N4)	1.3088	1.3129	1.3068
	r(C3−H6)	1.0784	1.0815	1.0848
	r(C5−H7)	1.0782	1.0810	1.0850
	α(C5S1N2)	92.74	92.73	92.52
	α(S1N2C3)	107.12	107.02	107.03
	α(N4C5S1)	112.37	112.34	112.39
	α(H6C3N2)	119.54	119.82	120.13

	α(S1C5H7)	123.90	123.83	123.61
$C_2H_2N_2S$ (1,2,5-thiadiazole)	r(S1−N2)	1.6254	1.6326	1.6329
	r(C3=N2)	1.3237	1.3288	1.3190
	r(C3−H4)	1.0785	1.0814	1.0852
	α(N2S1N2)	99.44	99.39	99.00
	α(S1N2C3)	106.56	106.48	106.72
	α(H4C3N2)	120.06	119.94	120.12
C_3H_4O (cis-Acrolein)	r(C2=O1)	1.2108	1.2142	1.2104
	r(C3=C4)	1.3350	1.3362	1.3346
	r(C2−C3)	1.4806	1.4848	1.4793
	r(C2−H5)	1.1024	1.1059	1.1106
	r(C3−H6)	1.0824	1.0848	1.0886
	r(C4−H7)	1.0808	1.0838	1.0882
	r(C4−H8)	1.0797	1.0824	1.0864
	α(C3C2O1)	123.96	124.28	124.25
	α(C2C3C4)	121.21	121.54	121.40
	α(C3C2H5)	115.83	115.52	115.59
	α(C4C3H6)	121.57	121.40	121.55
	α(C3C4H7)	119.85	120.01	119.87
	α(C3C4H8)	121.61	121.63	121.85
C_3H_4O (trans-Acrolein)	r(C2=O1)	1.2103	1.2139	1.2097
	r(C3=C4)	1.3354	1.3369	1.3351
	r(C2−C3)	1.4702	1.4732	1.4689
	r(C2−H5)	1.1048	1.1085	1.1132
	r(C3−H6)	1.0814	1.0841	1.0881
	r(C4−H7)	1.0825	1.0853	1.0895
	r(C4−H8)	1.0795	1.0824	1.0864
	α(C3C2O1)	124.02	124.20	124.29
	α(C2C3C4)	120.18	120.38	120.46
	α(C3C2H5)	115.08	114.86	114.85
	α(C4C3H6)	122.78	122.56	122.57
	α(C3C4H7)	120.46	120.57	120.59
	α(C3C4H8)	122.10	122.14	122.28
$C_2H_4O_2$ (cis-Methyl Formate)	r(C3=O1)	1.2003	1.2043	1.2019
	r(C3−O2)	1.3346	1.3384	1.3340
	r(C4−O2)	1.4347	1.4393	1.4343
	r(C4−H5)	1.0923	1.0959	1.0999
	r(C4−H6)	1.0870	1.0893	1.0928
	r(C4−H7)	1.0848	1.0861	1.0894
	α(O2C3O1)	125.47	125.72	125.34
	α(C4O2C3)	114.30	114.66	114.67
	α(H5C3O2)	109.58	109.36	109.70
	α(H6C4O2)	110.15	110.19	110.08
	α(H7C4O2)	105.40	105.50	105.62
	δ(H6C4O2C3)	-60.37	-60.35	-60.19
$C_2H_4O_2$ (Glycolaldehyde)	r(C4=O2)	1.2088	1.2117	1.2083
	r(C3−O1)	1.3965	1.4004	1.3928
	r(C3−C4)	1.5006	1.5051	1.4974
	r(O1−H5)	0.9611	0.9685	0.9679
	r(C3−H6)	1.0964	1.0988	1.1028
	r(C4−H7)	1.1010	1.1044	1.1087
	α(C4C3O1)	111.78	112.08	112.03
	α(C3C4O2)	121.65	121.99	121.58
	α(C3O1H5)	106.18	106.05	106.16
	α(C4C3H6)	107.91	107.85	107.77

	α(C3C4H7)	116.90	116.54	116.94
	δ(H6C3C4O2)	122.34	122.52	122.80
C_3H_3NS (Thiazole)	r(C2−S1)	1.7213	1.7241	1.7204
	r(C5−S1)	1.7089	1.7147	1.7094
	r(C4=C5)	1.3645	1.3668	1.3651
	r(C2=N3)	1.2986	1.3055	1.2998
	r(C2−H6)	1.0801	1.0808	1.0846
	r(C4−H7)	1.0774	1.0807	1.0841
	r(C5−H8)	1.0753	1.0782	1.0821
	α(C5S1C2)	89.24	89.25	89.29
	α(C4C5S1)	109.66	109.67	109.56
	α(S1C2N3)	115.33	115.29	115.16
	α(S1C2H6)	120.27	120.94	120.84
	α(S1C5H8)	121.83	121.78	121.72
	α(C5C4H7)	125.04	124.94	124.78
BH_3NH_3	r(B1−N2)	1.6453	1.6546	1.6415
	r(B1−H4)	1.2058	1.2108	1.2176
	r(N2−H3)	1.0101	1.0149	1.0150
	α(H4B1N2)	105.00	104.86	105.05
	α(H3N2B1)	110.97	110.96	111.08
C_3H_3NO (Oxazole)	r(C2−O1)	1.35097	1.3548	1.3487
	r(C4=C5)	1.34934	1.3531	1.3527
	r(C2=N3)	1.28819	1.2926	1.2897
	r(C4−N3)	1.39268	1.3945	1.3848
	r(C2−H6)	1.07481	1.0774	1.0800
	r(C4−H7)	1.07433	1.0767	1.0804
	r(C5−H8)	1.07300	1.0752	1.0785
	α(C4C5O1)	108.07	108.03	107.79
	α(N3C2O1)	115.076	114.93	114.66
	α(C2N3C4)	103.884	103.95	104.13
	α(C5C4N3)	109.003	109.05	109.06
	α(H6C2O1)	116.832	116.74	116.88
	α(H7C4N3)	121.867	121.93	122.06
	α(C4C5H8)	135.145	135.16	135.21
C_3H_3NO (Isoxazole)	r(N2−O1)	1.39274	1.3930	1.3802
	r(C4=C5)	1.3515	1.3577	1.3565
	r(C3−C4)	1.42101	1.4213	1.4191
	r(C3=N2)	1.30706	1.3121	1.3064
	r(C3−H6)	1.07590	1.0793	1.0831
	r(C4−H7)	1.07311	1.0759	1.0795
	r(C5−H8)	1.07556	1.0777	1.0813
	α(C4C5O1)	110.75	110.51	110.33
	α(C3N2O1)	105.518	105.41	105.48
	α(C5C4C3)	102.986	103.03	102.79
	α(C4C3N2)	112.159	112.20	112.16
	α(C3C4H7)	128.44	128.77	128.87
	α(C4C5H8)	133.52	133.64	133.62
	α(H6C3N2)	118.50	118.71	118.78
cyc-C_3H_6 (Cyclopropane)	r(C1−C1)	1.5024	1.5061	1.5015
	r(C1−H2)	1.0790	1.0817	1.0858
	α(H2C1H2)	114.87	114.75	114.32
$CH_3CH{=}CH_2$ (Propene)	r(C1=C2)	1.3315	1.3333	1.3313
	r(C2−C3)	1.4958	1.4985	1.4923
	r(C1−H4)	1.0824	1.0849	1.0892
	r(C1−H5)	1.0800	1.0830	1.0868

	r(C2−H6)	1.0844	1.0873	1.0915
	r(C3−H7)	1.0873	1.0914	1.0943
	r(C3−H8)	1.0917	1.0937	1.0968
	α(C1C2C3)	124.44	124.71	125.13
	α(C2C1H4)	121.17	121.25	121.38
	α(C2C1H5)	121.44	121.46	121.59
	α(C1C2H6)	119.07	118.79	118.64
	α(C2C3H7)	111.08	111.17	111.29
	α(C2C3H8)	110.85	110.92	110.94
	δ(C1C2C3H8)	120.70	120.66	120.79
$C_3H_3N_2H$ (Pyrazole)	r(C4=C5)	1.3761	1.3803	1.3779
	r(C3−C4)	1.4087	1.4111	1.4095
	r(C3=N2)	1.3289	1.3339	1.3272
	r(C5−N1)	1.3516	1.3550	1.3515
	r(N1−N2)	1.3438	1.3454	1.3378
	r(N1−H6)	1.0016	1.0049	1.0054
	r(C3−H7)	1.0761	1.0786	1.0822
	r(C4−H8)	1.0741	1.0769	1.0805
	r(C5−H9)	1.0749	1.0777	1.0809
	α(C3C4C5)	104.47	104.53	104.39
	α(C4C3N2)	111.92	111.86	111.82
	α(C4C5N1)	106.26	106.17	106.10
	α(C3N2N1)	104.11	104.09	104.29
	α(C5N1N2)	113.24	113.34	113.40
	α(C3C4H8)	128.20	128.27	128.32
	α(H7C3N2)	119.42	119.44	119.53
	α(H9C5N1)	121.70	121.80	121.89
	α(N2N1H6)	118.87	118.92	118.99
$C_3H_4N_2$ (Imidazole)	r(C4=C5)	1.3624	1.3704	1.3696
	r(C2=N3)	1.3101	1.3148	1.3110
	r(C4−N3)	1.3792	1.3795	1.3710
	r(C5−N1)	1.3741	1.3775	1.3721
	r(N1−N2)	1.3613	1.3630	1.3594
	r(N1−H6)	1.0016	1.0052	1.0057
	r(C2−H7)	1.0772	1.0785	1.0811
	r(C4−H8)	1.0755	1.0778	1.0813
	r(C5−H9)	1.0765	1.0765	1.0795
	α(C4C5N1)	105.42	105.15	105.03
	α(C5C4N3)	110.62	110.62	110.63
	α(C2N3C4)	105.03	105.21	105.37
	α(C5N1C2)	107.01	107.30	107.37
	α(N1C2N3)	111.92	111.72	111.60
	α(C2N1H6)	126.20	126.36	126.30
	α(H7C2N1)	122.34	122.39	122.41
	α(H8C4N3)	121.48	121.44	121.57
	α(H9C5N1)	121.94	122.28	122.38
C_4H_4O (Furan)	r(C2−O1)	1.3598	1.3624	1.3554
	r(C2=C3)	1.3551	1.3588	1.3576
	r(C3−C3)	1.4317	1.4353	1.4306
	r(C2−H4)	1.0731	1.0764	1.0797
	r(C3−H5)	1.0751	1.0776	1.0814
	α(C2O1C2)	106.61	106.69	106.98
	α(C3C2O1)	110.66	110.62	110.52
	α(C2C3C3)	106.04	106.03	106.00
	α(H4C2O1)	115.88	115.85	116.00

	α(C3C3H5)	127.68	127.63	127.67
$(CH_3)_2O$ (Dimethyl Ether)	r(C2−O1)	1.4071	1.4108	1.4054
	r(C2−H3)	1.0858	1.0890	1.0926
	r(C2−H4)	1.0950	1.0978	1.1014
	α(C2O1C2)	111.10	111.44	111.88
	α(H3C2O1)	107.50	107.44	107.49
	α(H4C2O1)	111.14	111.23	111.27
	δ(C2O1C2H4)	60.52	60.58	60.58
$C_4H_2O_3$ (Maleic Anhydride)	r(C3=O2)	1.1896	1.1939	1.1917
	r(C3−O1)	1.3845	1.3890	1.3829
	r(C4=C4)	1.3326	1.3342	1.3324
	r(C3−C4)	1.4848	1.4884	1.4827
	r(C4−H5)	1.0760	1.0790	1.0829
	α(O1C3O2)	122.55	122.56	122.51
	α(C4C3O1)	107.81	107.72	107.79
	α(C4C3O2)	129.65	129.71	129.70
	α(C3O1C3)	108.51	108.52	108.55
	α(C4C4C3)	107.94	108.02	107.93
	α(C4C4H5)	129.86	129.81	129.88
$S(CH_3)_2$ [c]	r(C2−S1)	1.7986	1.8058	1.7996
	r(C2−H3)	1.0886	1.0900	1.0934
	r(C2−H4)	1.08972	1.0911	1.0951
	α(C2S1C2)	98.58	98.69	99.33
	α(S1C2H3)	107.4196	107.38	107.13
	α(S1C2H4)	110.688	110.87	111.16
	δ(H4C2S1C2)	119.053	118.88	118.73
C_4H_4S (Thiophene)	r(C2−S1)	1.7126	1.7154	1.7102
	r(C2=C3)	1.3622	1.3703	1.3677
	r(C3−C3)	1.4233	1.4239	1.4211
	r(C2−H4)	1.0771	1.0789	1.0828
	r(C3−H5)	1.0794	1.0814	1.0850
	α(C2S1C2)	91.88	92.06	92.12
	α(C3C2S1)	111.66	111.55	111.55
	α(C2C3C3)	112.40	112.42	112.39
	α(S1C2H4)	120.11	120.27	120.17
	α(C2C3H5)	124.14	124.23	124.23
CH_2=CH-CH=CH_2	r(C1=C2)	1.3380	1.3392	1.3376
	r(C2−C2)	1.4547	1.4571	1.4526
	r(C1−H3)	1.0817	1.0847	1.0885
	r(C1−H4)	1.0796	1.0824	1.0861
	r(C2−H5)	1.0842	1.0870	1.0909
	α(C1C2C2)	123.54	123.74	124.03
	α(C1C2H5)	119.81	119.62	119.49
	α(C2C1H3)	120.96	121.08	121.18
	α(C2C1H4)	121.45	121.54	121.63
cyc-C_4H_6 (Butene)	r(C1=C1)	1.3406	1.3423	1.3396
	r(C1−C2)	1.5141	1.5176	1.5101
	r(C2−C2)	1.5639	1.5682	1.5595
	r(C1−H3)	1.0805	1.0835	1.0875
	r(C2−H4)	1.0894	1.0923	1.0959
	α(C1C1C2)	94.23	94.27	94.17
	α(C1C1H3)	133.42	133.42	133.50
	α(C2C2H4)	114.64	115.70	114.77
	α(H4C2H4)	109.09	109.01	108.60
$C_3H_4O_3$ (Pyruvic Acid)	r(C4=O1)	1.1980	1.2033	1.2008

	r(C5=O3)	1.2115	1.2162	1.2124
	r(C4−O2)	1.3311	1.3344	1.3286
	r(C4−C5)	1.5382	1.5436	1.5394
	r(C5−C6)	1.4898	1.4934	1.4862
	r(O2−H9)	0.9678	0.9747	0.9738
	r(C6−H7)	1.0902	1.0922	1.0951
	r(C6−H8)	1.0819	1.0873	1.0894
	α(C4C5O3)	117.75	117.82	117.37
	α(C5C4O1)	122.87	122.93	122.91
	α(C5C4O2)	112.81	112.79	112.63
	α(C4C5C6)	116.93	116.82	117.06
	α(C4O2H9)	106.39	106.19	106.31
	α(C5C6H7)	109.26	109.38	109.28
	α(C5C6H8)	110.19	109.99	110.26
	δ(C4C5C6H7)	57.8	58.06	57.75
C_3H_6O (Propanal)	r(C2=O1)	1.2075	1.2105	1.2064
	r(C2−C3)	1.5022	1.5058	1.4990
	r(C3−C4)	1.5162	1.5211	1.5150
	r(C2−H5)	1.1057	1.1088	1.1135
	r(C3−H6)	1.0950	1.0971	1.1002
	r(C4−H7)	1.0884	1.0906	1.0935
	r(C4−H8)	1.0888	1.0909	1.0934
	α(C3C2O1)	124.37	124.65	124.58
	α(C2C3C4)	113.61	113.88	114.20
	α(C3C2H5)	115.45	115.15	115.26
	α(C2C3H6)	106.95	106.87	106.63
	α(C3C4H7)	110.72	110.78	110.77
	α(C3C4H8)	110.61	110.63	110.75
	δ(O1C2C3H6)	123.79	123.95	124.38
	δ(C2C3C4H7)	59.46	59.55	59.47
CH_2NH_2COOH (Glycine)	r(C3=O4)	1.2030	1.2068	1.2053
	r(C3−O5)	1.3490	1.3532	1.3480
	r(C2−N1)	1.4429	1.4477	1.4411
	r(C2−C3)	1.5133	1.5182	1.5120
	r(N1−H8)	1.0110	1.0136	1.0139
	r(O5−H6)	0.9650	0.9685	0.9662
	r(C2−H7)	1.0907	1.0930	1.0961
	α(O4C3O5)	123.03	123.07	122.87
	α(C3C2N1)	115.28	115.39	115.48
	α(C2C3O4)	125.53	125.68	125.39
	α(C2C3O5)	111.44	111.25	111.74
	α(C2N1H8)	109.90	109.82	109.85
	α(C3O5H6)	106.70	106.64	107.00
	α(C3C2H7)	107.39	107.53	107.52
	δ(H7C2C3O4)	123.20	123.18	123.47
	δ(H8N1C2C3)	57.40	57.86	57.78
C_4H_4NH (Pyrrole)	r(C2=C3)	1.3731	1.3772	1.3755
	r(C3−C3)	1.4226	1.4243	1.4203
	r(C2−N1)	1.3689	1.3721	1.3672
	r(C2−H5)	1.0746	1.0774	1.0804
	r(C3−H6)	1.0748	1.0783	1.0819
	r(N1−H4)	1.0012	1.0040	1.0045
	α(C2C3C4)	107.33	107.37	107.33
	α(C3C2N1)	107.75	107.68	107.67
	α(C2N1C2)	109.84	109.90	110.00
	α(C2N1H4)	125.08	125.05	125.00

	α(H5C2N1)	121.23	121.20	121.27
	α(C2C3H6)	125.88	125.67	125.68
$C_4H_4N_2$ (Pyridazine)	r(C3=C3)	1.3761	1.3799	1.3793
	r(C2−C3)	1.3938	1.3962	1.3929
	r(C2=N1)	1.3302	1.3331	1.3304
	r(C2−H4)	1.0810	1.0838	1.0874
	r(C3−H5)	1.0802	1.0827	1.0863
	α(C2C3C3)	116.85	116.82	116.78
	α(C3C2N1)	123.86	123.88	123.69
	α(C3C2H4)	121.35	121.29	121.37
	α(C3C3H5)	122.37	122.33	122.31
$C_4H_4N_2$ (Pyrimidine)	r(C3C4)	1.3868	1.3896	1.3880
	r(C2N1)	1.3333	1.3357	1.3315
	r(C3N1)	1.3350	1.3372	1.3323
	r(C2H5)	1.082	1.0845	1.0877
	r(C4H6)	1.0825	1.0851	1.0890
	r(C5H7)	1.0794	1.0819	1.0855
	α(C3C4C3)	116.70	116.66	116.50
	α(C2N1C3)	115.70	115.72	115.87
	α(C4C3N1)	122.27	122.29	122.28
	α(H5C2N1)	116.32	116.34	116.40
	α(H6C3N1)	116.52	116.48	116.58
	α(C3C4H7)	121.65	121.67	121.75
$(CH_3)_2S{=}O$	r(S2=O1)	1.4804	1.4896	1.5019
	r(C3−S2)	1.7964	1.8052	1.8035
	r(C3−H4)	1.0868	1.0894	1.0923
	r(C3−H5)	1.0880	1.0914	1.0945
	r(C3−H6)	1.0888	1.0911	1.0941
	α(O1S2C3)	106.55	106.68	106.57
	α(C3S2C3)	96.01	96.07	96.61
	α(S2C3H4)	106.96	106.89	106.67
	α(S2C3H5)	109.62	109.73	109.59
	α(S2C3H6)	108.48	108.76	109.07
	α(H4C3H6)	110.12	110.04	110.10
	α(H4C3H5)	110.25	109.99	109.91
	α(H4C3H6)	111.29	111.31	111.38
	δ(O1S2C3H4)	67.85	67.73	67.42
	δ(O1S2C3H6)	-50.9	-51.05	-51.47
	δ(O1S2C3H5)	-172.61	-173.03	-173.64
	δ(C3S2C3H4)	-177.13	-177.19	-176.93
	δ(C3S2C3H6)	-58.37	-58.41	-58.05
	δ(C3S2C3H5)	63.34	63.57	64.13
	δ(oriOSC)	-129.17	-129.09	-128.83
C_6H_5 (Phenyl-rad)	r(C1−C2)	1.3722	1.3682	1.3733
	r(C2−C3)	1.3984	1.3957	1.3995
	r(C3−C4)	1.3931	1.3885	1.3923
	r(C2−H5)	1.0808	1.0829	1.0869
	r(C3−H6)	1.0819	1.0844	1.0878
	r(C4−H7)	1.0809	1.0832	1.0866
	α(C2C1C2)	125.85	126.12	126.06
	α(C1C2C3)	116.60	116.37	116.38
	α(C2C3C4)	120.16	120.29	120.28
	α(C3C4C3)	120.63	120.56	120.62
	α(C1C2H5)	122.90	122.41	122.52
	α(C2C3H6)	120.39	119.59	119.57

	α(C3C4H7)	119.68	119.72	119.69
$C_4H_4O_3$ (Succinic Anhydride)	r(C2−O1)	1.3824	1.3869	1.3810
	r(C2=O5)	1.1878	1.1922	1.1902
	r(C2−C3)	1.5116	1.5157	1.5086
	r(C3−H4)	1.0900	1.0899	1.0926
	α(O5C2O1)	121.52	121.49	121.44
	α(C3C2O1)	109.77	109.67	109.55
	α(C3C2O5)	128.71	128.84	129.02
	α(C2O1C2)	111.32	111.38	111.55
	α(C2C3H4)	108.34	108.33	108.37
	δ(O1C2C3H4)	121.84	121.82	122.09
C_5H_5N (Pyridine)	r(C2C3)	1.3907	1.3929	1.3914
	r(C3C4)	1.3885	1.3912	1.3890
	r(C2N1)	1.3356	1.3379	1.3329
	r(C2−H5)	1.0821	1.0852	1.0889
	r(C3−H6)	1.0799	1.0828	1.0863
	r(C4−H7)	1.0806	1.0835	1.0871
	α(C2C3C4)	118.54	118.53	118.45
	α(C3C4C3)	118.43	118.45	118.49
	α(C2N1C2)	116.95	116.99	117.22
	α(C3C2N1)	123.77	123.75	123.69
	α(H5C2N1)	115.99	115.96	116.11
	α(C3C2H5)	120.24	120.29	120.19
	α(C2C3H6)	120.09	120.16	120.24
	α(C3C4H7)	120.78	120.78	120.75
C_5H_4NF (2-Fluoropyridine)	r(C2−F7)	1.3343	1.3388	1.3436
	r(C2−C3)	1.3888	1.3926	1.3889
	r(C3−C4)	1.3829	1.3868	1.3858
	r(C4−C5)	1.3945	1.3962	1.3933
	r(C5−C6)	1.3842	1.3874	1.3871
	r(C2−N1)	1.3085	1.3101	1.3066
	r(C6−N1)	1.3422	1.3437	1.3371
	r(C3−H8)	1.0787	1.0811	1.0844
	r(C4−H9)	1.0807	1.0831	1.0867
	r(C5−H10)	1.0794	1.0819	1.0853
	r(C6−H11)	1.0815	1.0841	1.0878
	α(C3C2F7)	118.06	117.93	117.95
	α(C2C3C4)	116.69	116.62	116.42
	α(C3C4C5)	119.07	119.12	119.21
	α(C4C5C6)	118.36	118.34	118.27
	α(C6N1C2)	116.30	116.42	116.60
	α(C3C2N1)	126.16	126.11	126.20
	α(C5C6N1)	123.42	123.40	123.30
	α(C2C3H8)	120.37	120.48	120.65
	α(C3C4H9)	120.36	120.15	120.10
	α(C6C5H10)	120.31	120.33	120.40
	α(C5C6H11)	120.88	120.87	120.76
C_5H_4NF(3-Fluoropyridine)	r(C3−F8)	1.3393	1.3439	1.3475
	r(C2−C3)	1.3857	1.3885	1.3861
	r(C3−C4)	1.3801	1.3834	1.3809
	r(C4−C5)	1.3883	1.3916	1.3892
	r(C5−C6)	1.3913	1.3927	1.3914
	r(C2−N1)	1.3356	1.3355	1.3304
	r(C6−N1)	1.3345	1.3378	1.3325
	r(C2−H7)	1.0817	1.0844	1.0879

	r(C4−H9)	1.0797	1.0824	1.0858
	r(C5−H10)	1.0800	1.0826	1.0861
	r(C6−H11)	1.0814	1.0842	1.0878
	α(C4C3F8)	119.92	119.85	119.78
	α(C2C3C4)	121.04	120.94	121.03
	α(C3C4C5)	116.91	116.90	116.82
	α(C4C5C6)	118.95	118.99	118.94
	α(C6N1C2)	117.63	117.69	117.93
	α(C3C2N1)	121.88	121.97	121.82
	α(C5C6N1)	123.58	123.51	123.45
	α(C3C4H9)	120.32	120.51	120.55
	α(C6C5H10)	120.39	120.31	120.38
	α(C5C6H11)	120.4	120.42	120.32
C_6H_6 (Benzene)	r(C1C1)	1.3916	1.3939	1.3918
	r(C1−H2)	1.0799	1.0837	1.0870
$C_4H_4N_2O_2$ (Uracil)	r(C2=O7)	1.2097	1.2141	1.2131
	r(C4=O8)	1.2124	1.2169	1.2152
	r(C4−C5)	1.4555	1.4571	1.4520
	r(C5=C6)	1.3440	1.3466	1.3463
	r(C2−N1)	1.3817	1.3859	1.3837
	r(C2−N3)	1.3763	1.3799	1.3749
	r(C4−N3)	1.3989	1.4040	1.4014
	r(C6−N1)	1.3712	1.3733	1.3684
	r(C5−H9)	1.0766	1.0783	1.0817
	r(C6−H10)	1.0793	1.0818	1.0851
	r(N1−H11)	1.0046	1.0067	1.0069
	r(N3−H12)	1.009	1.0109	1.0106
	α(C5C4O8)	125.81	126.00	126.16
	α(N1C2O7)	123.86	122.87	122.68
	α(C6C5C4)	119.55	119.64	119.62
	α(C2N1C6)	123.47	123.46	123.50
	α(C4N3C2)	127.98	128.08	128.07
	α(C5C6N1)	121.92	121.99	121.91
	α(C5C4N3)	113.79	113.66	113.71
	α(N3C2N1)	113.29	113.18	113.19
	α(C6C5H9)	122.08	121.86	121.94
	α(C2N1H11)	115.17	115.29	115.08
	α(C2N3H12)	115.58	115.57	115.62
	α(H10C6N1)	115.33	115.39	115.48
$C_4H_4N_2SO$ (2-Thiouracil)	r(C2=S8)	1.6501	1.6519	1.6496
	r(C6=O7)	1.213	1.2159	1.2141
	r(C5−C6)	1.4522	1.4551	1.4506
	r(C4=C5)	1.3448	1.3475	1.3470
	r(C2−N1)	1.3631	1.3664	1.3629
	r(C6−N1)	1.4017	1.4084	1.4048
	r(C2−N3)	1.3654	1.3715	1.3702
	r(C4−N3)	1.3714	1.3726	1.3672
	r(C5−H11)	1.0768	1.0785	1.0818
	r(C4−H10)	1.0796	1.0818	1.0849
	r(N3−H9)	1.0058	1.0076	1.0077
	r(N1−H12)	1.0098	1.0116	1.0110
	α(N3C2S8)	122.48	122.50	122.40
	α(C5C6O7)	126.17	126.37	126.49
	α(C4C5C6)	119.24	119.41	119.37
	α(C2N3C4)	123.69	123.72	123.81
	α(C6N1C2)	127.78	128.06	128.09

	α(C5C4N3)	121.67	121.75	121.70
	α(C5C6N1)	113.84	113.52	113.59
	α(N1C2N3)	113.78	113.53	113.44
	α(C5C4H10)	122.19	121.96	122.91
	α(C2N3H9)	115.18	115.26	114.99
	α(C2N1H12)	116.06	115.96	116.00
	α(N3C4H10)	115.29	115.31	115.38
C_6H_5OH (Phenol)	r(C1−O12)	1.36386	1.3681	1.3632
	r(C1−C2)	1.39197	1.3941	1.3927
	r(C2−C3)	1.39010	1.3937	1.3910
	r(C3−C4)	1.39018	1.3921	1.3902
	r(C4−C5)	1.39170	1.3951	1.3929
	r(C5−C6)	1.38882	1.3907	1.3884
	r(O10−H13)	0.95939	0.9625	0.9600
	r(C2−H7)	1.08363	1.0856	1.0888
	r(C3−H8)	1.08090	1.0836	1.0870
	r(C4−H9)	1.07960	1.0826	1.0859
	r(C5−H10)	1.08133	1.0836	1.0870
	r(C6−H11)	1.07972	1.0828	1.0859
	α(C2C1O12)	122.44	122.45	122.44
	α(C6C1O12)	117.14	117.28	117.27
	α(C1C2C3)	119.65	119.68	119.65
	α(C2C3C4)	120.50	120.50	120.56
	α(C3C4C5)	119.32	119.30	119.22
	α(C4C5C6)	120.75	120.74	120.84
	α(C1O12H13)	108.907	108.94	109.43
	α(C3C2H7)	120.507	120.31	120.38
	α(C4C3H8)	120.164	120.17	120.15
	α(C5C4H9)	120.37	120.37	120.43
	α(C6C5H10)	119.286	119.28	119.24
	α(C1C6H11)	119.05	119.04	119.24
CH_2=CH-CH=CH-CH=CH_2	r(C1=C2)	1.3418	1.3413	1.3399
	r(C2−C3)	1.4510	1.4534	1.4479
	r(C3=C3)	1.3509	1.3509	1.3507
	r(C1−H4)	1.0815	1.0848	1.0886
	r(C1−H5)	1.0800	1.0825	1.0860
	r(C2−H6)	1.0866	1.0848	1.0888
	r(C3−H7)	1.0813	1.0867	1.0900
	α(C1C2C3)	122.59	122.98	123.31
	α(C2C3C3)	126.23	126.40	126.69
	α(C2C1H4)	121.05	121.11	121.17
	α(C2C1H5)	121.38	121.50	121.62
	α(C1C2H6)	119.51	118.75	118.60
	α(C3C3H7)	117.48	117.89	117.75
C_6H_5-C≡CH (Phenyl-Acetylene)	r(C3−C4)	1.3988	1.4017	1.4007
	r(C4−C5)	1.3887	1.3908	1.3878
	r(C5−C6)	1.3916	1.3940	1.3919
	r(C2−C3)	1.4311	1.4322	1.4263
	r(C1−C2)	1.2075	1.2109	1.2107
	r(C1−H9)	1.0608	1.0638	1.0674
	r(C4−H10)	1.0788	1.0830	1.0860
	r(C5−H11)	1.0803	1.0834	1.0867
	r(C3−H12)	1.0801	1.0834	1.0866
	α(C4C3C4)	119.44	119.37	119.15
	α(C3C4C5)	120.12	120.16	120.27
	α(C4C5C6)	120.24	120.24	120.27

	α(C5C6C5)	119.84	119.84	119.77
	α(C3C4H10)	119.43	119.27	119.17
	α(C6C5H11)	120.14	120.08	120.10
	α(C5C4H10)	120.45	120.57	120.56
	α(C4C5H11)	119.62	119.68	119.63
$(H_2C{=}CH{-}CH_2S)_2$ (Diallyldisulfide)	r(S1−S10)	2.0282	2.0363	2.0365
	r(S1−C2)	1.8325	1.8351	1.8334
	r(S10−C11)	1.8332	1.8376	1.8375
	r(C2−C3)	1.4870	1.4906	1.4860
	r(C3=C4)	1.3337	1.3345	1.3324
	r(C12=C13)	1.3314	1.3340	1.3318
	r(C11−C12)	1.4851	1.4898	1.4849
	r(C4−H5)	1.0814	1.0843	1.0882
	r(C4−H6)	1.0806	1.0832	1.0868
	r(C3−H7)	1.0844	1.0867	1.0905
	r(C2−H8)	1.0923	1.0947	1.0974
	r(C2−H9)	1.0886	1.0904	1.0933
	r(C11−H14)	1.0874	1.0891	1.0918
	r(C11−H15)	1.0923	1.0948	1.0972
	r(C13−H16)	1.0803	1.0829	1.0865
	r(C12−H17)	1.0836	1.0862	1.0897
	r(C13−H18)	1.0816	1.0844	1.0885
	α(C2S1S10)	102.284	102.71	103.01
	α(C11S10S1)	103.910	103.58	103.66
	α(C3C2S1)	112.92	112.98	113.08
	α(C12C11S10)	112.24	112.14	112.38
	α(C2C3C4)	123.50	123.57	123.74
	α(C11C12C13)	123.27	123.45	123.52
	α(S1C2H9)	108.52	108.50	108.70
	α(S1C2H8)	103.37	103.21	102.96
	α(C2C3H7)	116.81	116.37	116.28
	α(C3C4H6)	121.29	121.41	121.55
	α(C3C4H5)	120.8	121.13	121.23
	α(C12C11H14)	112.02	112.13	112.11
	α(C12C11H15)	111.91	111.62	111.73
	α(C13C12H17)	120.00	120.04	119.96
	α(C12C13H16)	121.44	121.44	121.61
	α(C12C13H18)	120.89	121.13	121.20
	δ(S10S1C2C3)	68.93	67.83	66.53
	δ(S1S10C11C12)	-63.44	-64.52	-62.91
	δ(C2S1S10C11)	-93.63	-93.12	-92.62
	δ(S1C2C3C4)	-114.43	-113.62	-112.92
	δ(S10C11C12C13)	115.05	113.09	110.81
	δ(H6C4C3C2)	179.27	179.24	179.02
	δ(C2C3C4H5)	-1.14	-0.85	-1.13
	δ(H7C3C2S1)	67.79	64.79	65.21
	δ(H9C2S1S10)	-56.94	-56.49	-58.02
	δ(H8C2S1S10)	-172.27	-171.66	-173.03
	δ(H15C11C12C13)	-132.79	-131.94	-134.28
	δ(H16C13C12C11)	-178.69	-178.66	-178.32
	δ(H18C13C12C11)	0.67	1.26	1.54
	δ(H17C12C13C11)	177.44	177.59	177.21
	δ(H14C11C12C13)	-10.02	-9.56	-11.90
$(C_6H_5S)_2$ (Diphenyldisulfide)	r(S1−S1)	2.0199	2.0279	2.0315
	r(C2−S1)	1.7771	1.7828	1.7784
	r(C2−C3)	1.3983	1.3989	1.3963

r(C3−C4)	1.3890	1.3911	1.3890
r(C4−C5)	1.3914	1.3945	1.3919
r(C5−C6)	1.3903	1.3926	1.3907
r(C6−C7)	1.3915	1.3941	1.3909
r(C7−C2)	1.3942	1.3932	1.3920
r(C3−H8)	1.0829	1.0847	1.0876
r(C4−H9)	1.0815	1.0836	1.0868
r(C5−H10)	1.0810	1.0831	1.0862
r(C6−H11)	1.0816	1.0837	1.0869
r(C7−H12)	1.0815	1.0827	1.0859
α(C2S1S1)	105.05	105.53	105.49
α(S1C2C3)	115.92	115.61	115.82
α(C2C3C4)	120.44	119.82	119.79
α(C3C4C5)	120.01	120.29	120.36
α(C4C5C6)	119.59	119.55	119.49
α(C5C6C7)	120.63	120.66	120.69
α(C2C3H8)	119.95	119.99	120.00
α(C3C4H9)	119.49	119.50	119.43
α(C4C5H10)	120.21	120.21	120.25
α(C5C6H11)	120.10	120.09	120.09
α(C6C7H12)	120.58	120.34	120.41
δ(C2S1S1C2)	-81.21	-84.55	-86.32
δ(S1S1C2C3)	-167.29	-168.48	-163.87
δ(S1C2C3C4)	-177.54	-178.22	-177.54
δ(C2C3C4C5)	-0.58	-0.24	-0.43
δ(C3C4C5C6)	0.08	0.11	0.18
δ(C4C5C6C7)	0.64	0.23	0.30
δ(S1C2C3H8)	2.48	2.15	2.92
δ(C2C3C4H9)	-179.69	-179.79	-179.79
δ(C3C4C5H10)	-179.74	-179.77	-179.62
δ(C4C5C6H11)	180.00	179.97	-179.95
δ(C5C6C7H12)	-179.76	-179.89	-179.98

[a] Bond lengths and angles in Å and °, respectively. See Figures 1 - 6 of the manuscript for atom labelling.

Table S2: Statistics and LRA parameters for valence angles for rev-DSDPBEP86/jun-cc-pV(T+d)Z and PW6B95/jul-cc-pV(D+d)Z levels of theory[a]

Parameter	rev-DSDPBEP86	PW6B95	rev-DSDPBEP86-LRA	PW6B95-LRA
CCC angle, $N = 56$				
MD	0.03	0.11	1×10^{-4}	-0.02
Neg.	-0.61	-0.65	-0.65	-0.79
Pos.	0.39	3.0	0.36	2.81
MAD	0.12	0.24	0.11	0.26
A	-0.00025	-0.00112	–	–
B	0	0	–	–
CCH angle, $N = 159$				
MD	0.001	0.032	-6×10^{-5}	-8×10^{-4}
Neg.	-1.21	-1.21	-1.22	-1.24
Pos.	1.48	1.51	1.48	1.48
MAD	0.16	0.18	0.16	0.18
A	-0.00001	-0.00027	–	–
B	0	0	–	–
HCH angle, $N = 54$				
MD	-0.15	-0.33	4×10^{-4}	-2×10^{-4}
Neg.	-0.58	-0.90	-0.39	-0.64
Pos.	0.33	0.35	0.37	0.70
MAD	0.17	0.38	0.09	0.22
A	0.01695	0.02077	–	–
B	-1.77589	-2.01637	–	–
CCO angle, $N = 31$				
MD	0.09	0.04	0.002	-0.01
Neg.	-0.24	-0.42	-0.32	-0.47
Pos.	0.34	0.65	0.25	0.57
MAD	0.14	0.21	0.12	0.21
A	-0.0007	-0.00047	–	–
B	0	0	–	–
COC angle, $N = 7$				
MD	0.12	0.35	–	-1×10^{-4}
Neg.	-0.08	–	–	-0.32
Pos.	0.36	0.78	–	0.40
MAD	0.14	0.35	–	0.14
A	0	-0.00311	–	–
B	0	-0.02646	–	–
OCO angle, $N = 7$				
MD	0.12	-0.08	-4×10^{-4}	-9×10^{-5}
Neg.	-0.03	-0.30	-0.05	-0.22
Pos.	0.25	0.11	0.08	0.19
MAD	0.13	0.13	0.03	0.10
A	-0.06709	0.00063	–	–
B	8.1676	0	–	–
HCO angle, $N = 27$				
MD	-0.01	0.05	-5×10^{-4}	0.002
Neg.	-0.42	-0.63	-0.41	-0.68
Pos.	0.14	0.32	0.14	0.27
MAD	0.08	0.12	0.08	0.12
A	0.00011	-0.00044	–	–
B	0	0	–	–
CCN angle, $N = 21$				
MD	-0.004	-0.06	–	–

Neg.	-0.27	-0.39	–	–
Pos.	0.13	0.20	–	–
MAD	0.07	0.11	–	–
A	0	0	–	–
B	0	0	–	–
CNC angle, $N = 13$				
MD	0.07	0.23	–	-4×10^{-4}
Neg.	-0.04	–	–	-0.14
Pos.	0.29	0.36	–	0.12
MAD	0.08	0.23	–	0.07
A	0	0.0035	–	–
B	0	-0.6132	–	–
HCN angle, $N = 31$				
MD	0.005	0.17	-9×10^{-4}	-2×10^{-4}
Neg.	-0.62	-0.39	-0.63	-0.57
Pos.	0.33	0.59	0.33	0.42
MAD	0.16	0.22	0.16	0.15
A	-0.00003	-0.0014	–	–
B	0	0	–	–
CSC angle, $N = 6$				
MD	0.05	0.28	-0.01	-0.03
Neg.	-0.07	–	-0.11	-0.27
Pos.	0.18	0.75	0.11	0.40
MAD	0.08	0.28	0.06	0.24
A	-0.00073	-0.00359	–	–
B	0	0	–	–
SCH angle, $N = 17$				
MD	0.11	0.12	-0.004	-0.004
Neg.	-0.16	-0.41	-0.26	-0.52
Pos.	0.67	0.59	0.55	0.48
MAD	0.16	0.30	0.15	0.29
A	-0.00095	-0.00105	–	–
B	0	0	–	–
CCS angle, $N = 7$				
MD	-0.06	0.04	–	–
Neg.	-0.31	-0.11	–	–
Pos.	0.06	0.27	–	–
MAD	0.09	0.13	–	–
A	0	0	–	–
B	0	0	–	–
CNH angle, $N = 11$				
MD	0.21	-9×10^{-4}	-0.02	0.01
Neg.	-0.10	-0.69	-0.34	-0.67
Pos.	1.57	0.78	1.32	0.79
MAD	0.27	0.22	0.32	0.22
A	-0.00195	0.00011	–	–
B	0	0	–	–

COH angle, $N = 8$				
MD	-0.011	0.31	–	-4×10^{-4}
Neg.	-0.20	-0.08	–	-0.15
Pos.	0.12	0.66	–	0.23
MAD	0.09	0.34	–	0.09
A	0	-0.16466	–	–
B	0	17.43968	–	–

[a] N: number of points in the linear fit; MD: Mean Deviation (deg); Neg.: largest negative error (deg); Pos.: largest positive error (deg); MAD: Mean Absolute Deviation (deg); B (or A) = 0 means that the parameter has been fixed to zero.

Table S3: Theoretical equilibrium geometries of proline at rev-DSDPBEP86/jun-cc-pVTZ, PW6B95/jul-cc-pVDZ levels of theory, upon LRA and TMA augmentation, and comparison to the SE equilibrium structure[a]

	**SE**	**rev-DSD**	**PW6**	**rev-DSD-LRA**	**PW6-LRA**	**rev-DSD-TMA**	**PW6-TMA**
N1H9	1.0078	1.0107	1.0098	1.0085	1.0064	1.0081	1.0068
N1C2	1.4785	1.4805	1.4738	1.4769	1.4781	1.4757	1.4756
C2C6	1.5321	1.5367	1.5314	1.5339	1.5316	1.5318	1.5328
C6O8	1.3343	1.3375	1.3322	1.3336	1.338	1.3334	1.3332
O8H11	0.9861	0.9864	0.9851	0.9889	0.9877	0.9829	0.9838
C6O7	1.2027	1.2061	1.2043	1.2025	1.2036	1.2023	1.2019
C2H10	1.0891	1.0918	1.0943	1.0892	1.0878	1.0895	1.0889
C2C3	1.5307	1.5366	1.5322	1.5338	1.5325	1.5325	1.5299
C3H12	1.0903	1.0929	1.0949	1.0903	1.0885	1.0903[b]	1.0885[b]
C3H13	1.0865	1.0888	1.0913	1.0862	1.0849	1.0862[b]	1.0849[b]
C3C4	1.5262	1.5305	1.5249	1.5276	1.5251	1.5287	1.5273
C4H14	1.0872	1.0899	1.0924	1.0873	1.0859	1.0873[b]	1.0859[b]
C4H15	1.0909	1.0935	1.0960	1.0909	1.0896	1.0909[b]	1.0896[b]
C4C5	1.5215	1.5262	1.5209	1.5233	1.5211	1.5220	1.5185
C5H16	1.0931	1.0961	1.0992	1.0935	1.0927	1.0935[b]	1.0927[b]
C5H17	1.0880	1.0908	1.939	1.0882	1.0875	1.0882[b]	1.0875[b]
MD		0.0031	0.0021	0.0006	-0.0005	0.0003	-0.0012
MAD		0.0031	0.0031	0.0010	0.0010	0.0011	0.0014
C2N1H9	111.84	111.75	112.14	111.53	112.16	111.83	112.19
C6C2N1	110.09	110.21	110.21	110.21	110.21	110.10	110.00
C2C6O8	114.17	113.94	113.86	113.86	113.81	114.13	113.56
C6O8H11	102.88	103.50	103.68	103.50	104.05	103.56	103.38
C2C6O7	122.41	122.60	122.66	122.52	122.61	122.45	122.80
N1C2C10	112.03	112.00	112.05	112.00	111.90	112.00[b]	111.90[b]
N1C2C3	105.85	105.73	105.73	105.73	105.73	105.86	105.81
C2C3H12	109.88	109.70	109.76	109.70	109.74	109.70[b]	109.74[b]
C2C3H13	111.69	111.69	111.51	111.69	111.48	111.69[b]	111.48[b]
C2C3C4	102.279	102.38	102.54	102.36	102.42	102.35	102.53
C3C4H14	113.53	113.38	113.33	113.37	113.30	113.37[b]	113.30[b]
C3C4H15	110.16	110.30	110.41	110.30	110.38	110.30[b]	110.38[b]
C3C4C5	102.221	102.17	102.32	102.14	102.21	102.13	102.32
C4C5H16	109.93	110.00	110.07	110.00	110.04	110.00[b]	110.04[b]
C4C5H16	113.25	113.27	113.21	113.27	113.18	113.27[b]	113.18[b]
MD		0.03	0.09	-0.002	0.07	0.04	0.03
MAD		0.14	0.23	0.16	0.24	0.10	0.23
C6C2N1H9	-119.5	-117.86	-116.08	n.a.	n.a.	n.a.	n.a.
N1C2C6O8	2.78	2.06	2.23	n.a.	n.a.	n.a.	n.a.
C2C6O8H11	-0.35	-0.56	-1.08	n.a.	n.a.	n.a.	n.a.
N1C2C6O7	-177.09	-177.88	-177.62	n.a.	n.a.	n.a.	n.a.
H9N1C2H10	-0.01	0.27	1.91	n.a.	n.a.	n.a.	n.a.
H9N1C2C3	120.85	122.24	123.74	n.a.	n.a.	n.a.	n.a.
N1C2C3H12	-90.78	-90.62	-91.84	n.a.	n.a.	n.a.	n.a.
N1C2C3H13	148.93	149.03	148.25	n.a.	n.a.	n.a.	n.a.
N1C2C3C4	26.58	26.11	25.27	n.a.	n.a.	n.a.	n.a.
C2C3C4H14	-161.58	-161.26	-160.58	n.a.	n.a.	n.a.	n.a.
C2C3C4H15	77.17	77.14	78.16	n.a.	n.a.	n.a.	n.a.
C2C3C4C5	-40.1	-39.95	-39.20	n.a.	n.a.	n.a.	n.a.
C3C4C5H16	-79.57	-79.83	-80.44	n.a.	n.a.	n.a.	n.a.
C3C4C5H17	159.01	158.78	158.58	n.a.	n.a.	n.a.	n.a.

MD	0.09	0.35	–	–	–	–
MAD	0.48	1.23	–	–	–	–

[a] Bond lengths in Å and angles in deg. MD = Mean Deviation; MAD = Mean Absolute Deviation. SE equilibrium geometry from Ref.[S1]

[b] Fixed to the corresponding LRA value.

Table S4: Theoretical equilibrium geometries of 2-deoxyribose at rev-DSDPBEP86/jun-cc-pVTZ, PW6B95/jul-cc-pVDZ levels of theory, and upon LRA augmentation, and comparison to the SE equilibrium structure[a]

	SE	rev-DSDPBEP86	PW6B95	rev-DSDPBEP86-LRA	PW6B95-LRA
C1C2	1.5182	1.5218	1.5167	1.5190	1.5169
C2C3	1.5258	1.5293	1.5253	1.5265	1.5255
C3C4	1.5226	1.5287	1.5251	1.5259	1.5253
C4C5	1.5133	1.5178	1.5130	1.5150	1.5132
C1O6	1.4183	1.4204	1.4179	1.4162	1.4209
C1O7	1.4072	1.4099	1.4054	1.4057	1.4082
C3O8	1.4145	1.4172	1.4118	1.4130	1.4147
C4O9	1.4262	1.4294	1.4249	1.4251	1.4280
C1H10	1.0905	1.0930	1.0962	1.0904	1.0898
C2H11	1.0906	1.0936	1.0962	1.0910	1.0897
C2H12	1.08789	1.0905	1.0924	1.0879	1.0860
C3H13	1.08969	1.0923	1.0950	1.0897	1.0886
C4H14	1.0897	1.0923	1.0946	1.0896	1.0881
C5H15	1.08719	1.0897	1.0922	1.0871	1.0858
C5H16	1.0909	1.0937	1.0962	1.0911	1.0898
O7H17	0.96041	0.9633	0.9610	0.9601	0.9594
O8H18	0.9633	0.9659	0.9645	0.9633	0.9635
O9H19	0.96239	0.9648	0.9635	0.9619	0.9623
MD		0.0030	0.0018	0.0005	-0.0002
MAD		0.0030	0.0028	0.0010	0.0011
C1C2C3	111.747	111.82	111.83	111.79	111.70
C2C3C4	109.559	109.83	109.63	109.80	109.51
C3C4C5	110.107	109.80	109.93	109.77	109.81
O6C1C2	111.775	111.72	111.53	111.64	111.48
O6C1O7	111.47	111.47	111.36	n.a.	n.a.
C2C3O8	111.33	111.33	111.35	111.25	111.30
C3C4O9	109.43	109.93	109.44	109.86	109.39
O6C1H10	104.09	103.94	104.03	103.92	103.98
C1C2H11	108.36	108.28	108.25	108.28	108.22
C1C2H12	109.79	109.81	109.94	109.81	109.91
C2C3H13	109.56	109.68	109.81	109.68	109.78
C3C4H14	109.46	109.48	109.61	109.48	109.58
C4C5H15	110.61	110.77	111.01	110.77	110.98
C4C5H16	110.30	110.24	110.31	110.24	110.28
C1O7H17	107.58	107.79	108.31	107.79	107.92
C3O8H18	105.67	106.07	106.10	106.07	106.07
C4O9H19	106.72	107.03	107.01	107.03	106.83
MD		0.09	0.11	0.07	0.03
MAD		0.16	0.19	0.16	0.17
C1C2C3C4	49.67	49.95	49.96	n.a.	n.a.
C2C3C4C5	-53.26	-53.39	-53.53	n.a.	n.a.
C5O6C1C2	58.93	58.20	58.83	n.a.	n.a.
O6C1C2C3	-51.98	-51.89	-52.19	n.a.	n.a.
C1C2C3O3	172.65	173.06	172.58	n.a.	n.a.
C2C3C4O4	69.21	69.17	68.57	n.a.	n.a.
C5O6C1H1	178.54	178.26	178.92	n.a.	n.a.
O6C1C2H2q	-174.80	-174.51	-175.08	n.a.	n.a.
O6C1C2H2x	67.79	68.14	67.78	n.a.	n.a.
C1C2C3H3	-69.43	-69.38	-69.54	n.a.	n.a.
C2C3C4H4	-174.55	-174.60	-175.22	n.a.	n.a.

C3C4C5H5q	175.88	175.79	176.36	n.a.	n.a.
C3C4C5H5x	-63.17	-63.08	-62.36	n.a.	n.a.
O6C1O1H1	-61.01	-61.45	-61.67	n.a.	n.a.
C2C3O3H3	-78.67	-78.84	-78.38	n.a.	n.a.
C3C4O4H4	-86.12	-85.93	-85.13	n.a.	n.a.
MD		-0.01	0.01	n.a.	n.a.
MAD		0.23	0.39	n.a.	n.a.

a Bond lengths in Å and angles in deg. MD = Mean Deviation; MAD = Mean Absolute Deviation. SE equilibrium geometry from Ref.[S2]

References

(S1) Vogt, N.; Demaison, J.; Krasnoshchekov, S. V.; Stepanov, N. F.; Rudolph, H. D. Determination of accurate semiexperimental equilibrium structure of proline using efficient transformations of anharmonic force fields among the series of isotopologues. *Mol. Phys.* **2017**, *115*, 942–951.

(S2) Vogt, N.; Demaison, J.; Cocinero, E. J.; Ècija, P.; Lesarri, A.; Rudolph, H. D.; Vogt, J. The equilibrium molecular structures of 2-deoxyribose and fructose by the semiexperimental mixed estimation method and coupled-cluster computations. *Phys. Chem. Chem. Phys.* **2016**, *18*, 15555–15563.

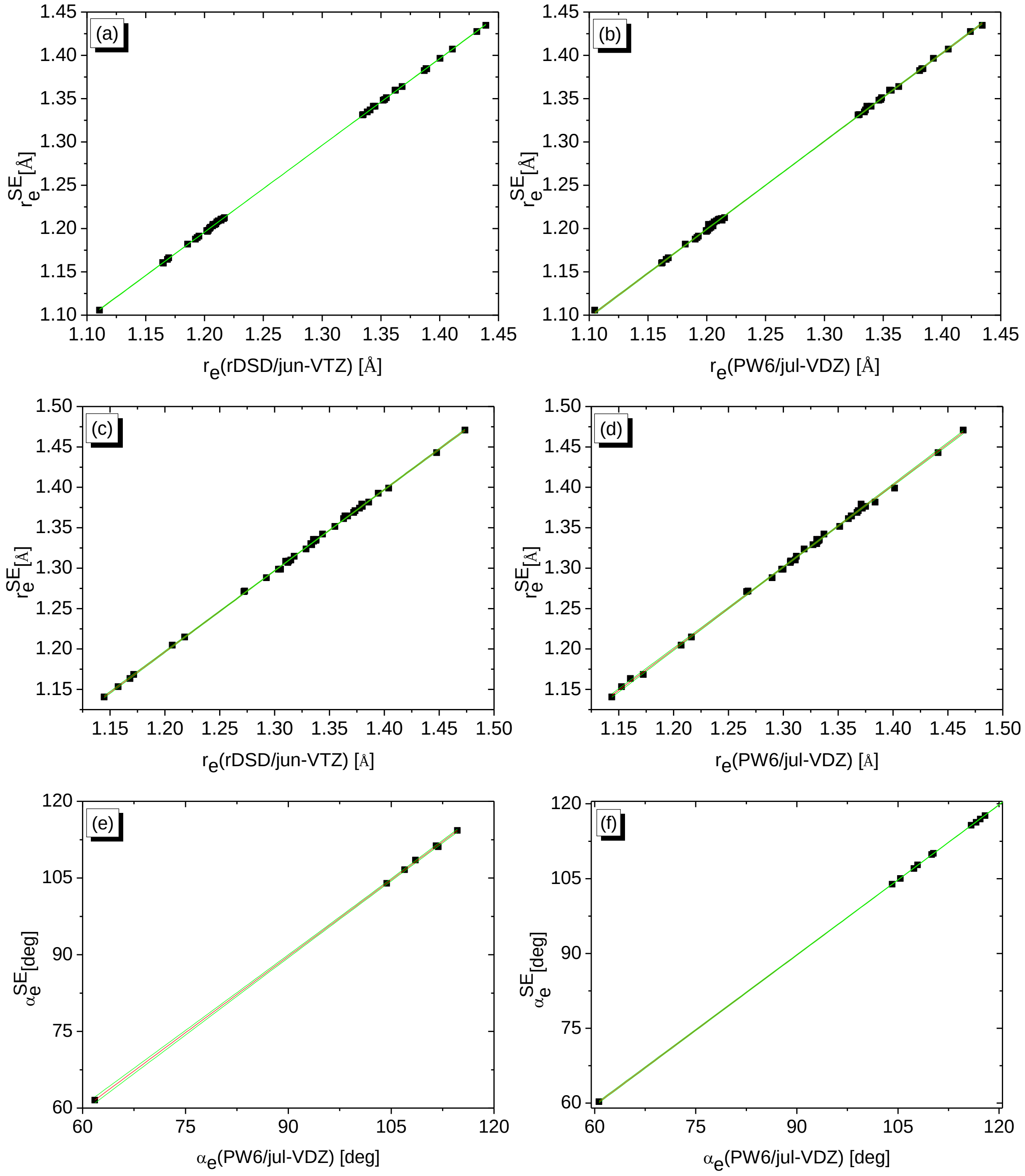

Figure S1: LRA for CO bond lengths at (a) rev-DSDPBEP86/jun-cc-pVTZ and (b) PW6B95/jul-cc-pVDZ levels of theory; for CN bond lengths at (c) rev-DSDPBEP86/jun-cc-pVTZ and (d) PW6B95/jul-cc-pVDZ levels of theory; for COC (e) and CNC (f) angles at PW6B95/jul-cc-pVDZ levels of theory. Green curves represents 95% confidence intervals.

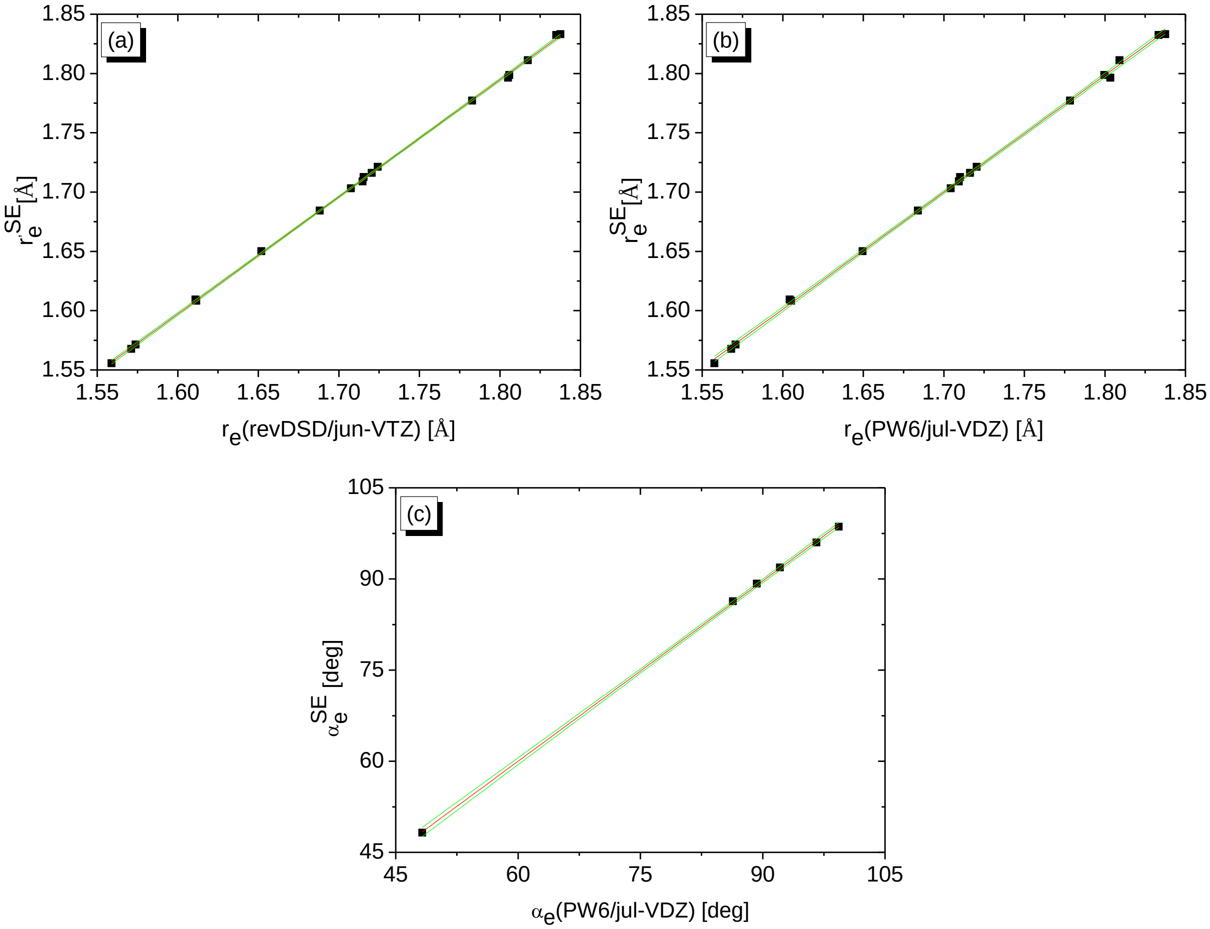

Figure S2: LRA for CS bond length at (a) rev-DSDPBEP86/jun-cc-pV(T+d)Z and (b) PW6B95/jul-cc-pV(D+d)Z levels of theory; (c) for CSC angle at PW6B95/jul-cc-pVDZ levels of theory. Green curves represent 95% confidence intervals.

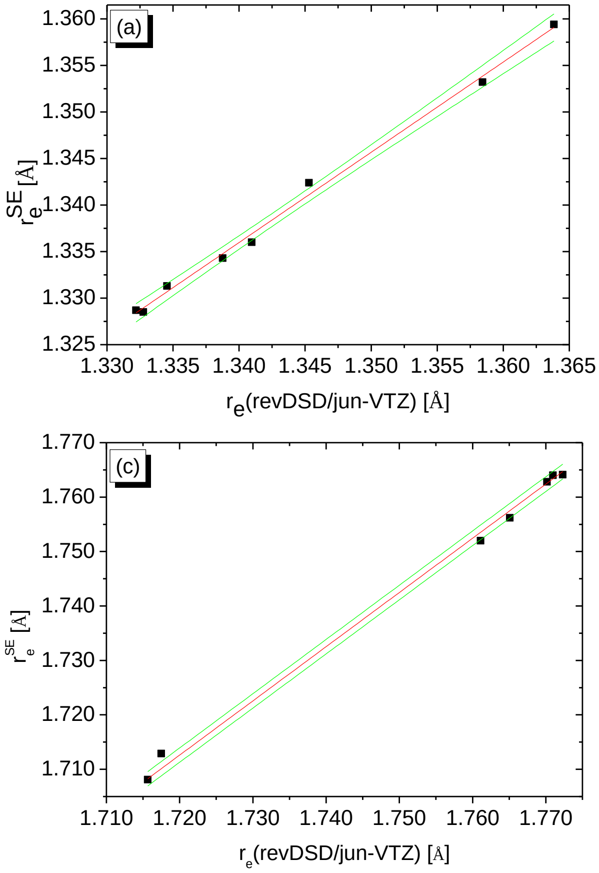

Figure S3: LRA for C−F bond length at (a) rev-DSDPBEP86/jun-cc-pVTZ and (b) PW6B95/jul-cc-pVDZ levels of theory; (c) for C−Cl bond length (c) rev-DSDPBEP86/jun-cc-pV(T+d)Z and (d) PW6B95/jul-cc-pV(D+d)Z level of theory. Green curves represent 95% confidence intervals.

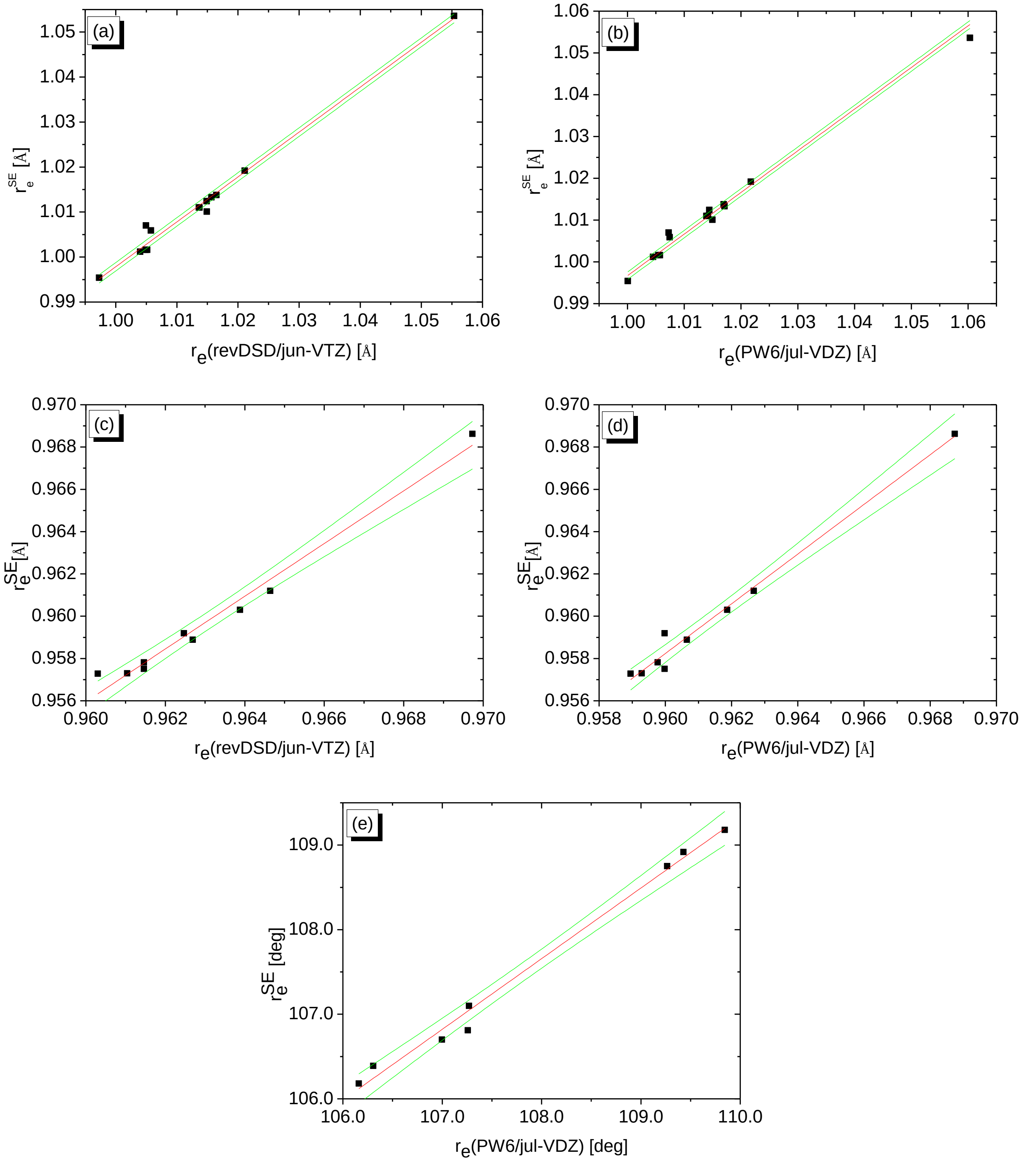

Figure S4: LRA for N−H bond length at (a) rev-DSDPBEP86/jun-cc-pVTZ and (b) PW6B95/jul-cc-pVDZ levels of theory; (c) for O−H bond length (c) rev-DSDPBEP86/jun-cc-pVTZ and (d) PW6B95/jul-cc-pVDZ level of theory; (e) for COH bond angle at PW6B95/jul-cc-pVDZ level of theory. Green curves represent 95% confidence intervals.

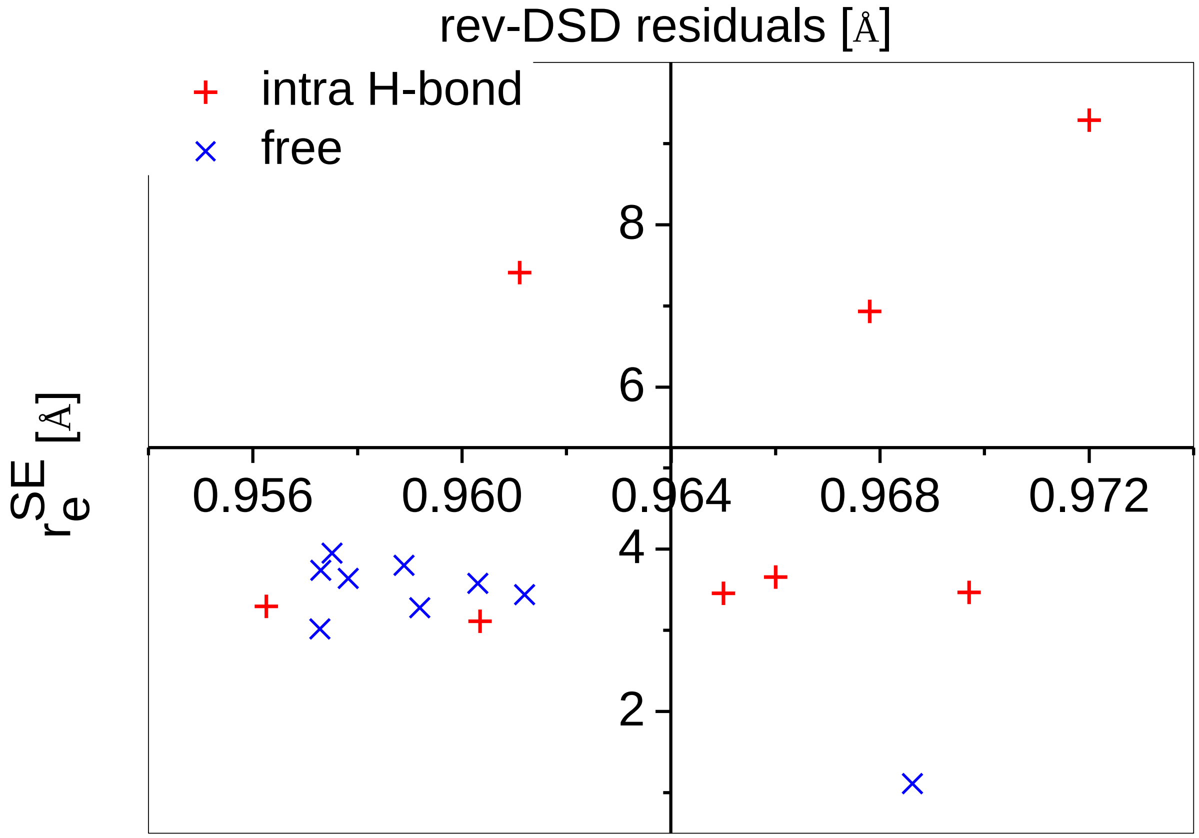

Figure S5: Residuals of OH bond distances computed at rev-DSDPBEP86/jun-cc-pVTZ from SE equilibrium values.
